# Supplementary material for: ACLY ubiquitination by CUL3-KLHL25 induces the reprogramming of fatty acid metabolism to facilitate iTreg differentiation
Source: eLife. 2021 Sep 7;10:e62394. doi: 10.7554/eLife.62394 (PMC8423445; doi:10.7554/eLife.62394)
Supplement: Source data 1. [file elife-62394-data1.zip › Source data files/Source data (Raw)/新建 Microsoft PowerPoint 演示文稿.pptx]

## Slide 1
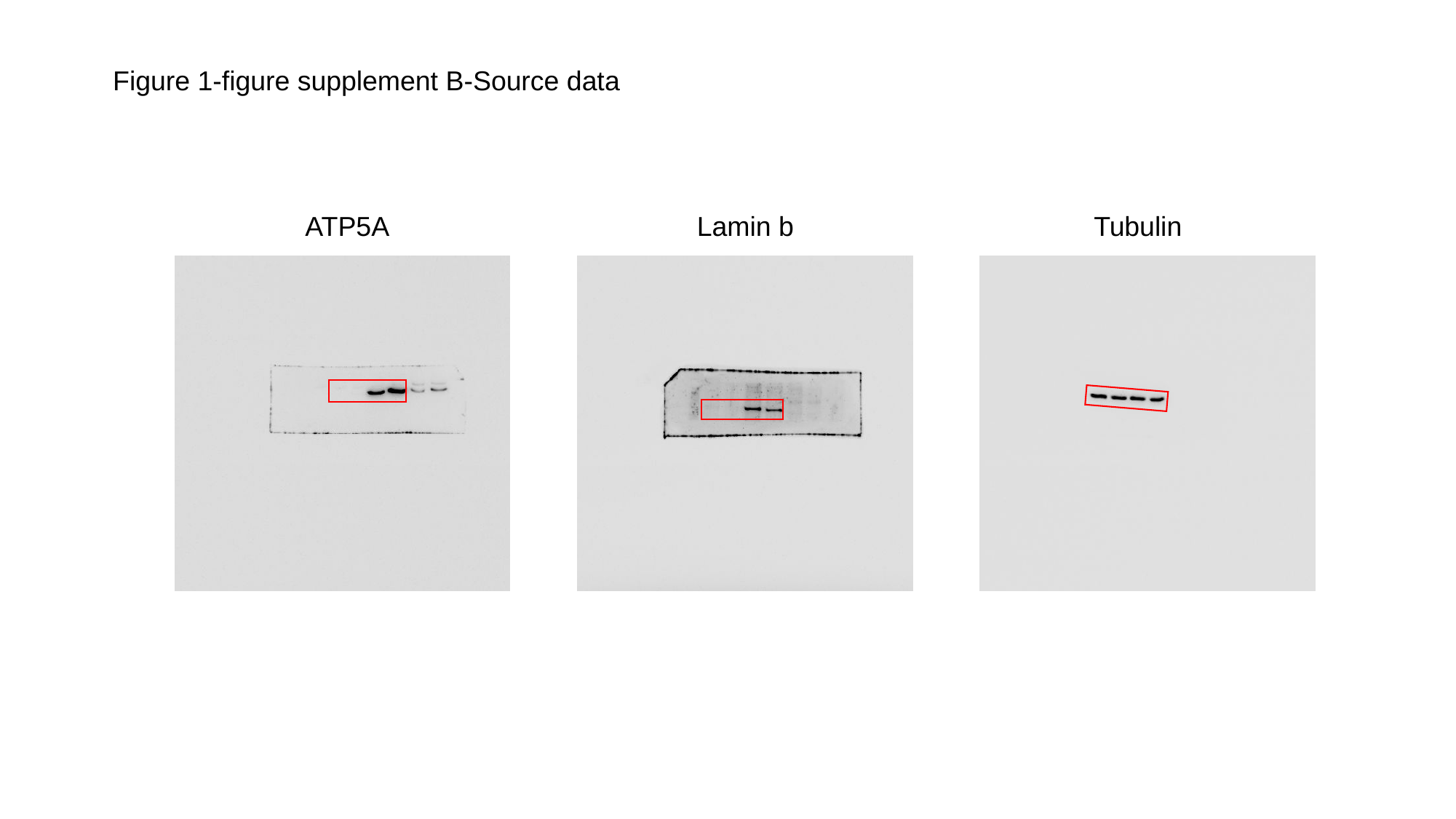

Figure 1-figure supplement B-Source data
Tubulin
ATP5A
Lamin b

## Slide 2
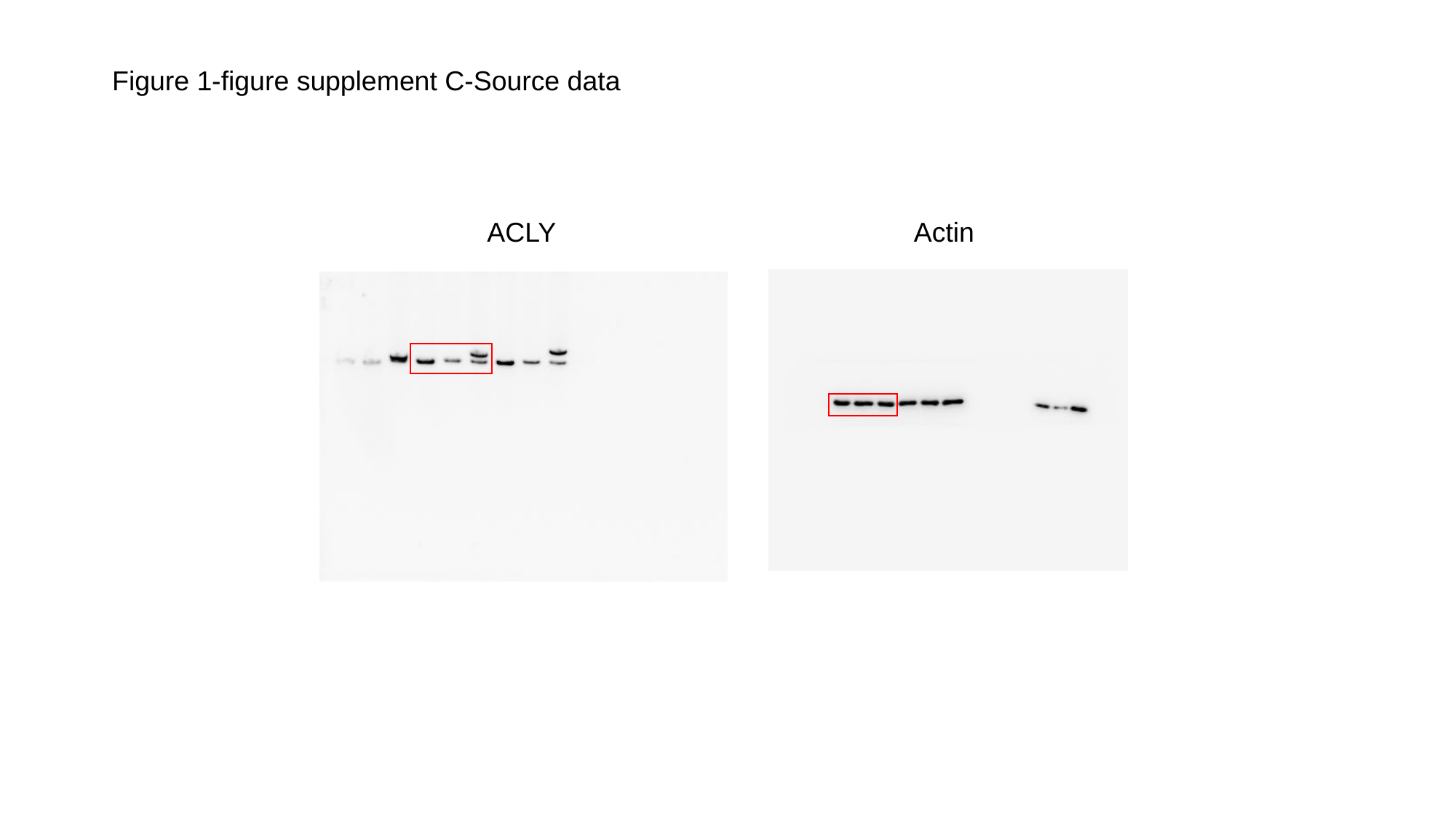

Figure 1-figure supplement C-Source data
ACLY
Actin

## Slide 3
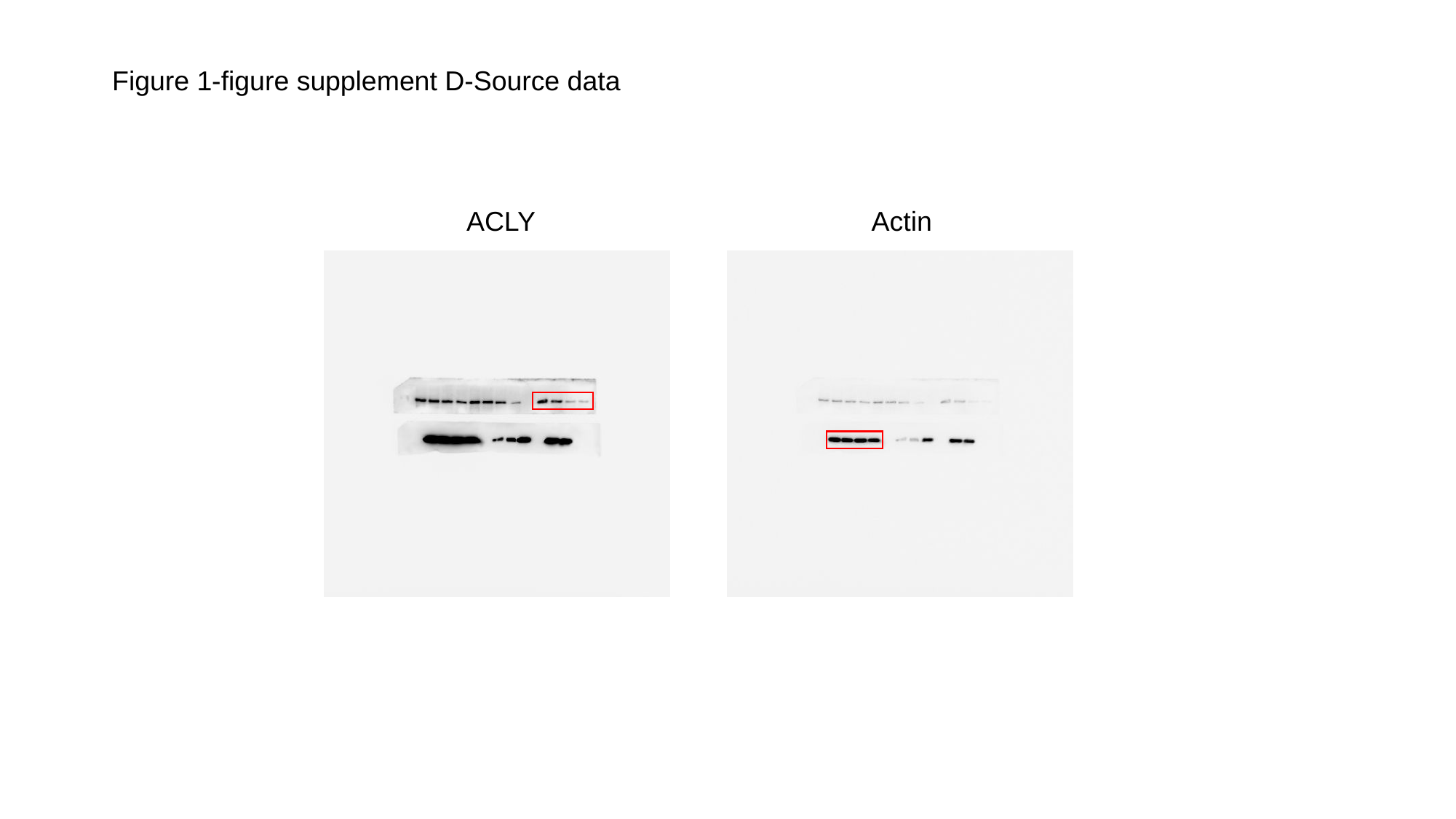

Figure 1-figure supplement D-Source data
ACLY
Actin

## Slide 4
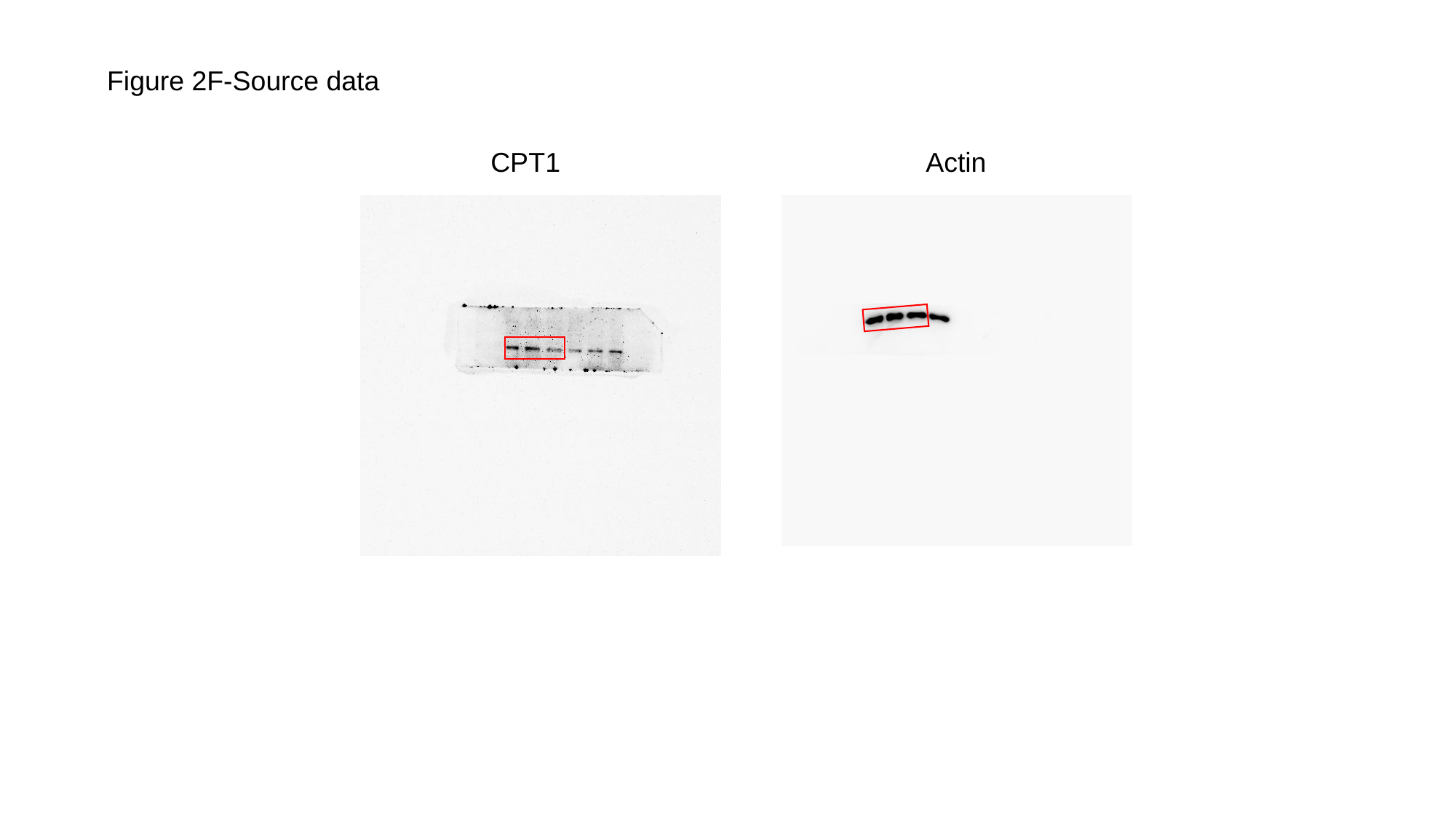

Figure 2F-Source data
CPT1
Actin

## Slide 5
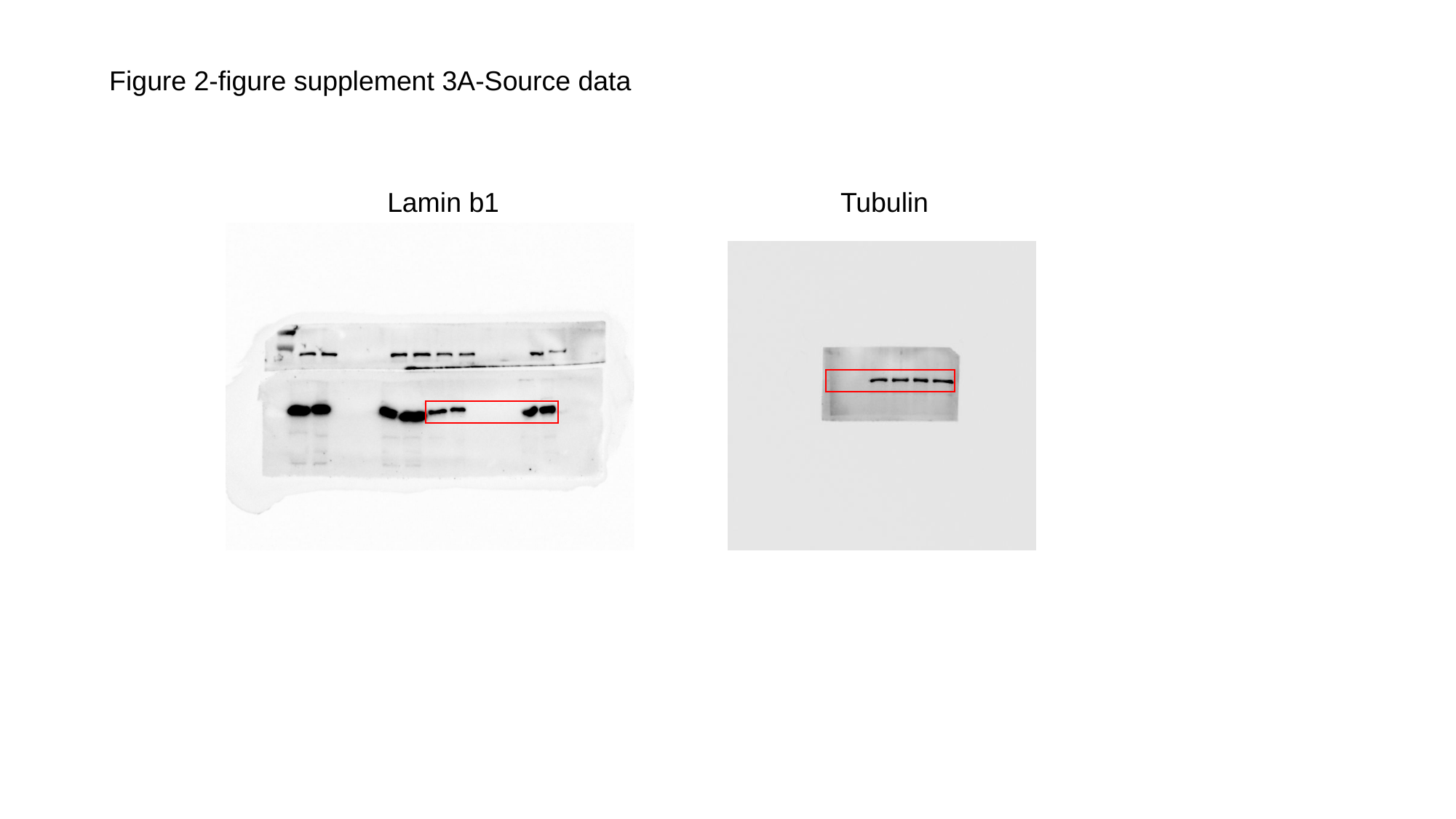

Figure 2-figure supplement 3A-Source data
Lamin b1
Tubulin

## Slide 6
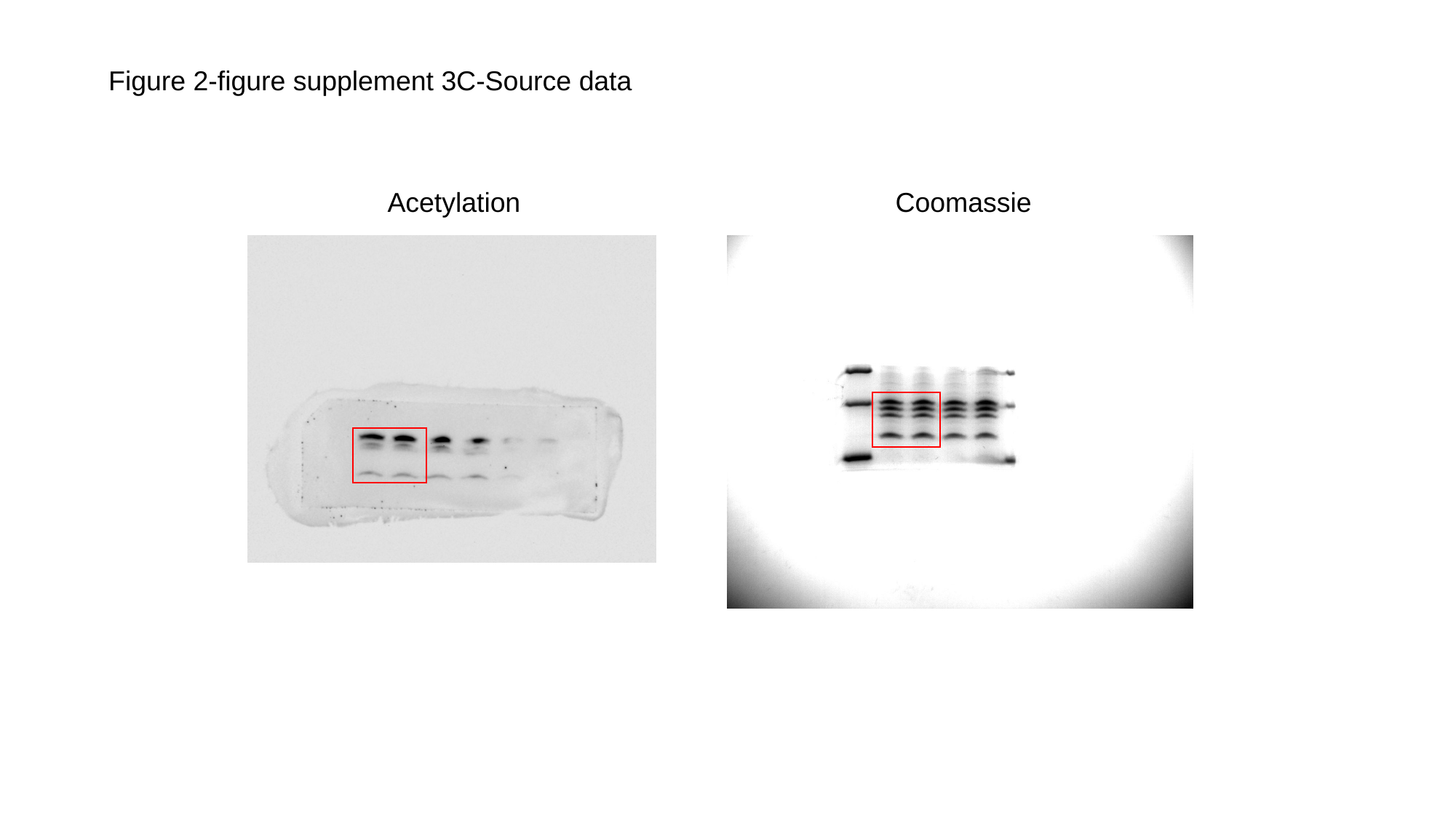

Figure 2-figure supplement 3C-Source data
Acetylation
Coomassie

## Slide 7
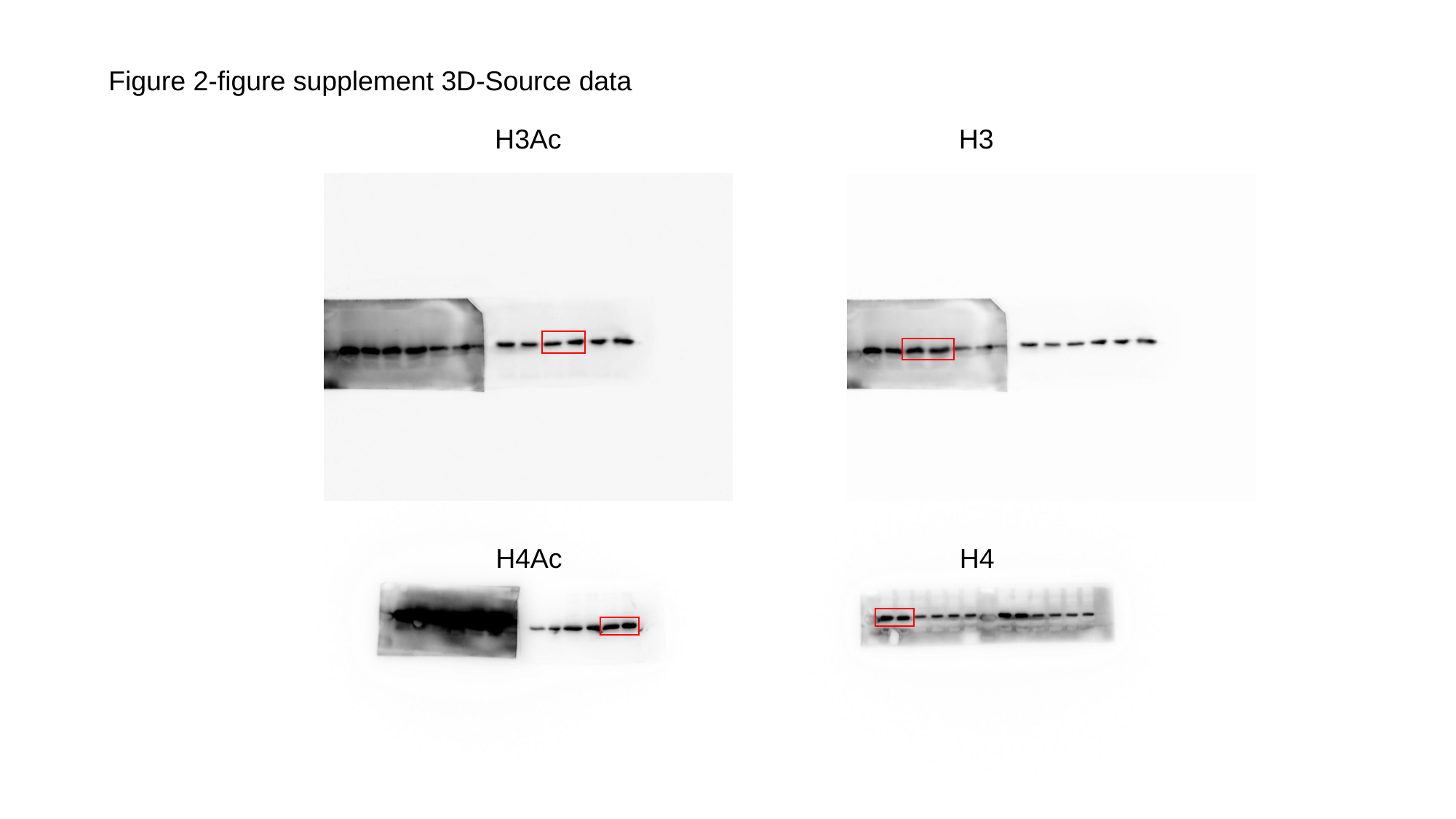

Figure 2-figure supplement 3D-Source data
H3Ac
H3
H4Ac
H4

## Slide 8
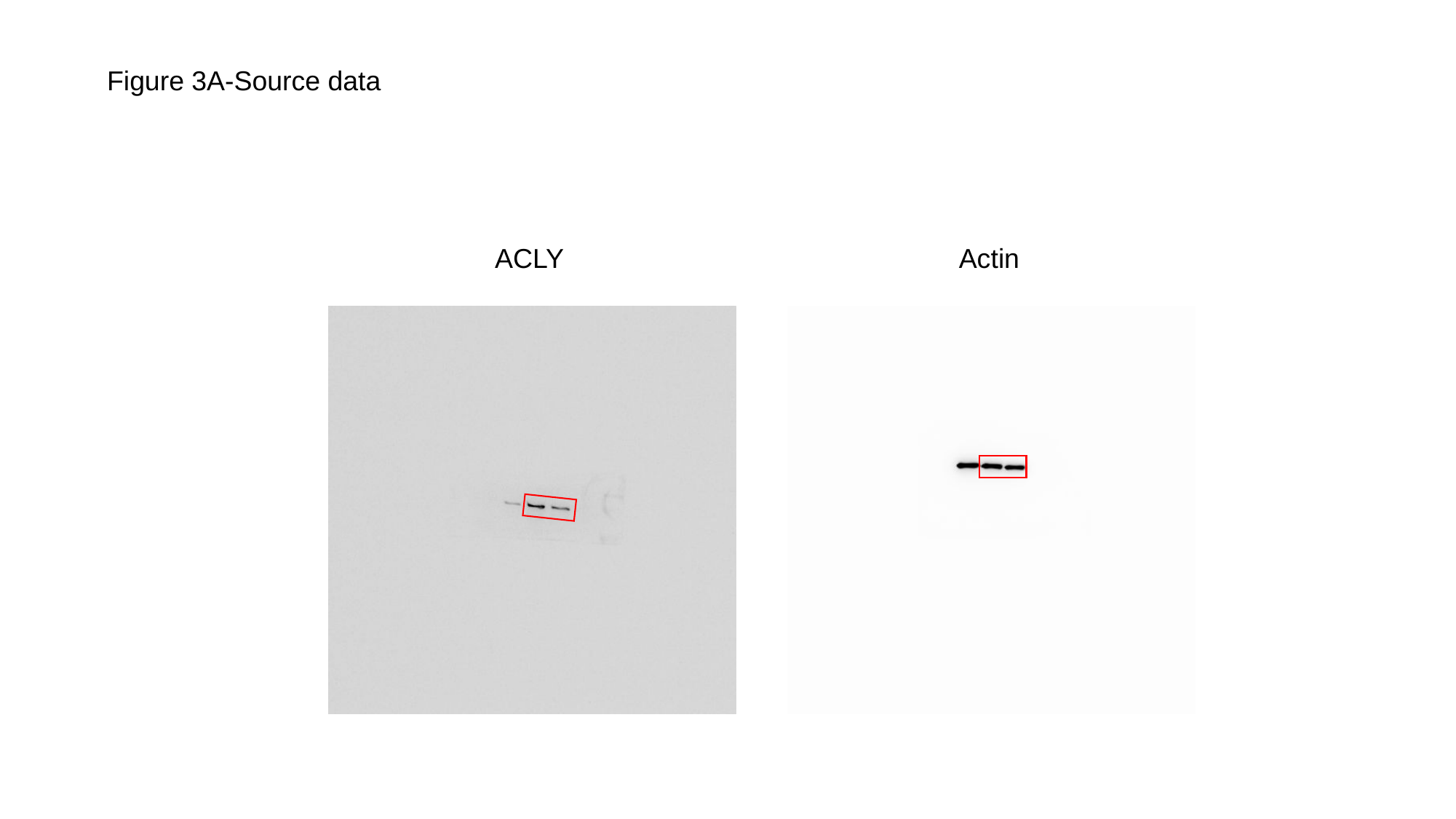

Figure 3A-Source data
ACLY
Actin

## Slide 9
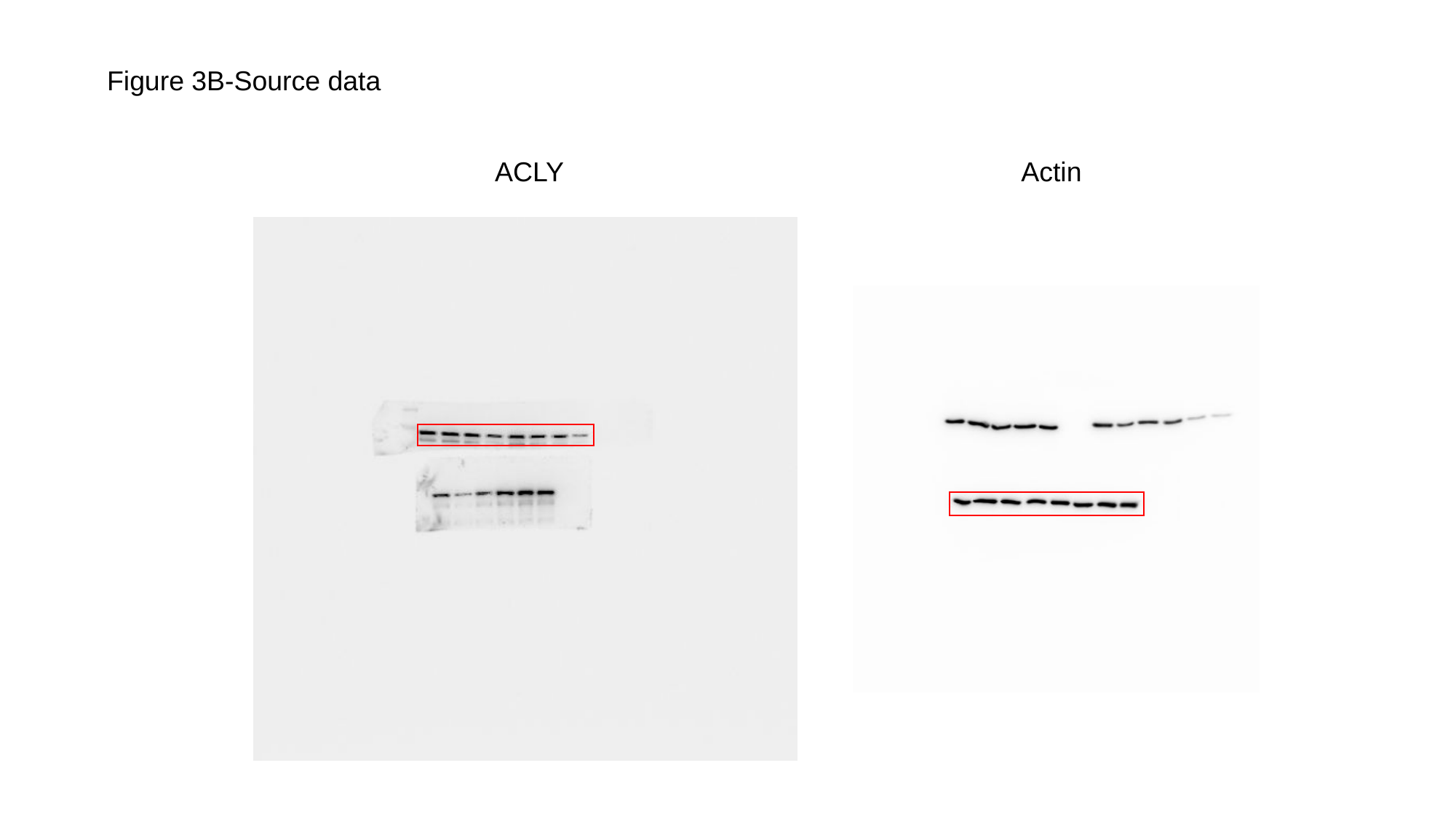

Figure 3B-Source data
ACLY
Actin

## Slide 10
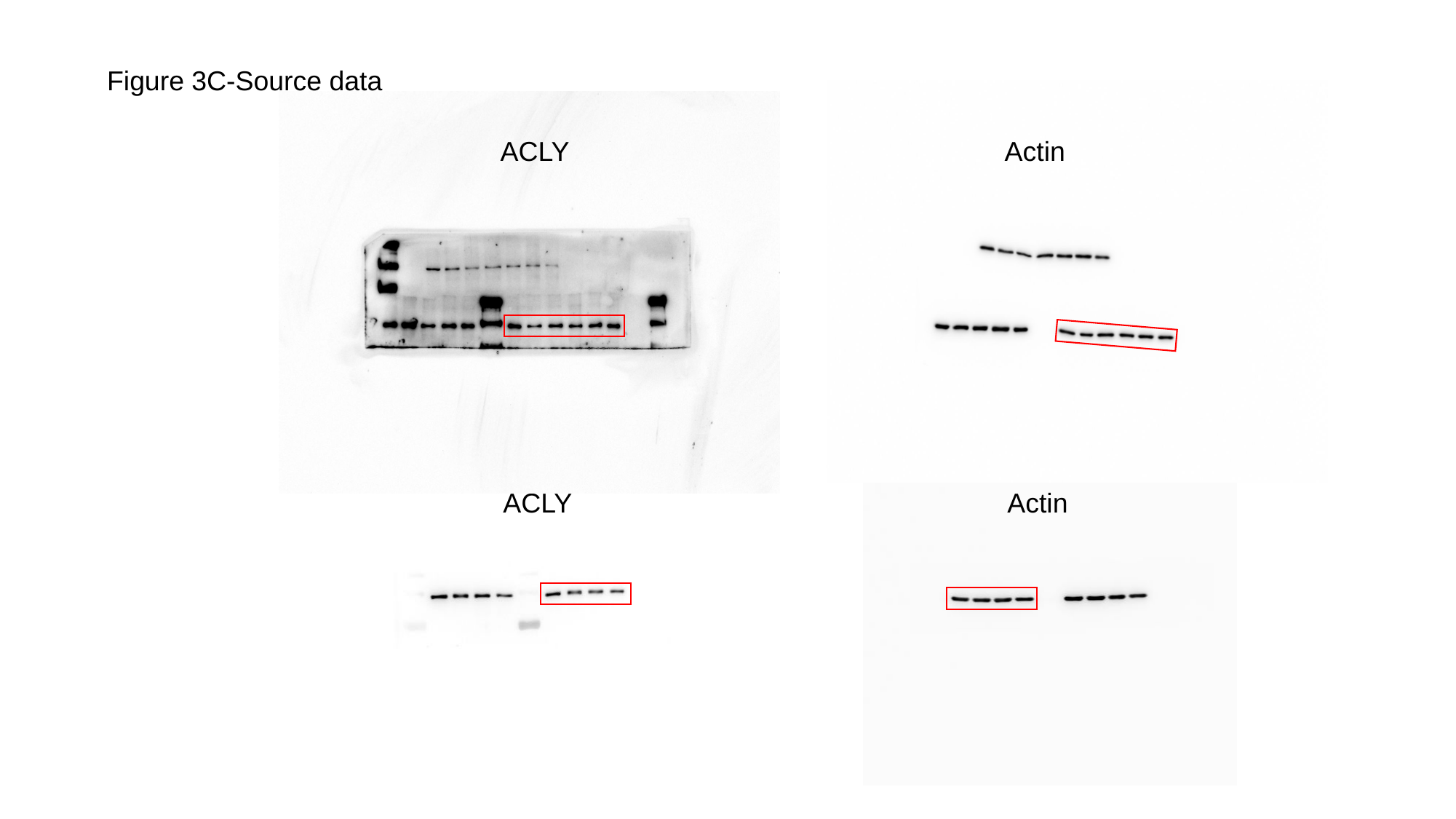

Figure 3C-Source data
ACLY
Actin
ACLY
Actin

## Slide 11
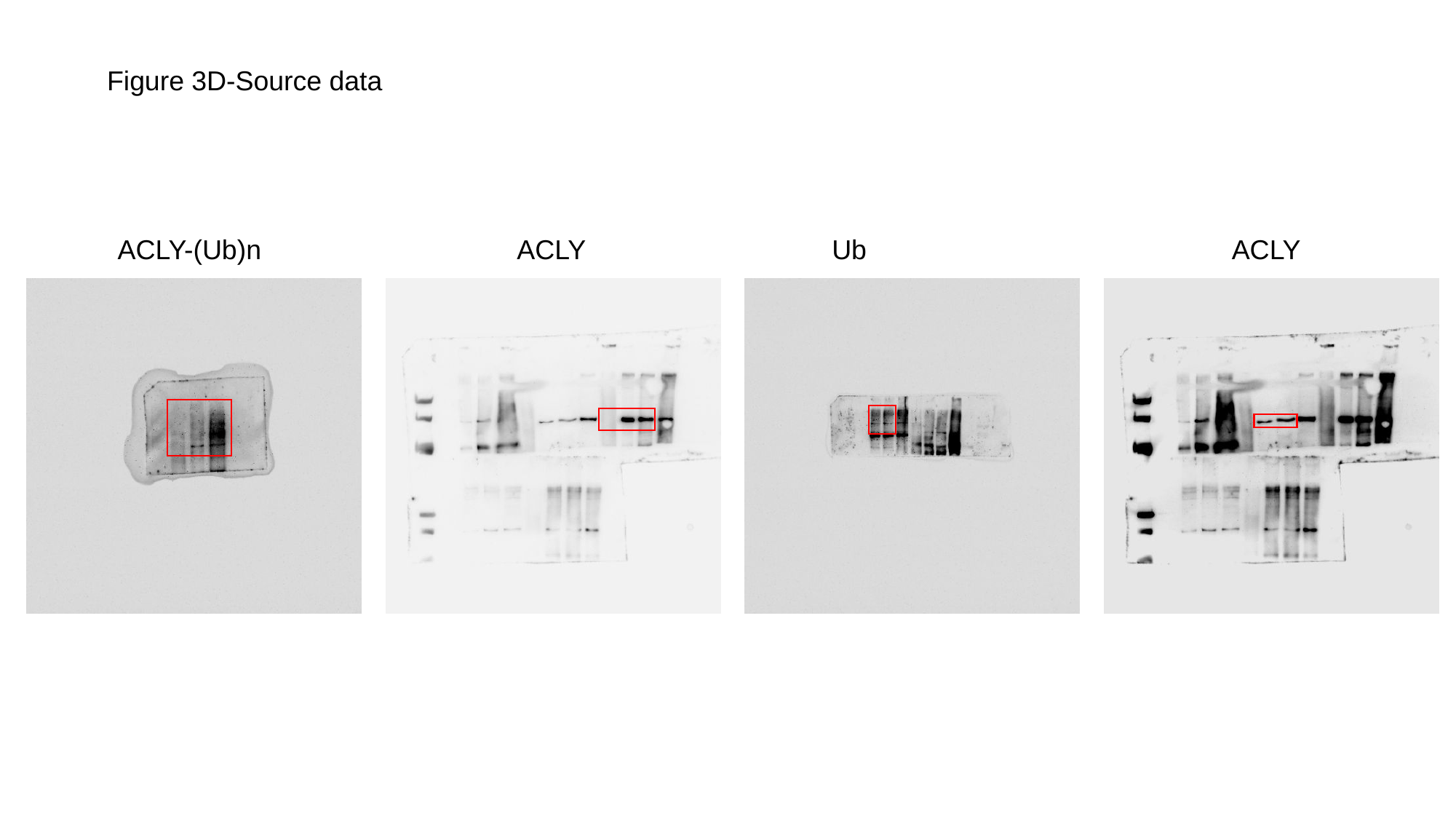

Figure 3D-Source data
ACLY-(Ub)n
ACLY
Ub
ACLY

## Slide 12
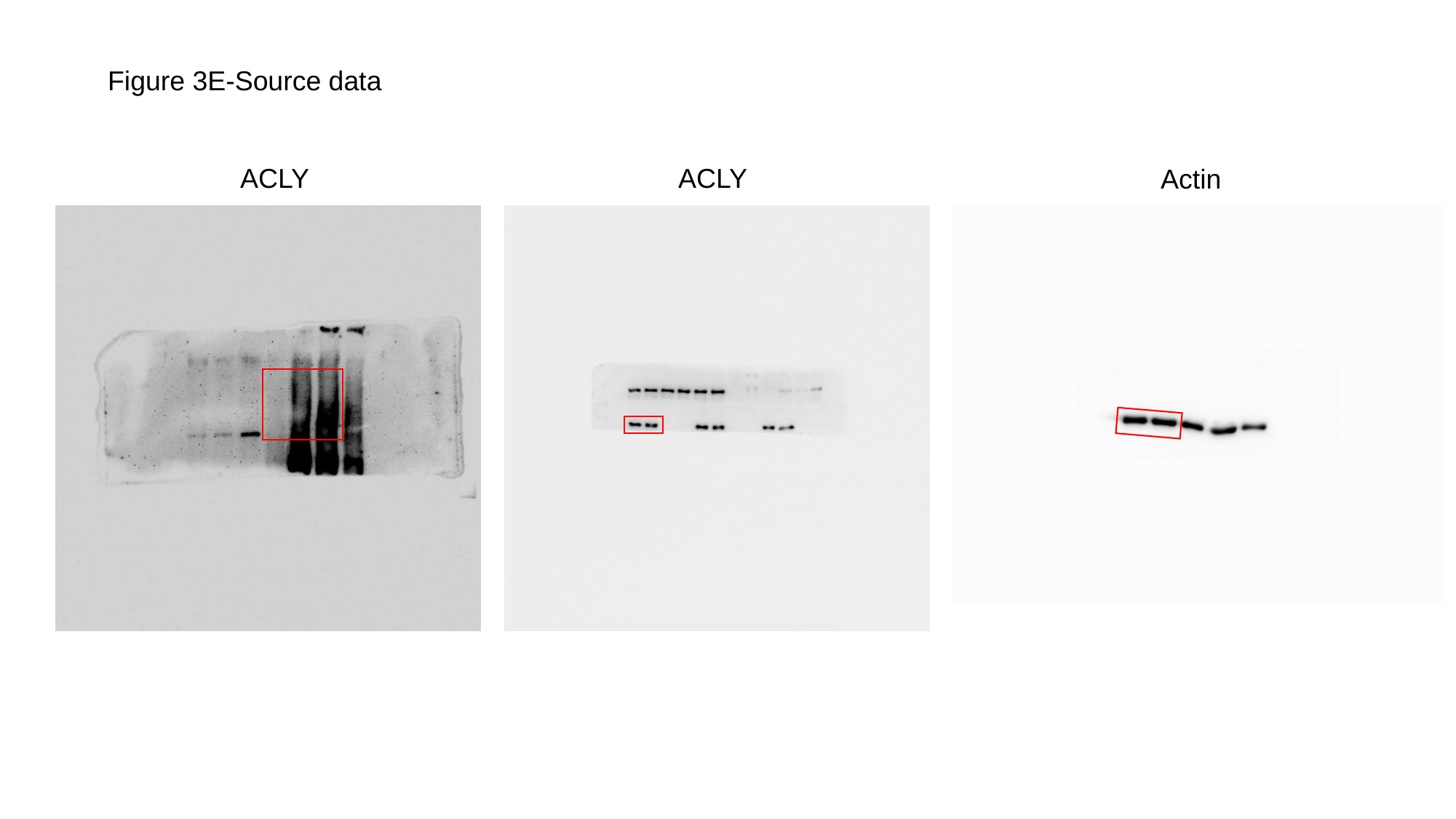

Figure 3E-Source data
ACLY
ACLY
Actin

## Slide 13
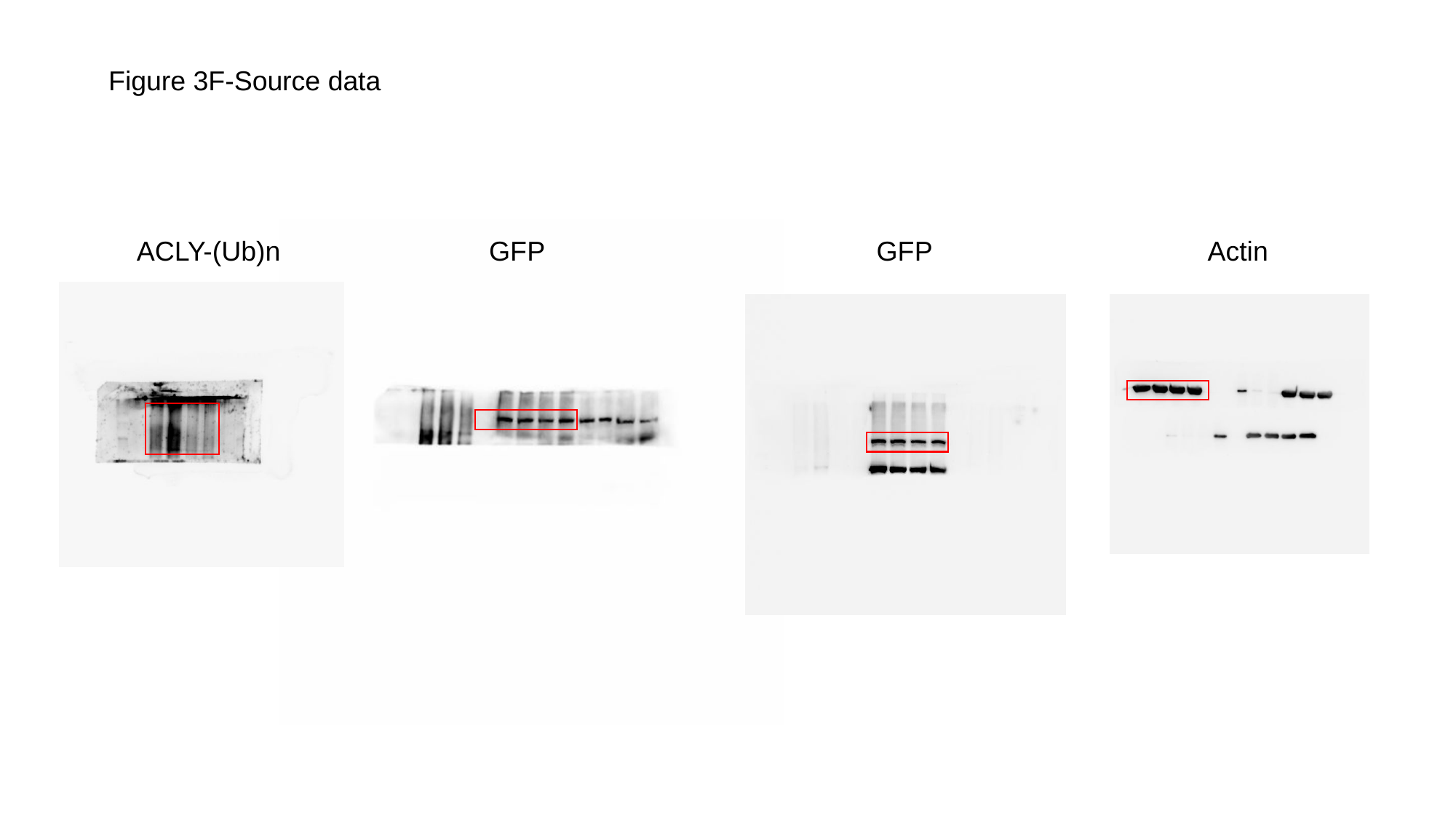

Figure 3F-Source data
ACLY-(Ub)n
GFP
Actin
GFP

## Slide 14
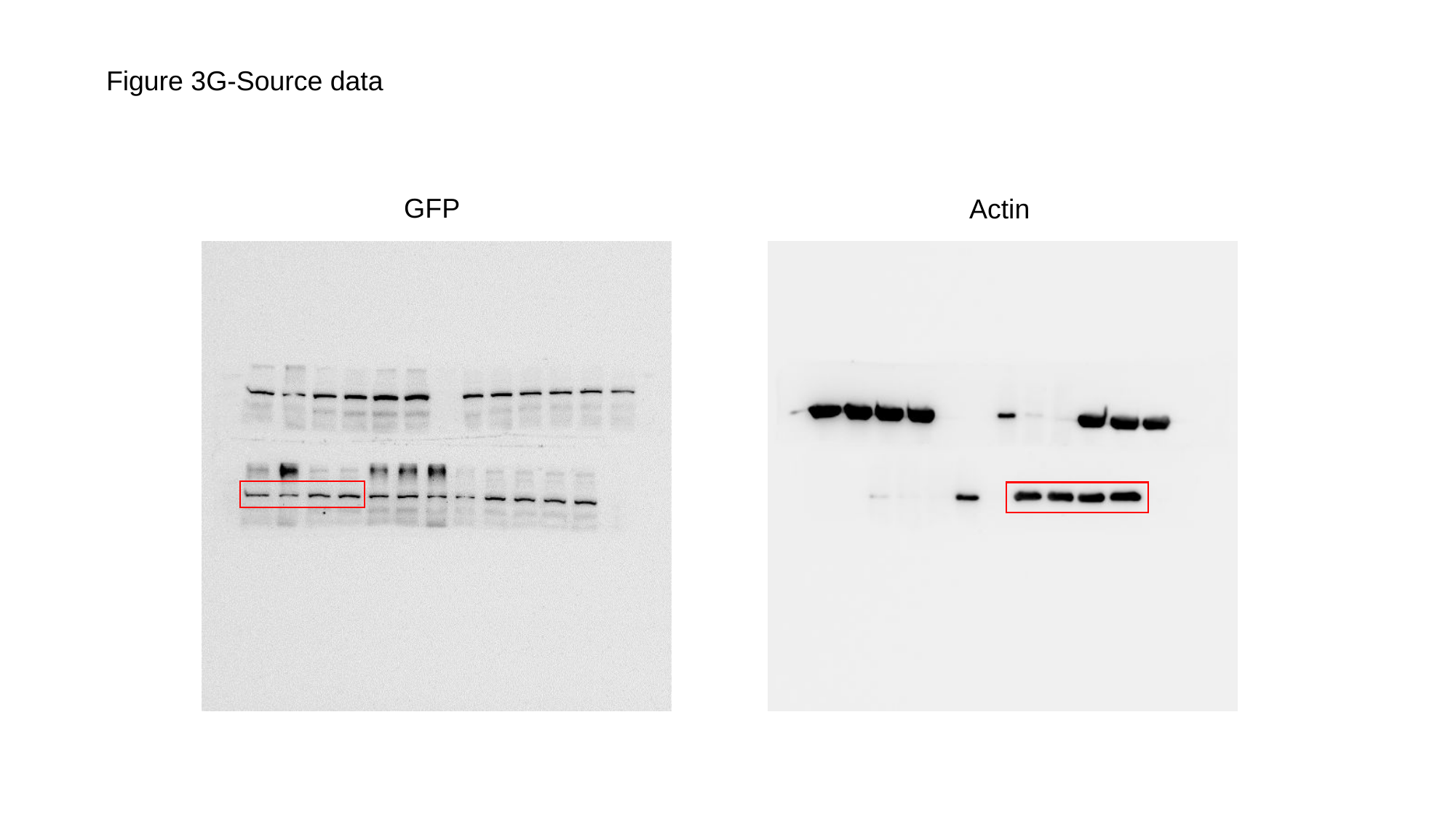

Figure 3G-Source data
GFP
Actin

## Slide 15
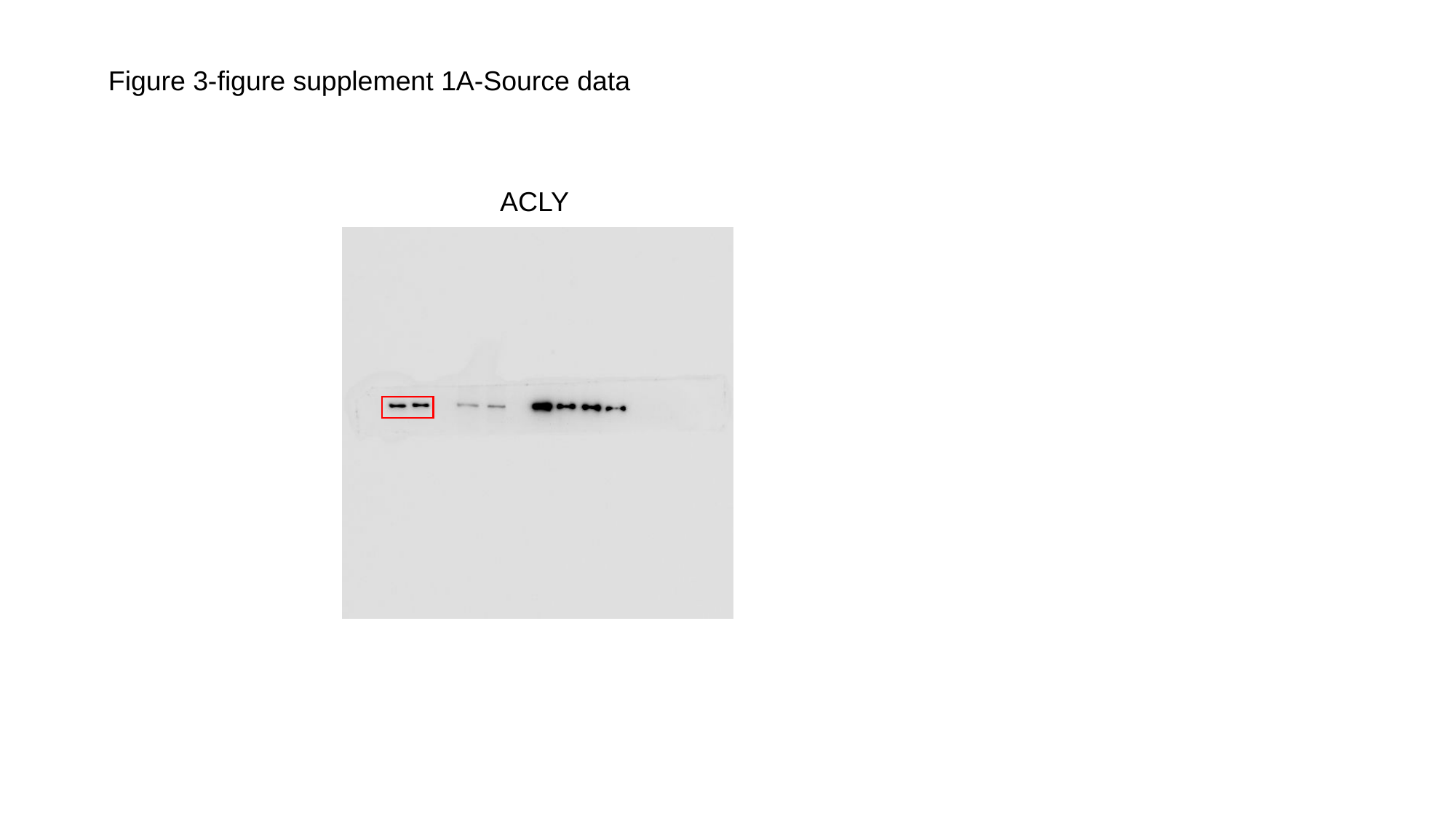

Figure 3-figure supplement 1A-Source data
ACLY

## Slide 16
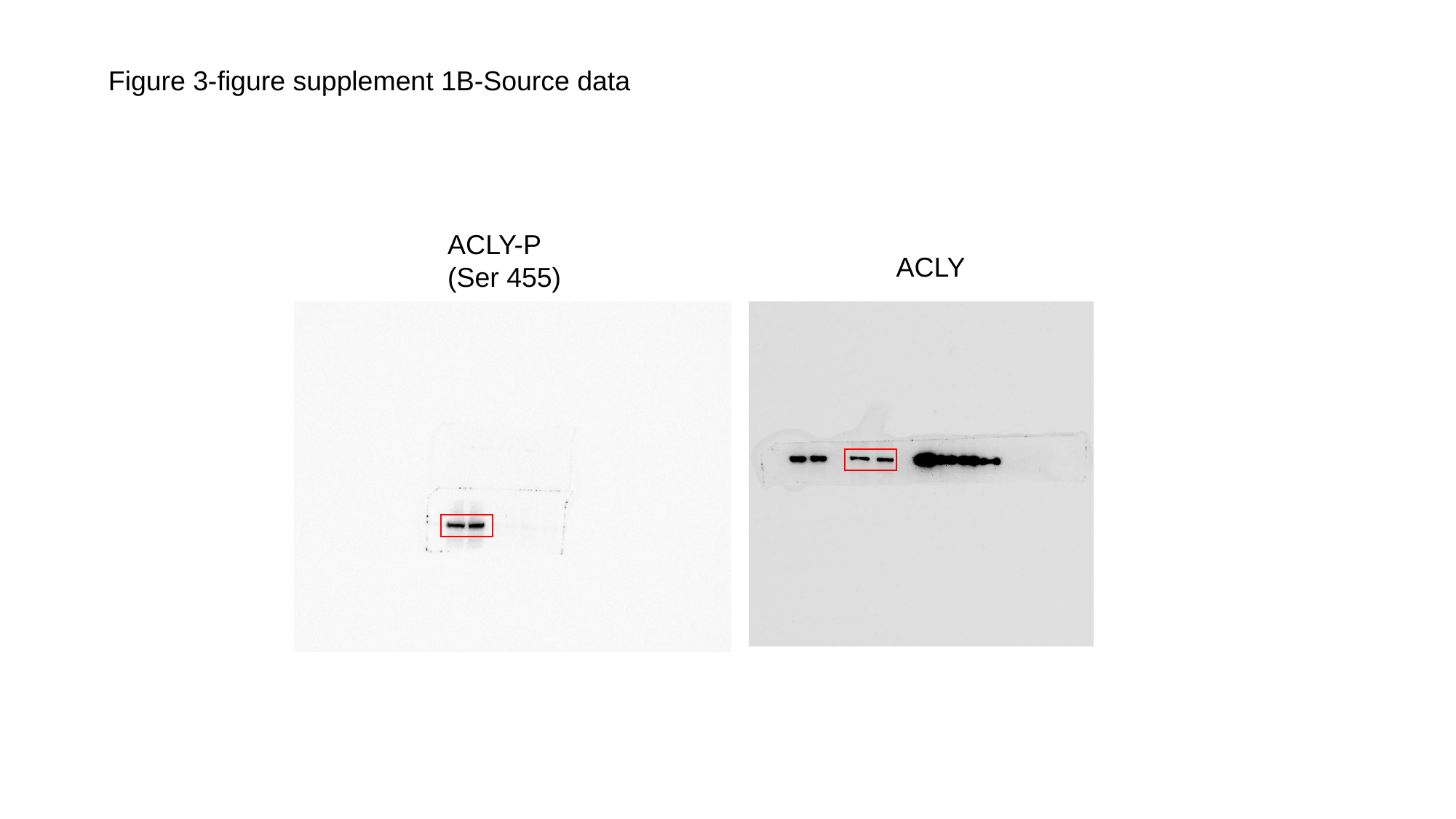

Figure 3-figure supplement 1B-Source data
ACLY-P (Ser 455)
ACLY

## Slide 17
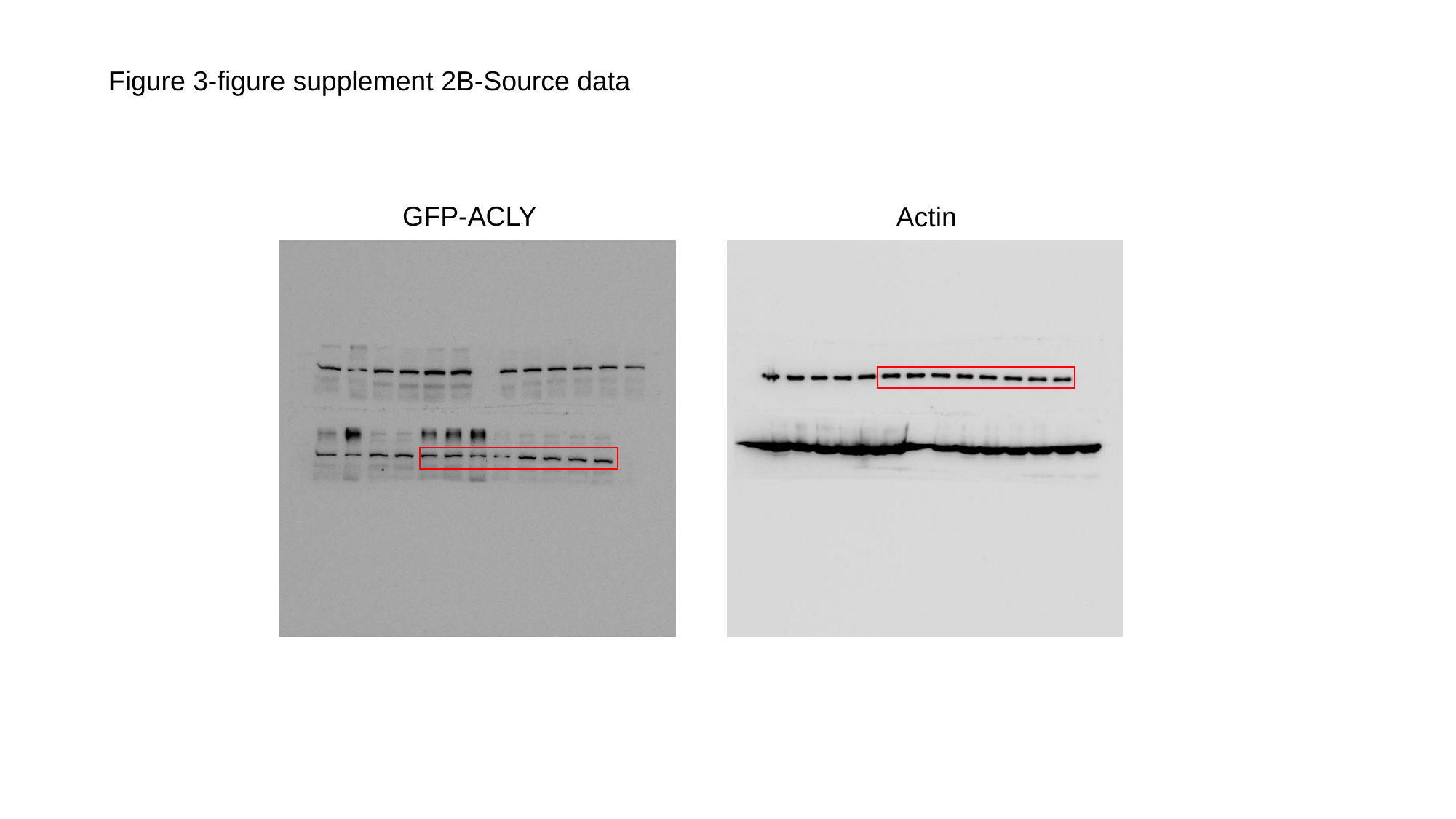

Figure 3-figure supplement 2B-Source data
GFP-ACLY
Actin

## Slide 18
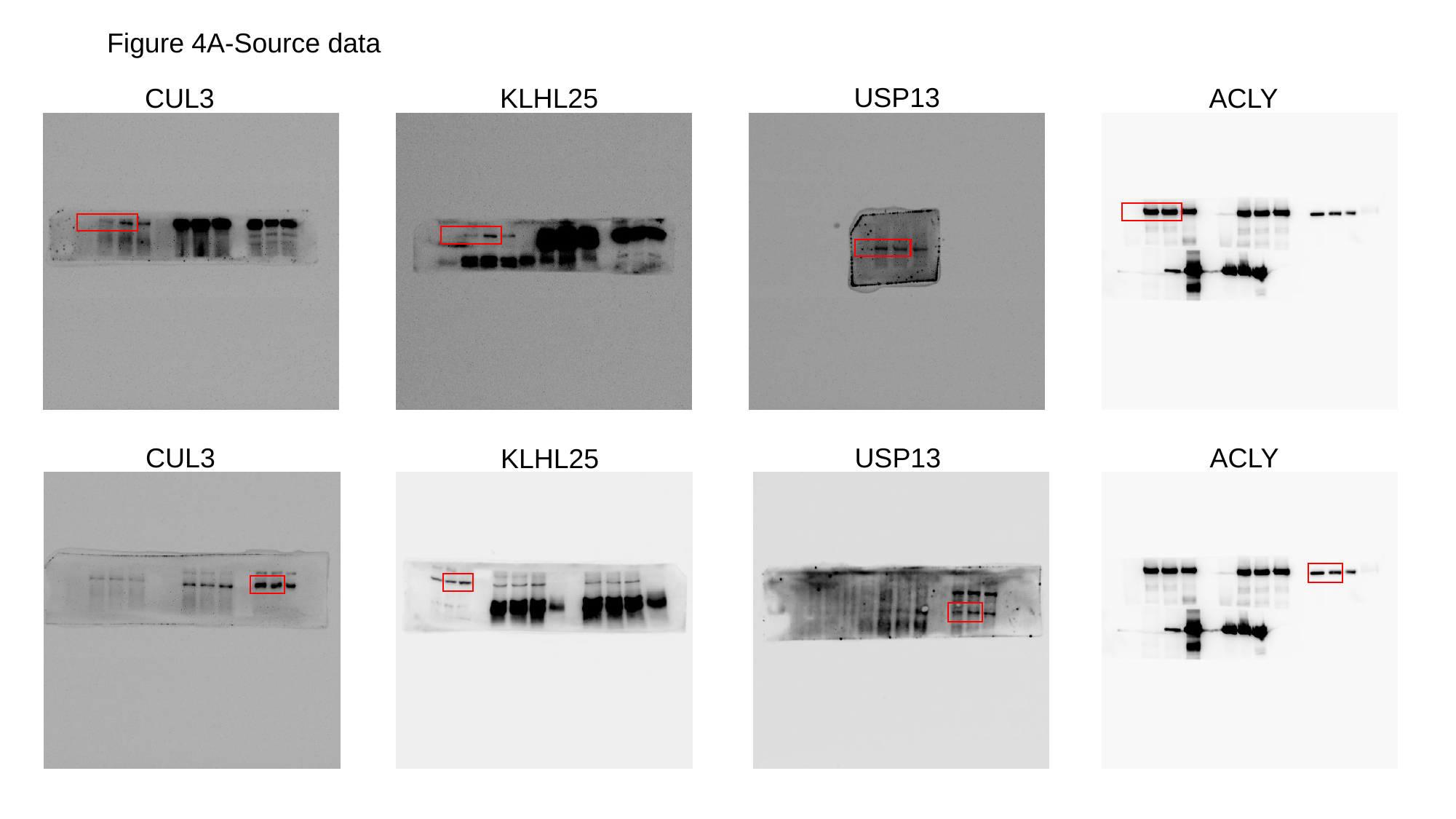

Figure 4A-Source data
USP13
CUL3
ACLY
KLHL25
USP13
CUL3
ACLY
KLHL25

## Slide 19
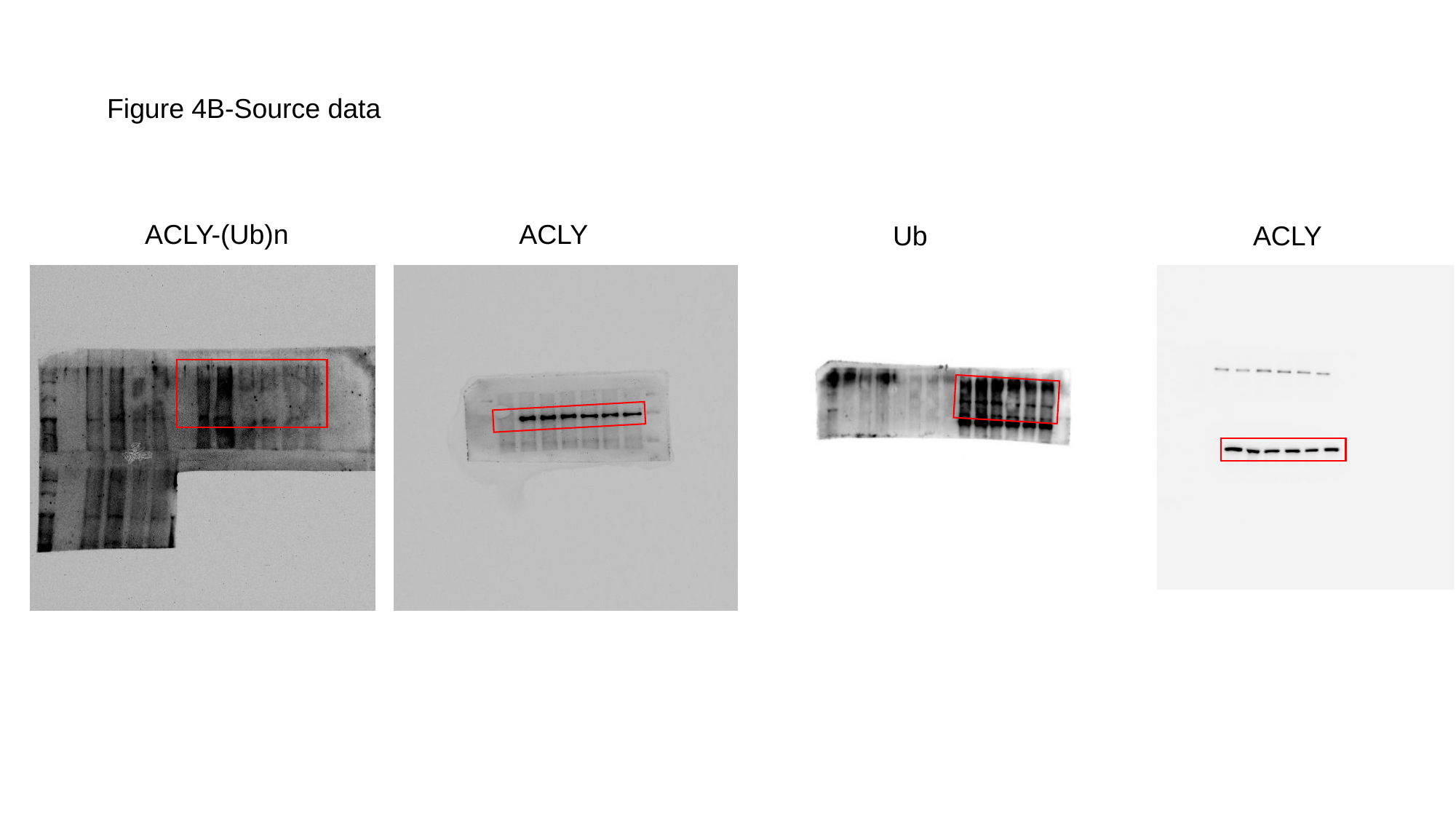

Figure 4B-Source data
ACLY-(Ub)n
ACLY
Ub
ACLY

## Slide 20
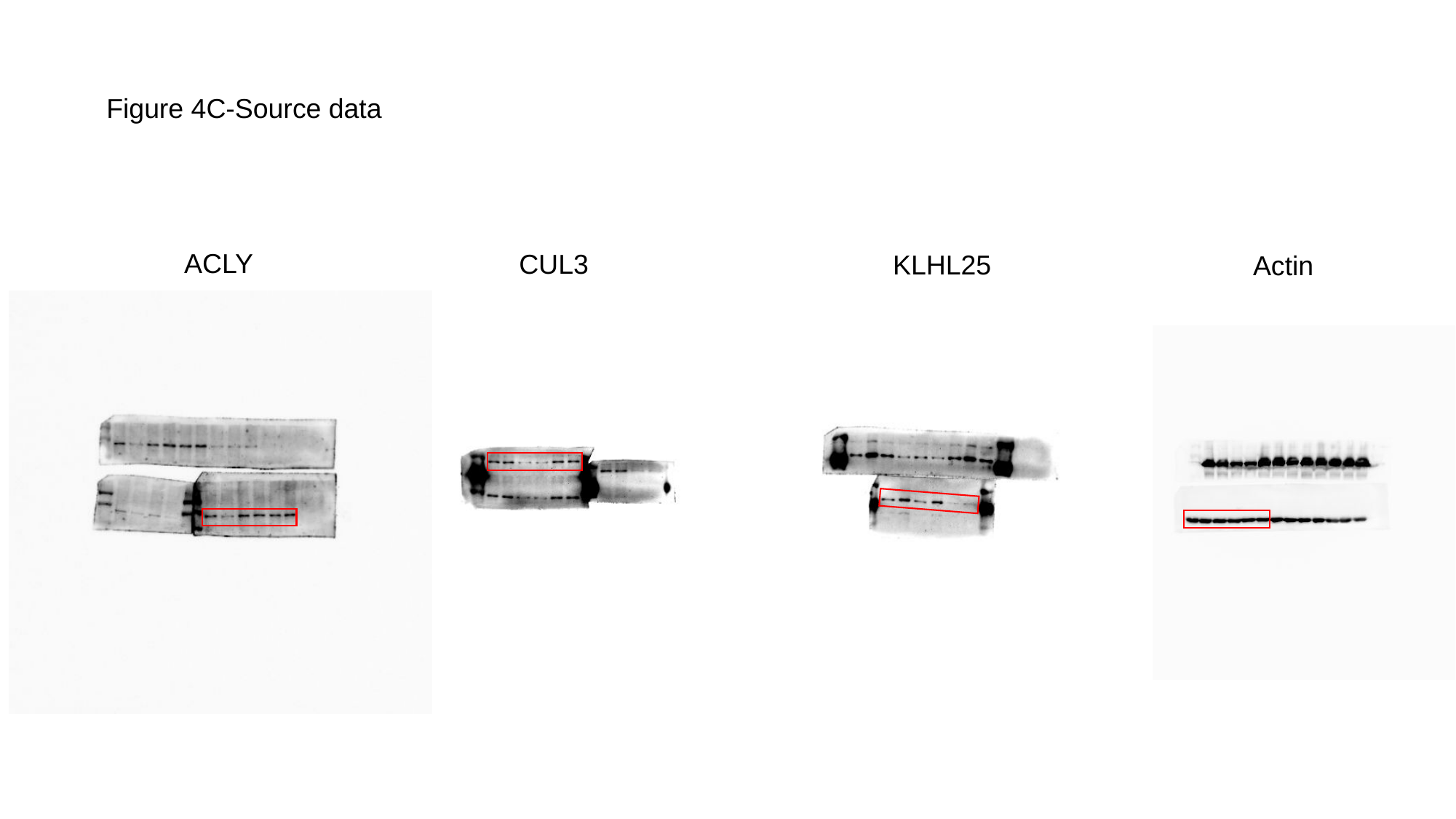

Figure 4C-Source data
ACLY
CUL3
KLHL25
Actin

## Slide 21
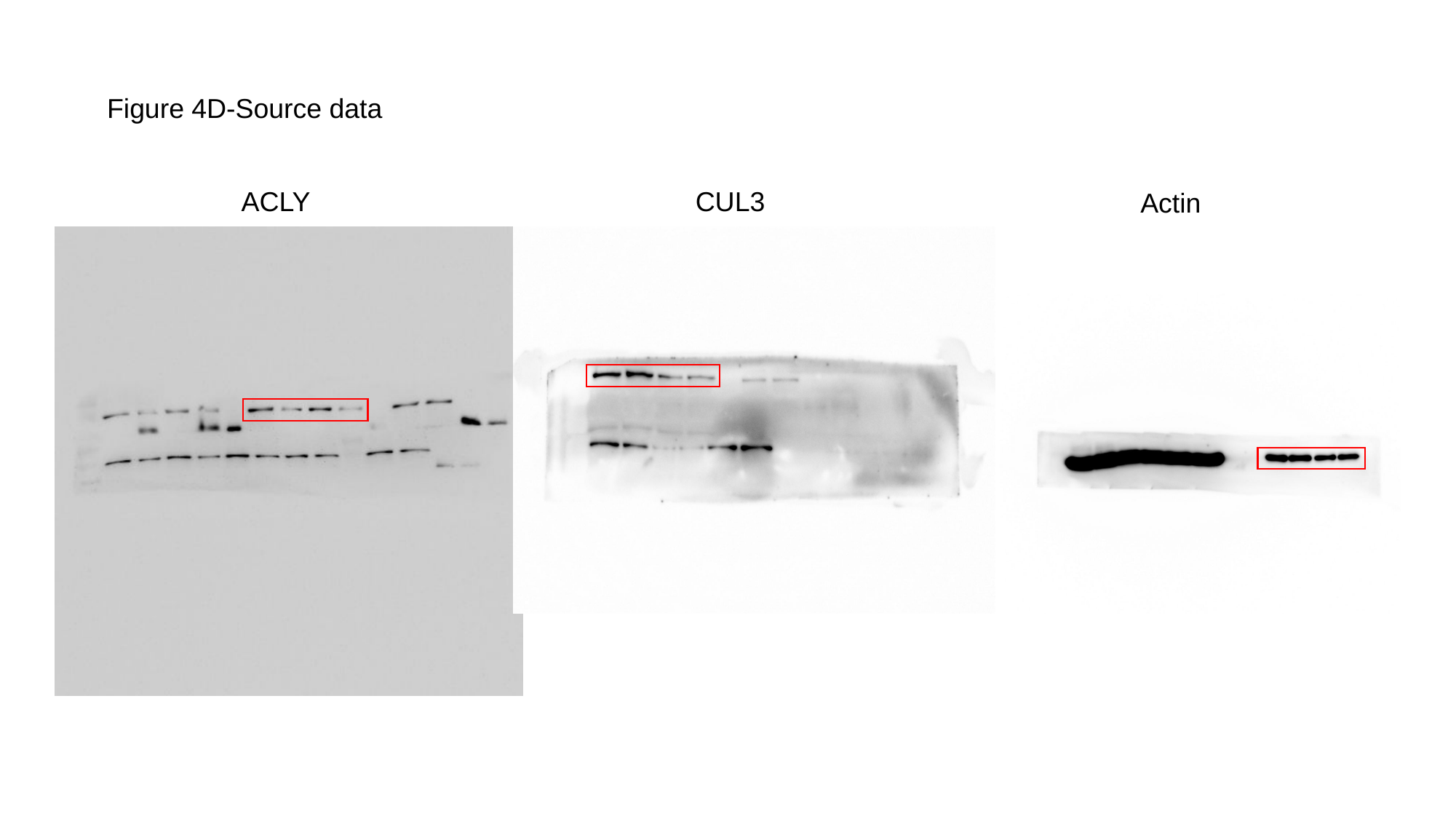

Figure 4D-Source data
ACLY
CUL3
Actin

## Slide 22
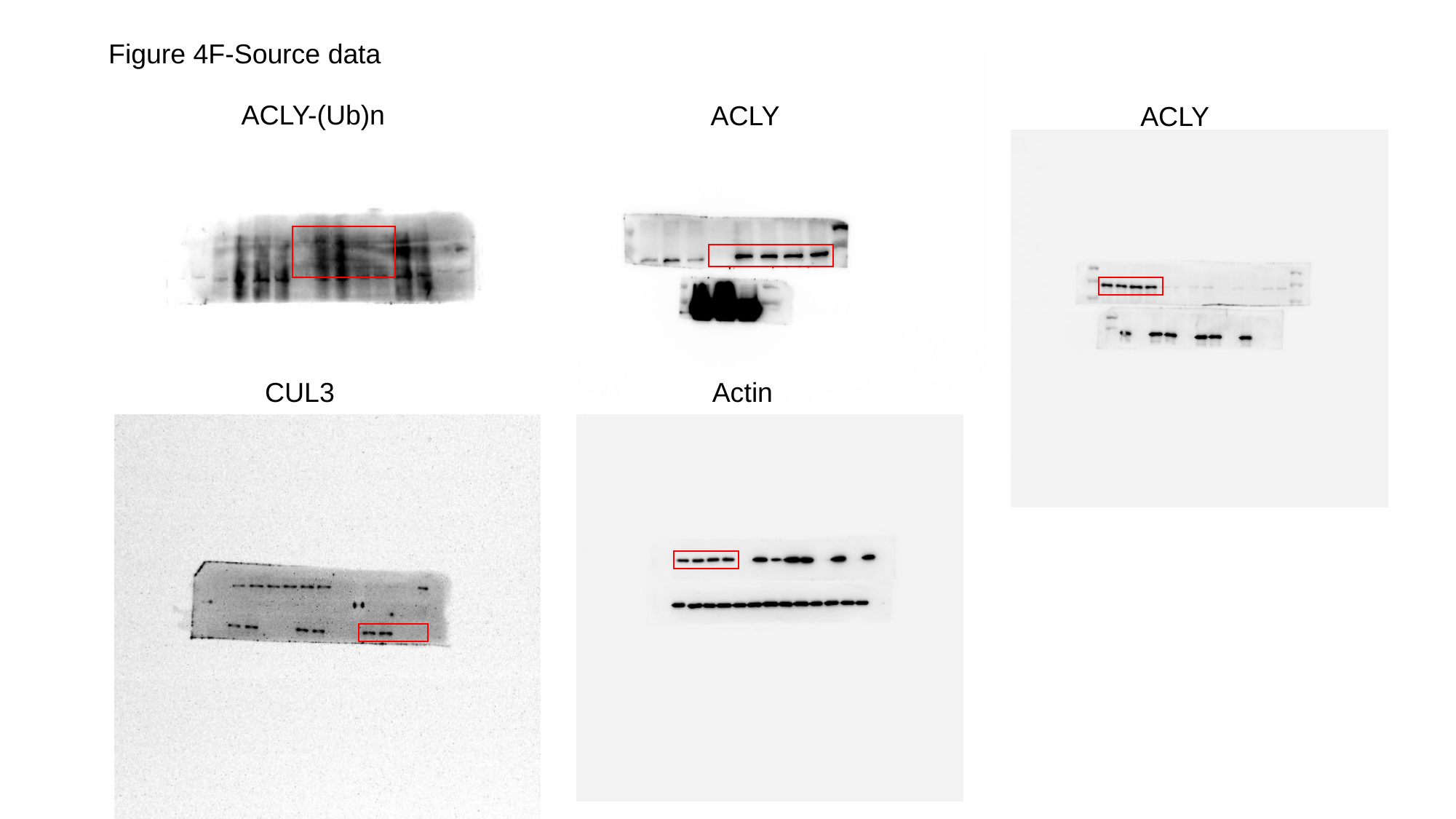

Figure 4F-Source data
ACLY-(Ub)n
ACLY
ACLY
CUL3
Actin

## Slide 23
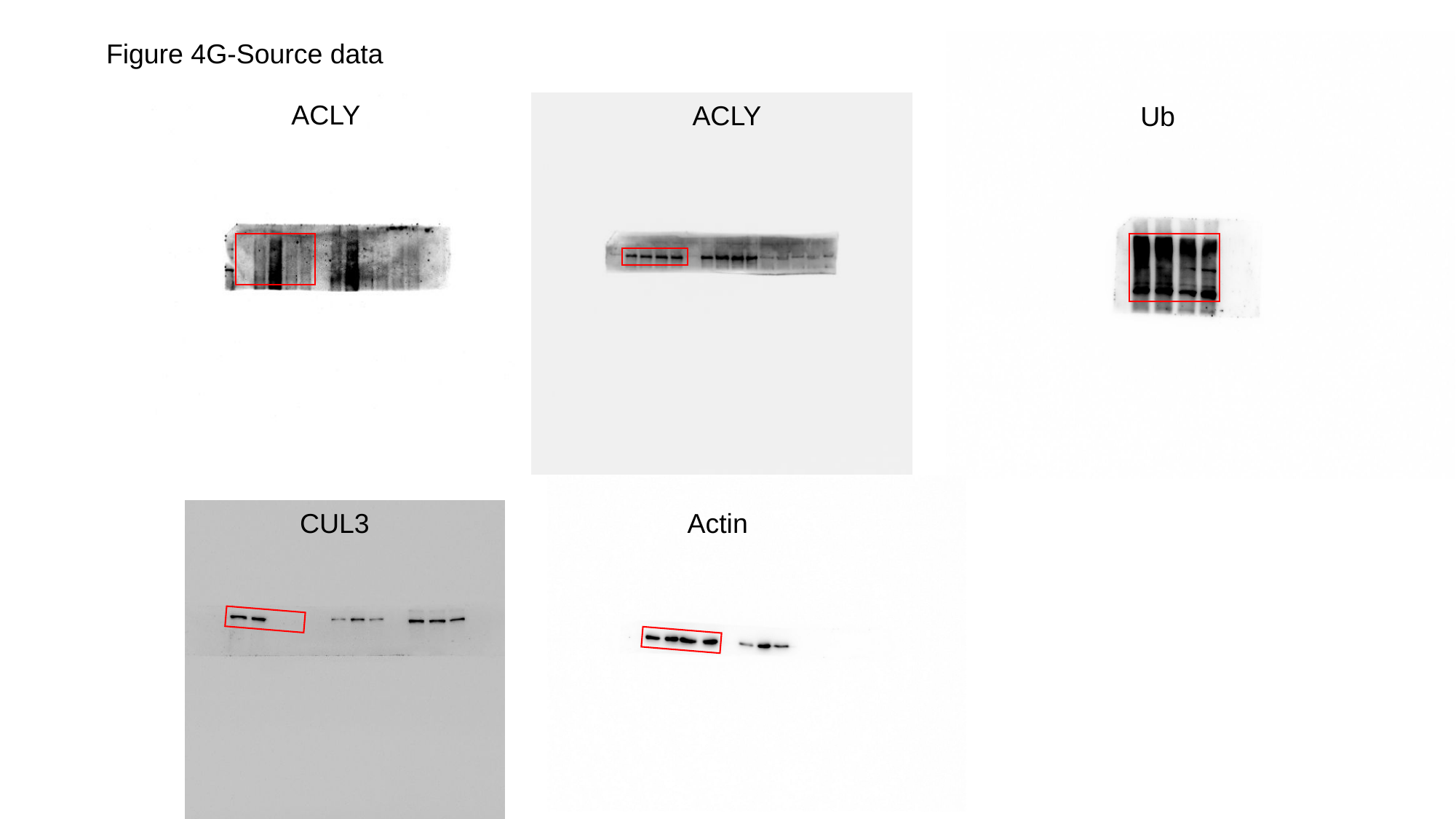

Figure 4G-Source data
ACLY
ACLY
Ub
CUL3
Actin

## Slide 24
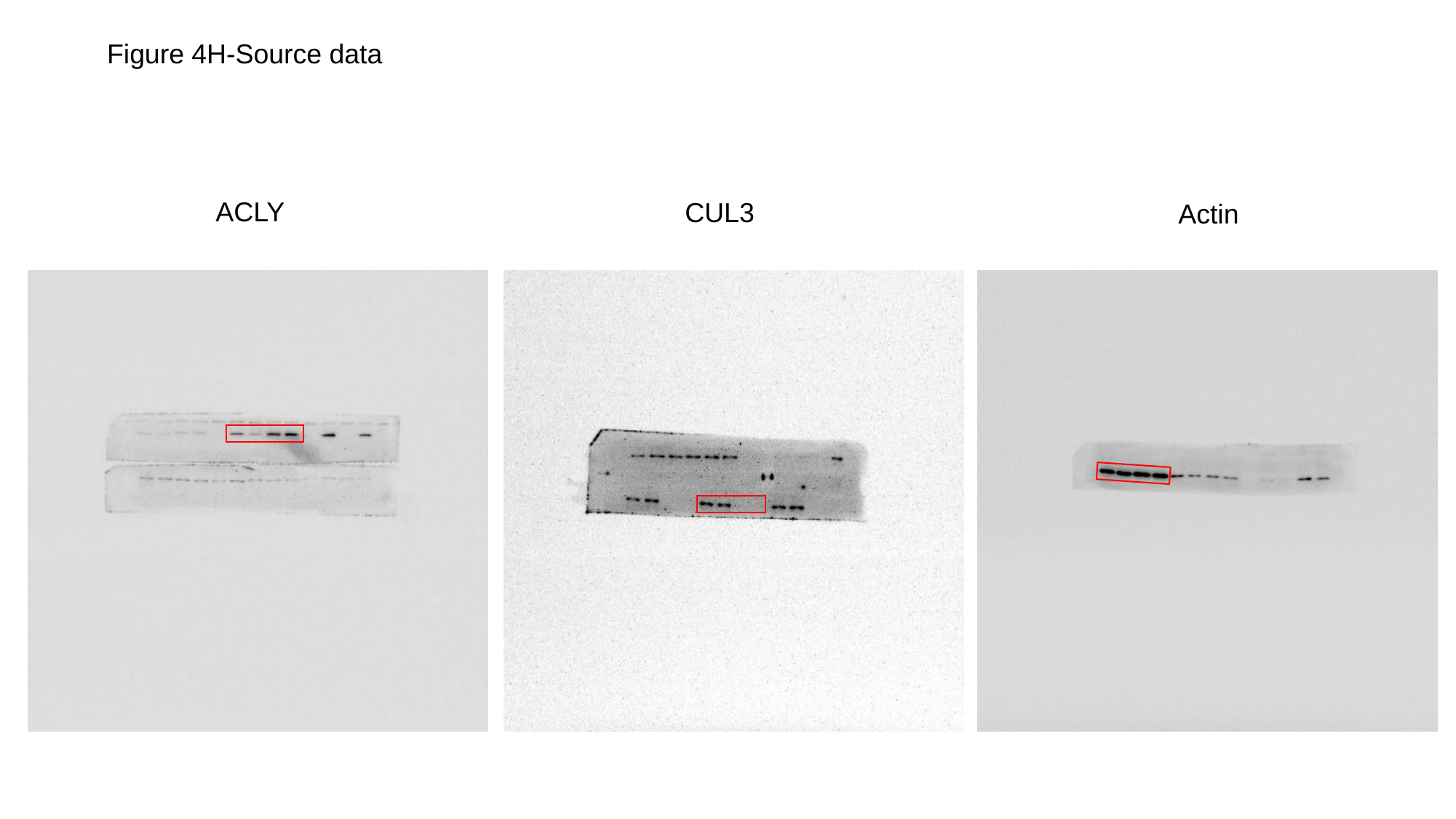

Figure 4H-Source data
ACLY
CUL3
Actin

## Slide 25
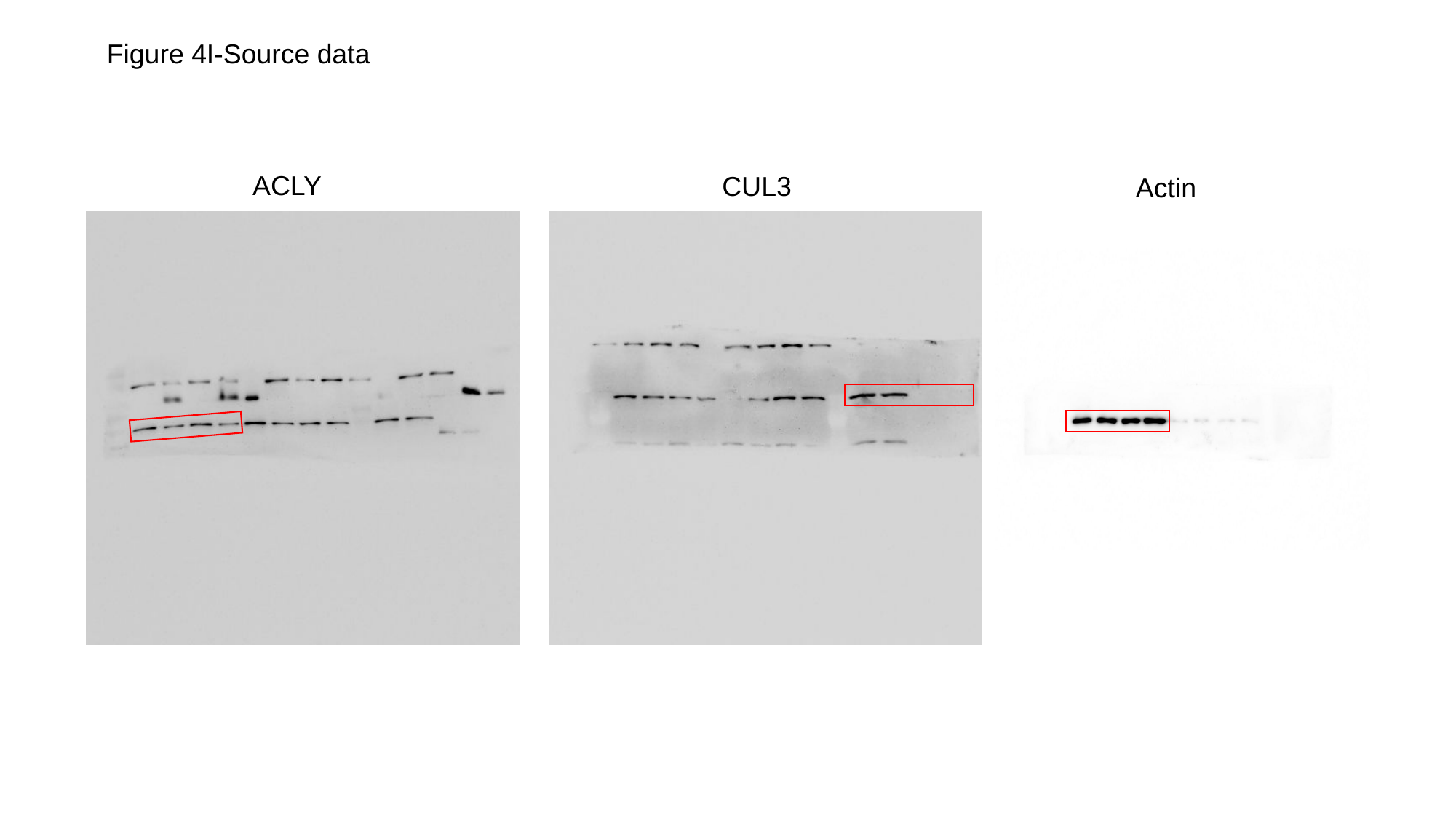

Figure 4I-Source data
ACLY
CUL3
Actin

## Slide 26
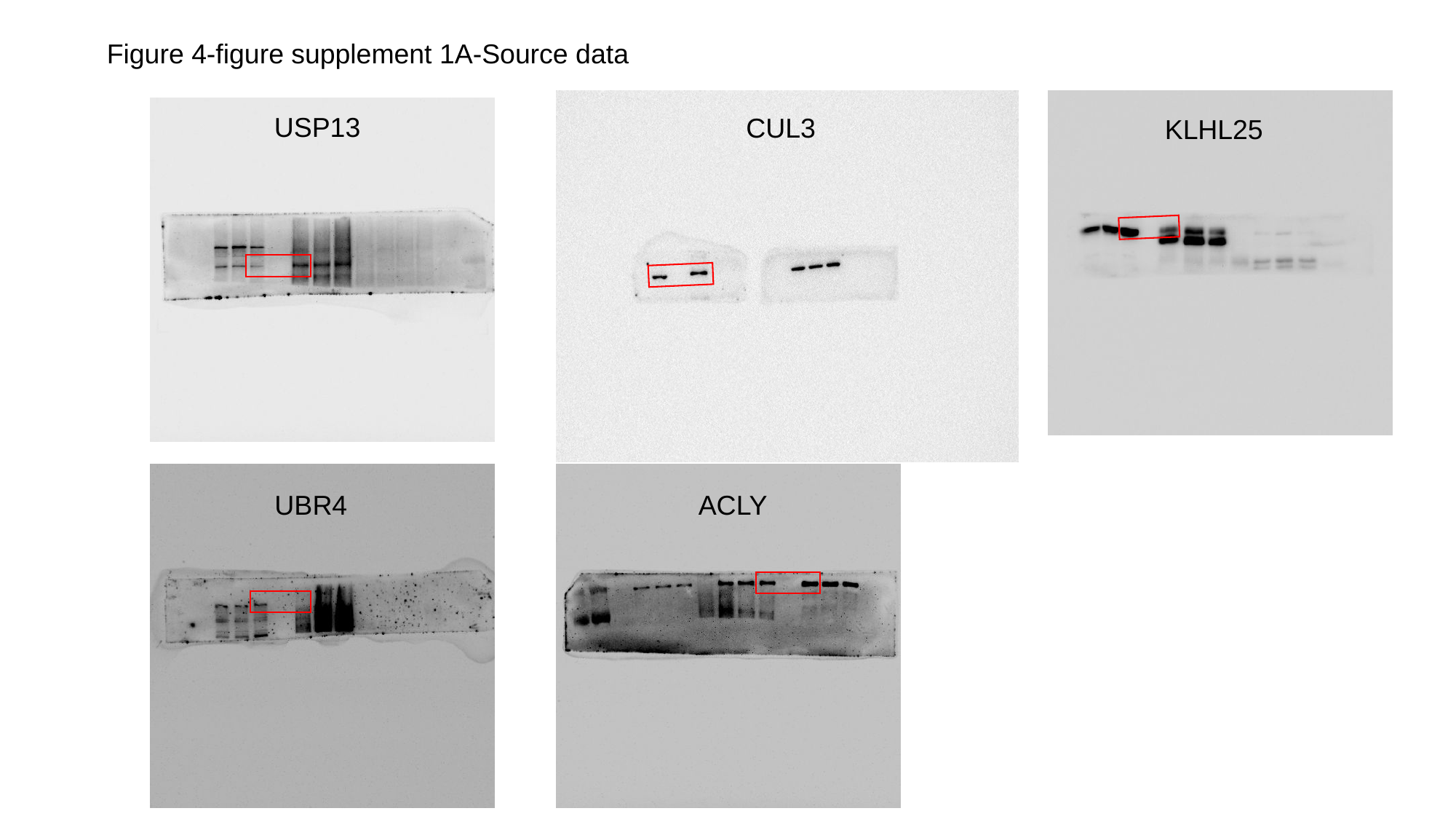

Figure 4-figure supplement 1A-Source data
USP13
CUL3
KLHL25
UBR4
ACLY

## Slide 27
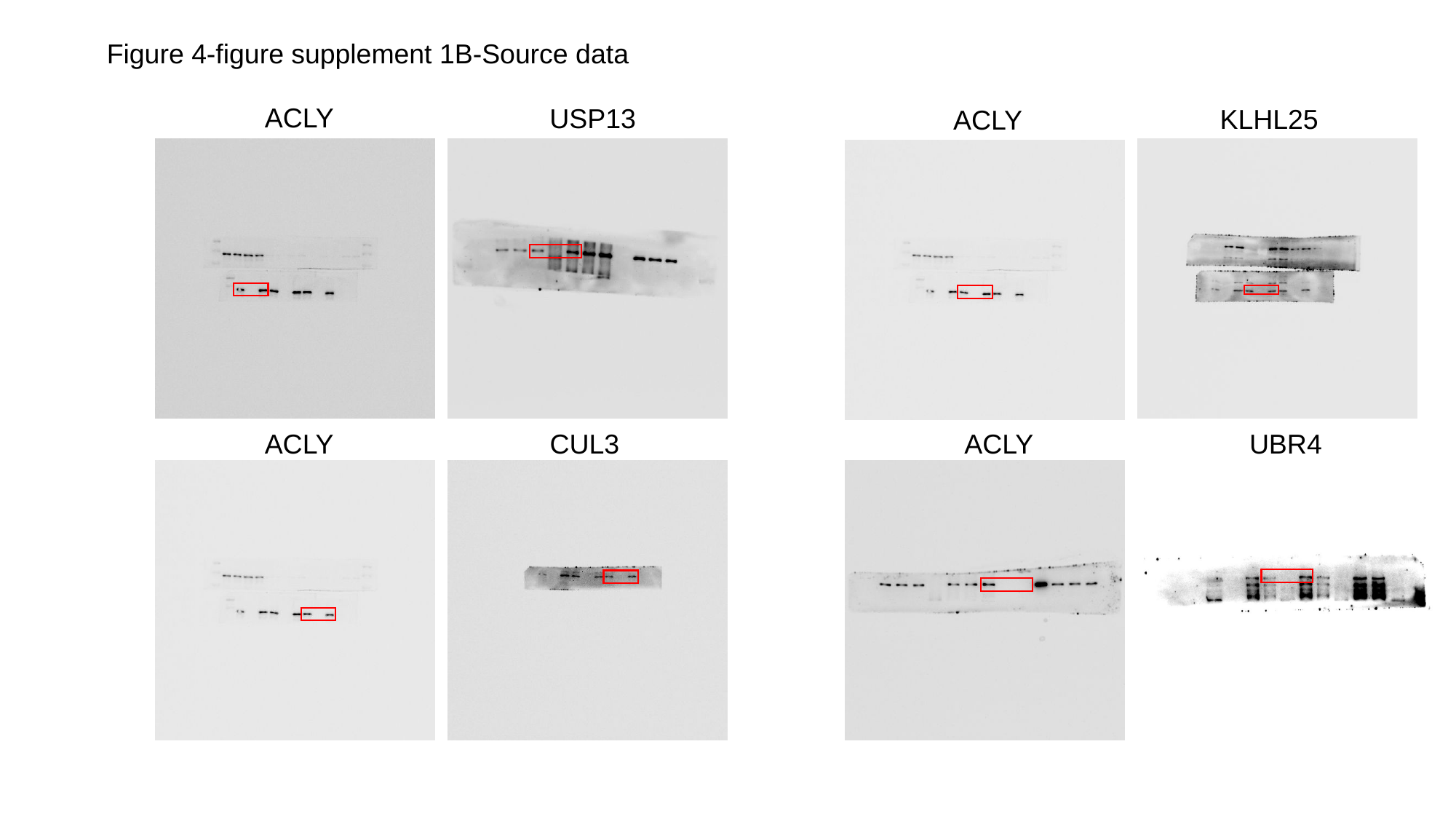

Figure 4-figure supplement 1B-Source data
ACLY
USP13
KLHL25
ACLY
ACLY
ACLY
CUL3
UBR4

## Slide 28
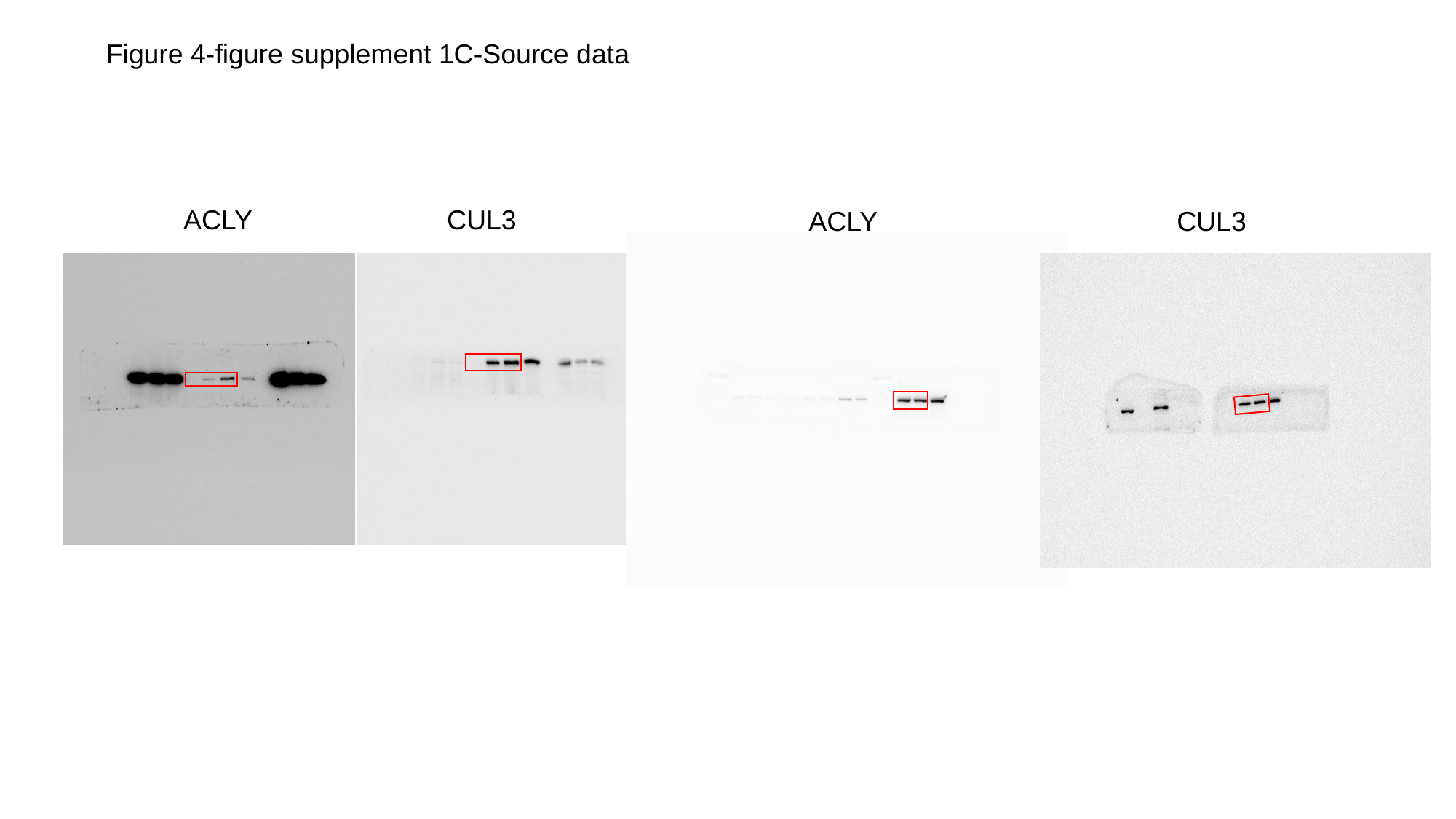

Figure 4-figure supplement 1C-Source data
ACLY
CUL3
CUL3
ACLY

## Slide 29
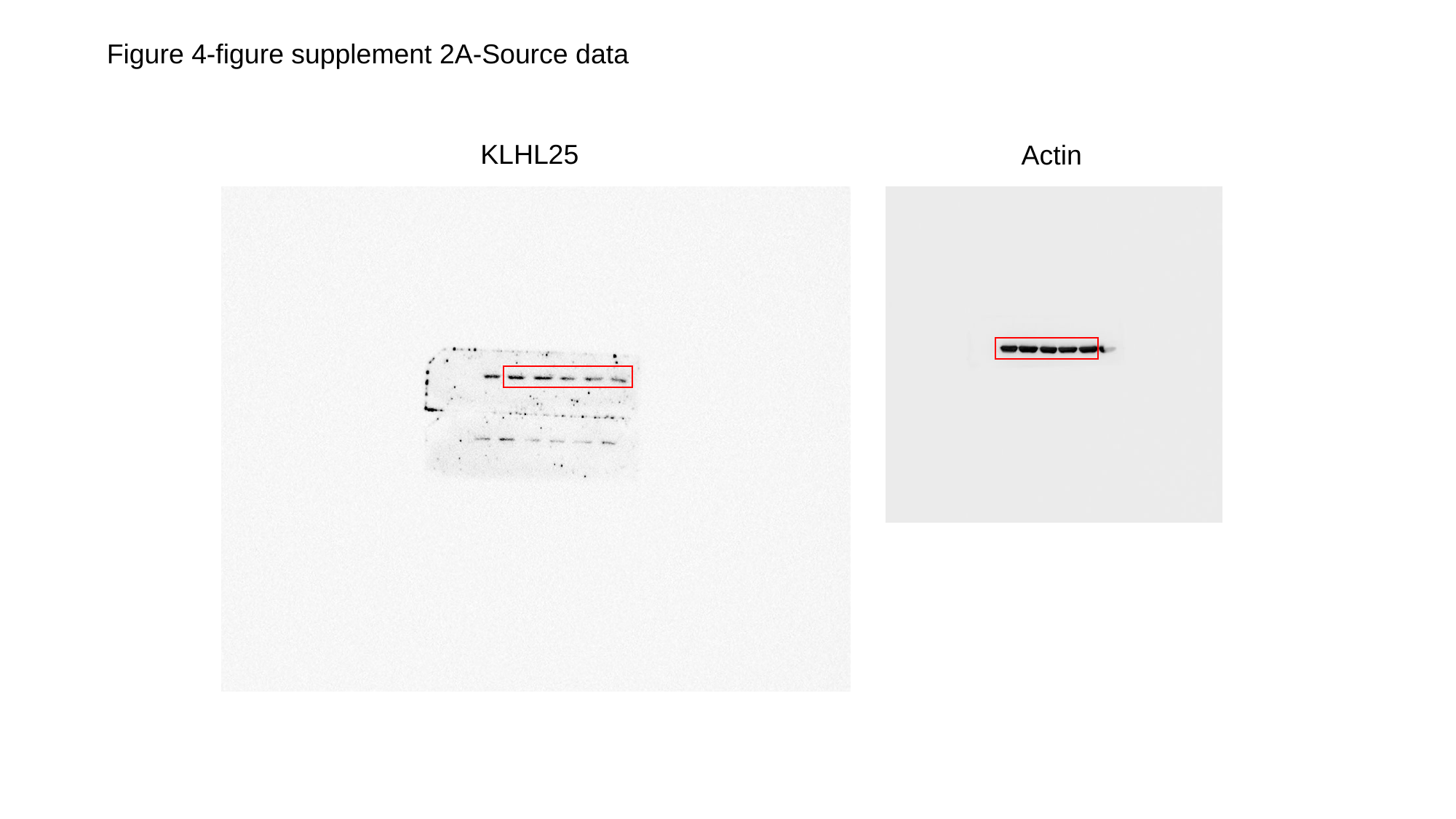

Figure 4-figure supplement 2A-Source data
KLHL25
Actin

## Slide 30
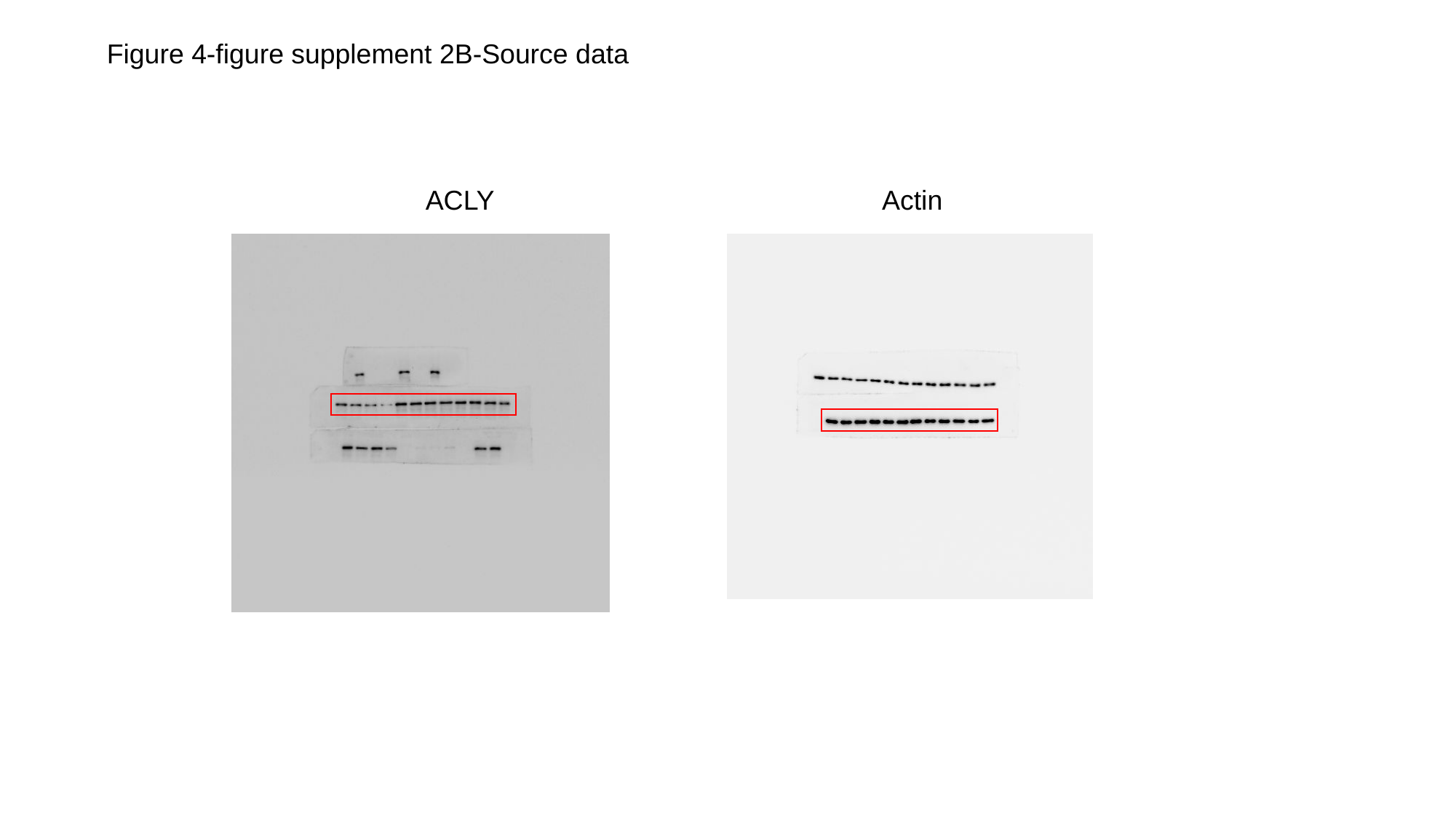

Figure 4-figure supplement 2B-Source data
ACLY
Actin

## Slide 31
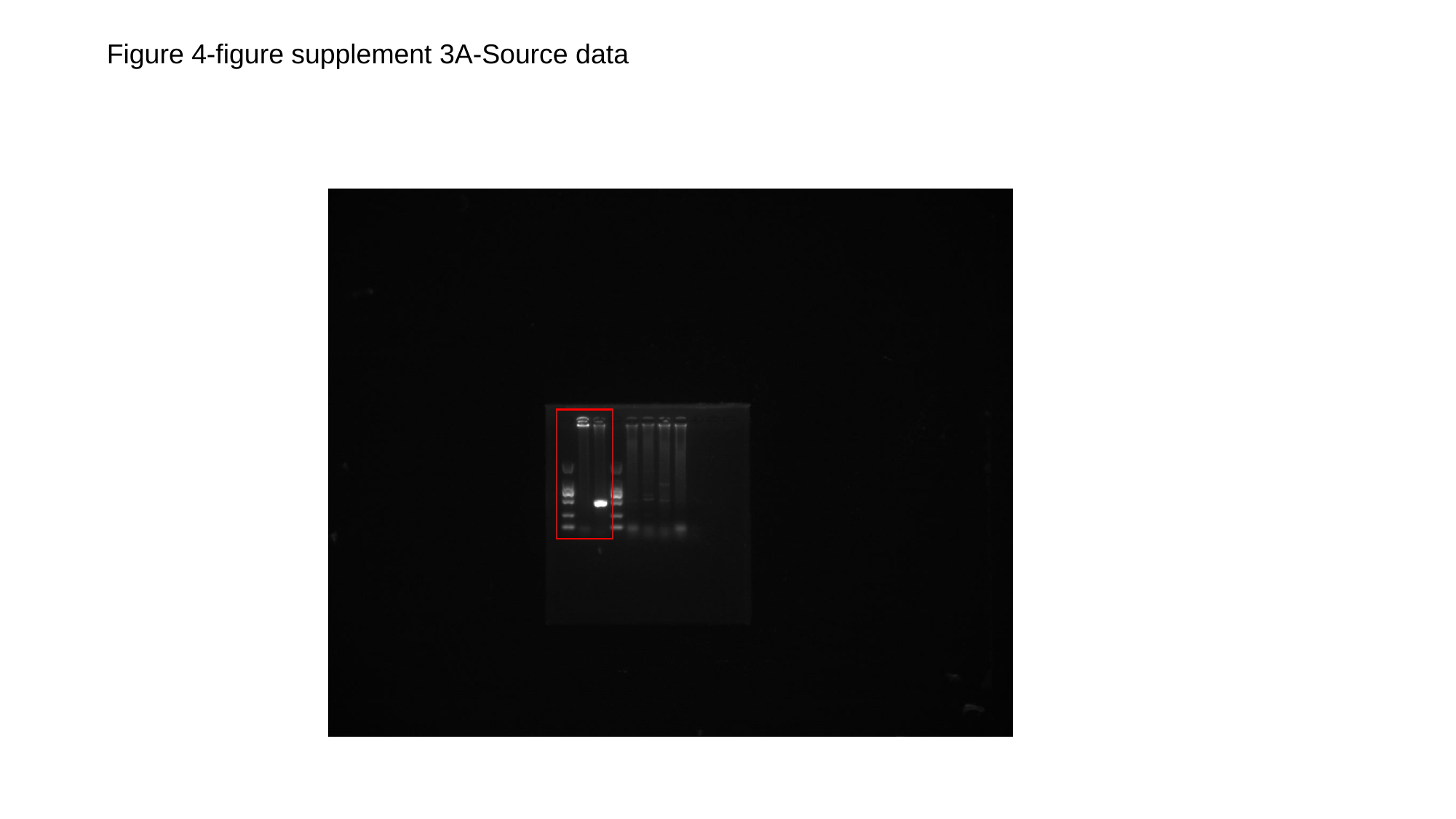

Figure 4-figure supplement 3A-Source data

## Slide 32
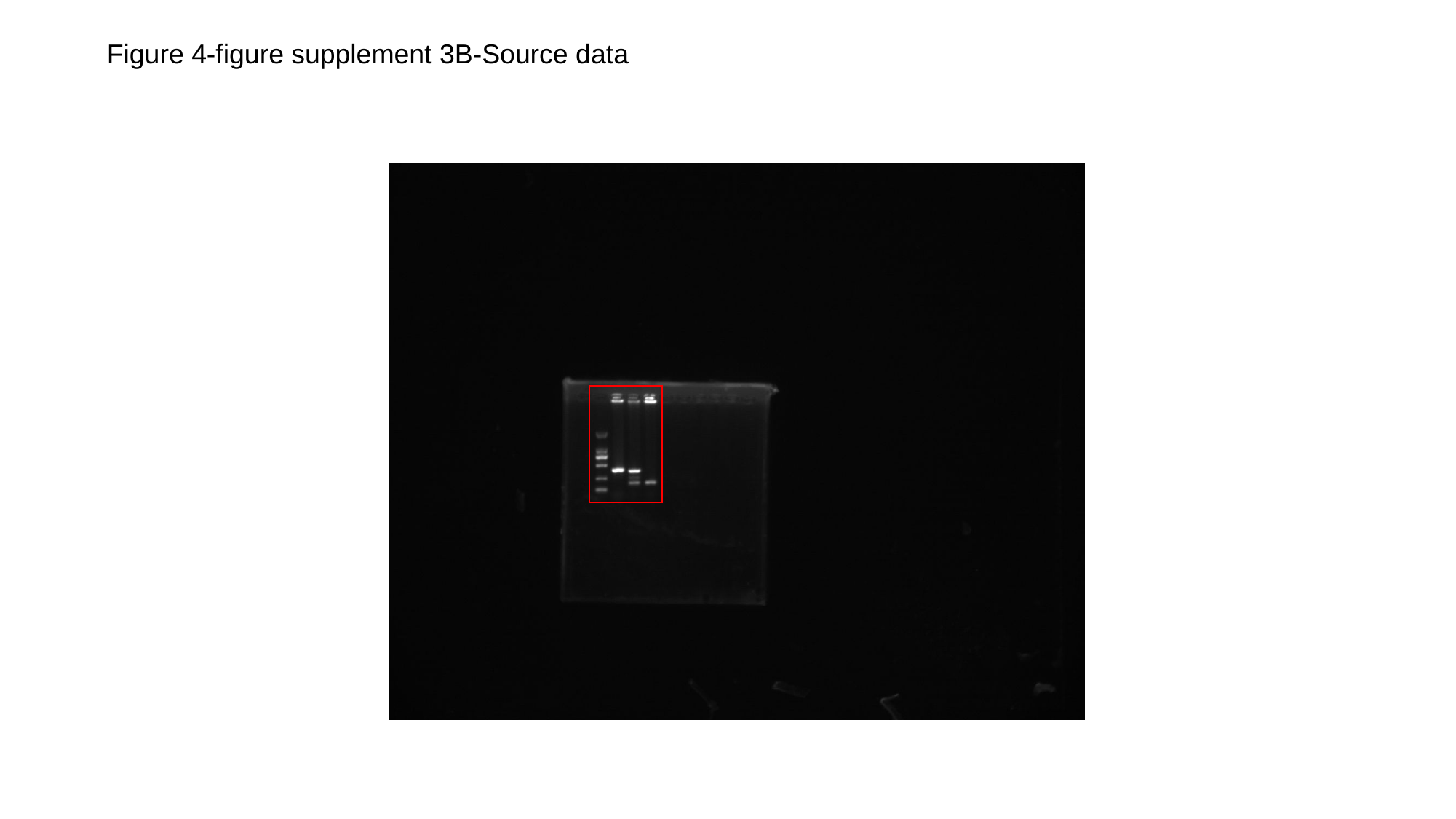

Figure 4-figure supplement 3B-Source data

## Slide 33
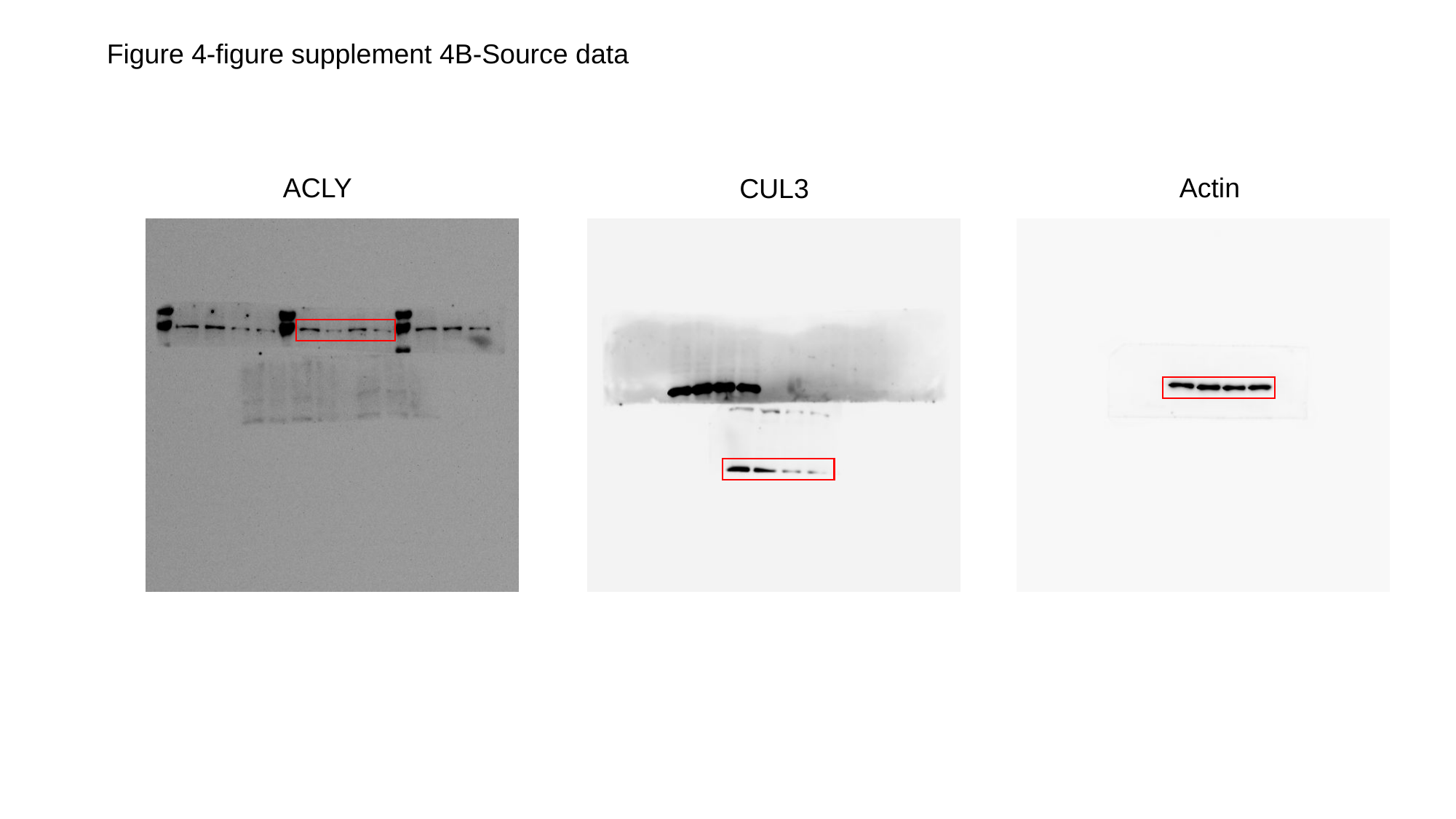

Figure 4-figure supplement 4B-Source data
ACLY
Actin
CUL3

## Slide 34
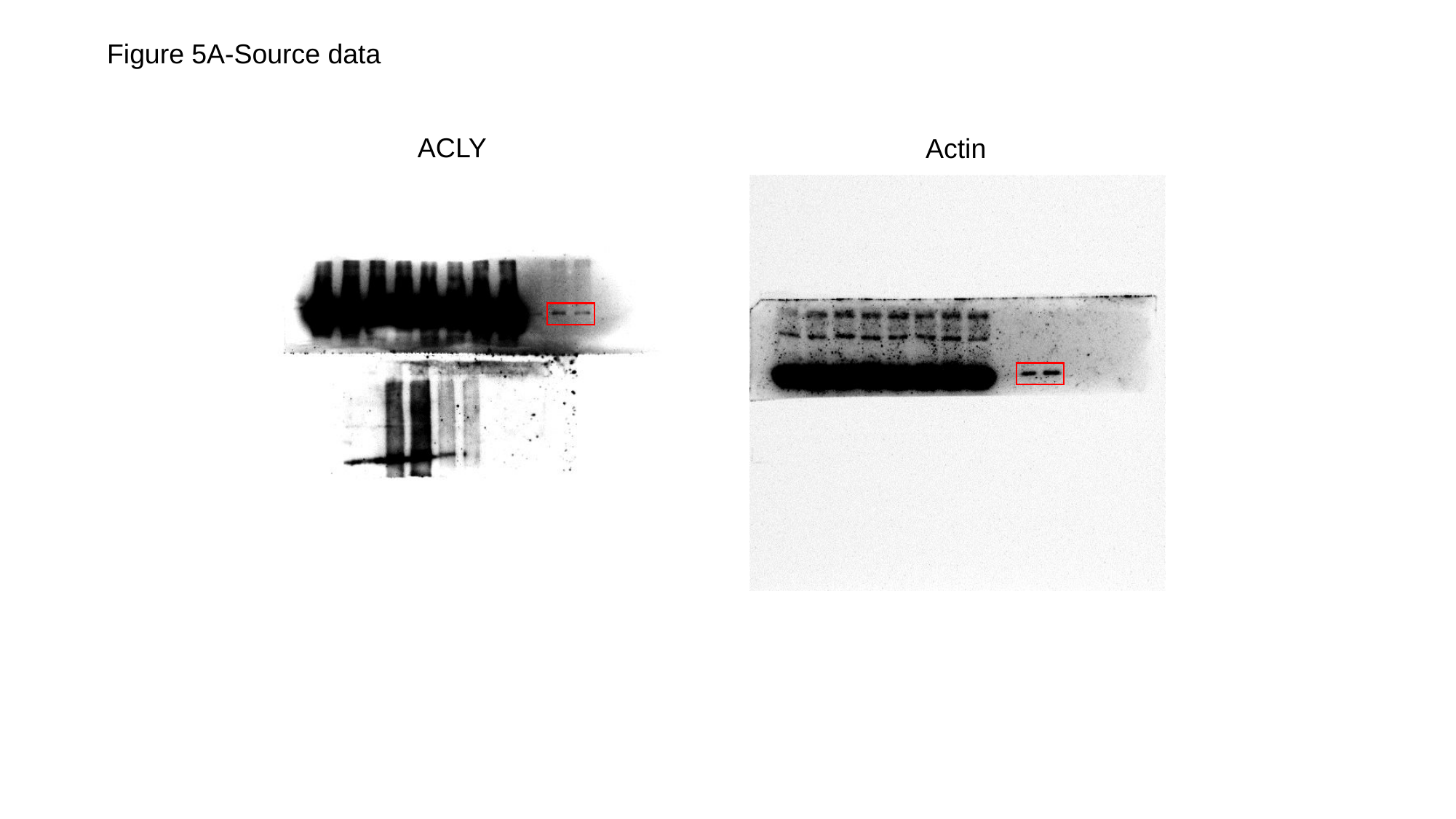

Figure 5A-Source data
ACLY
Actin

## Slide 35
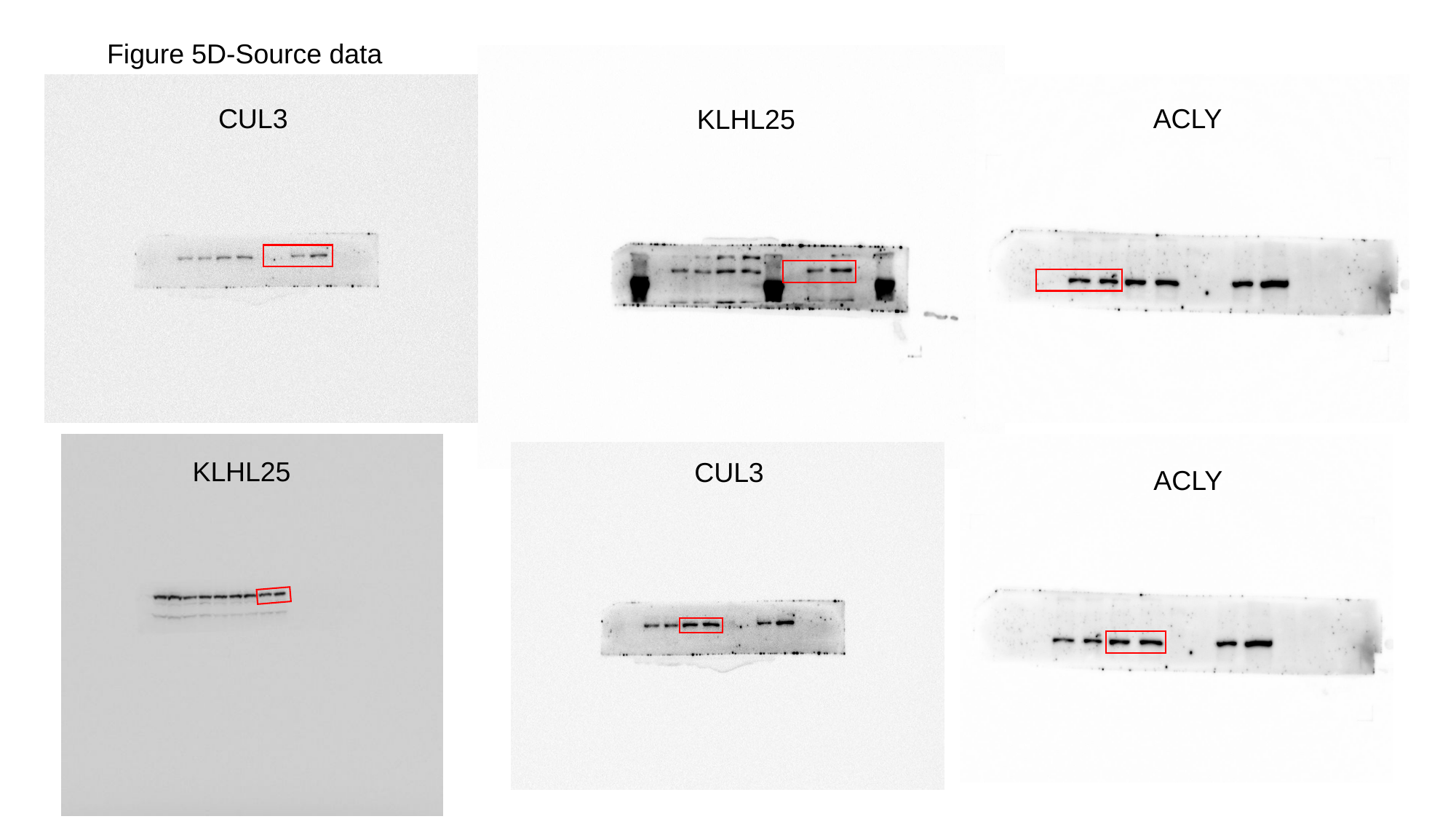

Figure 5D-Source data
ACLY
CUL3
KLHL25
KLHL25
CUL3
ACLY

## Slide 36
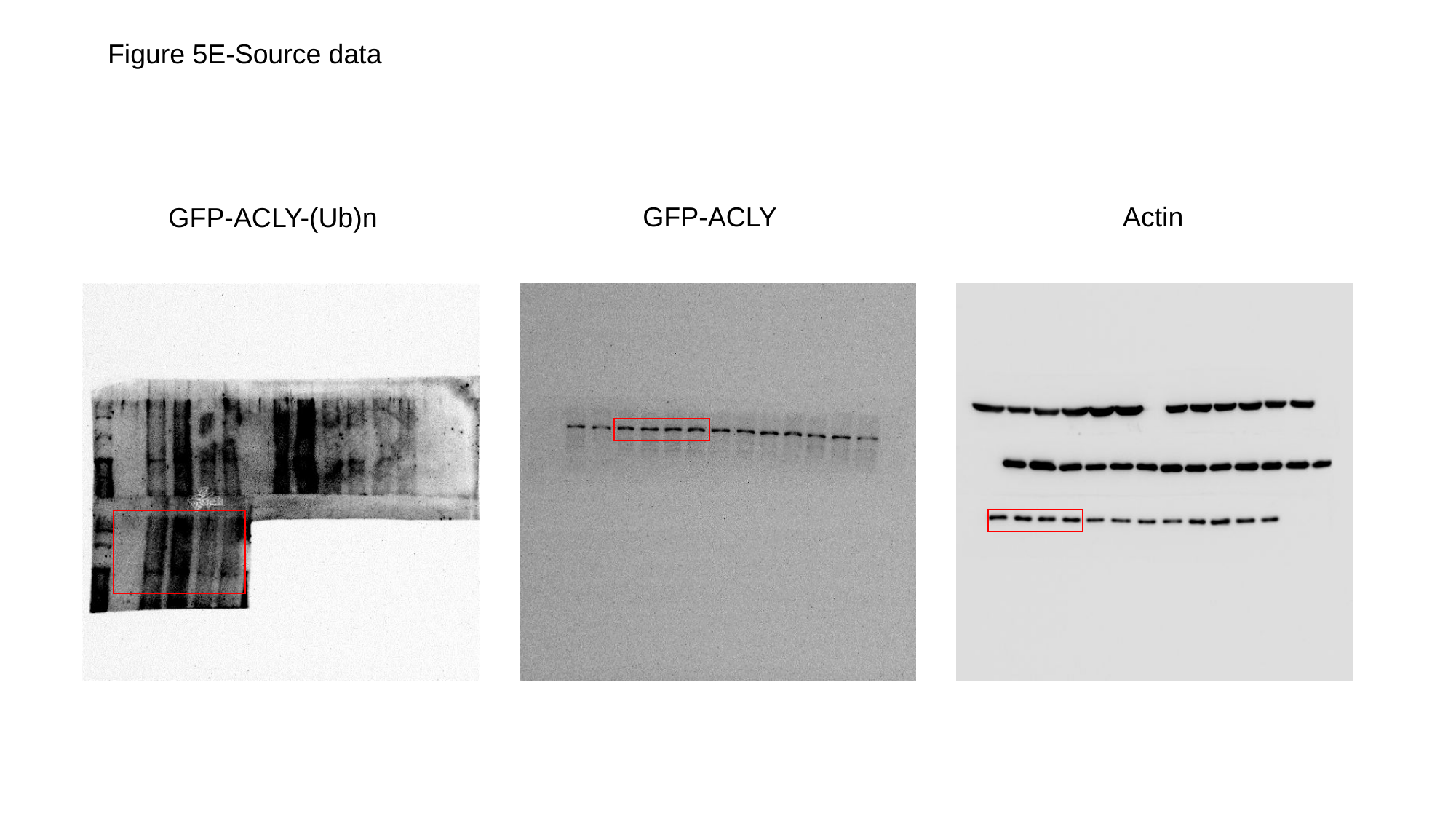

Figure 5E-Source data
GFP-ACLY
Actin
GFP-ACLY-(Ub)n

## Slide 37
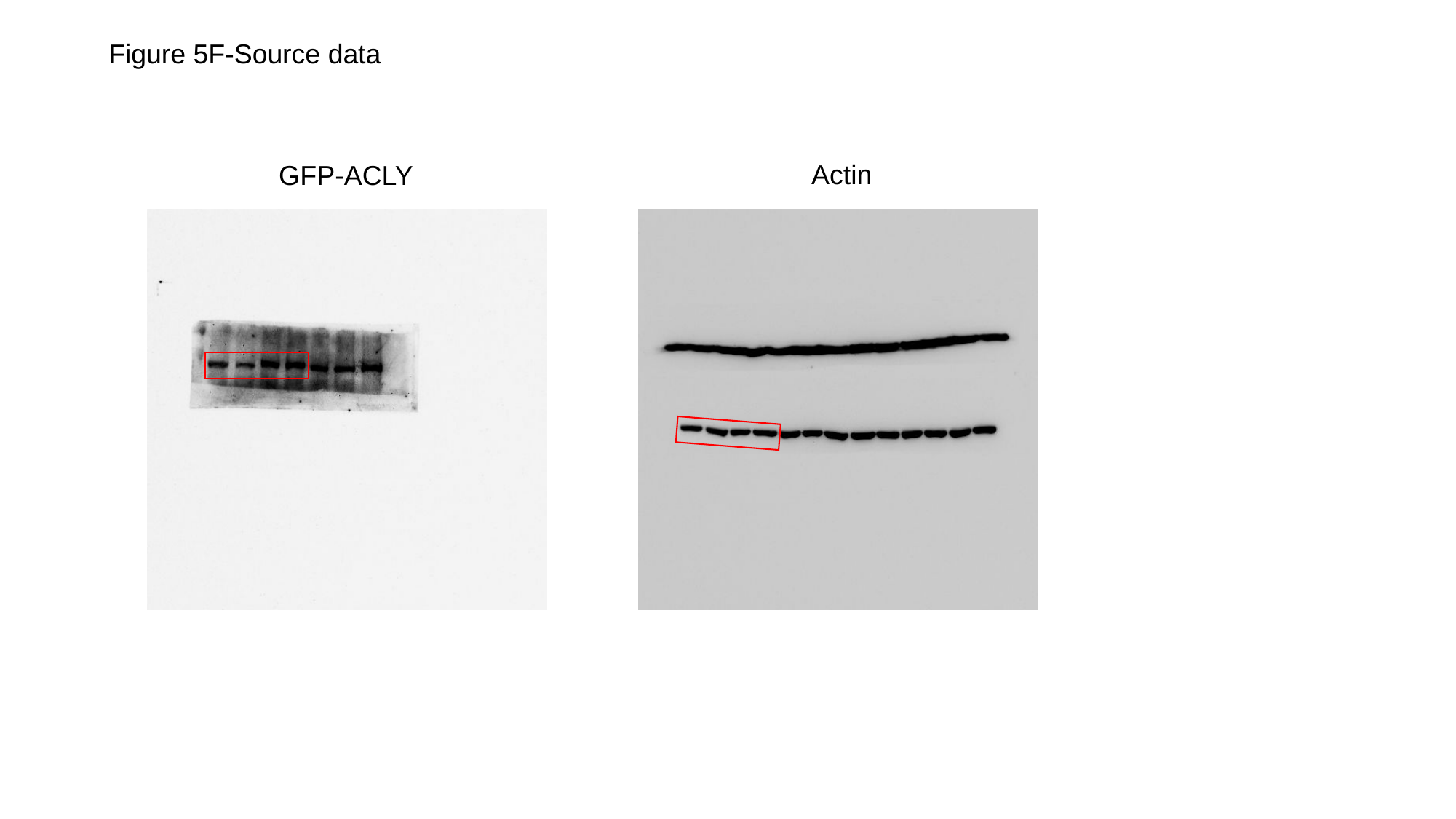

Figure 5F-Source data
Actin
GFP-ACLY
