# Supplementary figures and images for: ACLY ubiquitination by CUL3-KLHL25 induces the reprogramming of fatty acid metabolism to facilitate iTreg differentiation (part 2 of 2)
Source: eLife. 2021 Sep 7;10:e62394. doi: 10.7554/eLife.62394 (PMC8423445; doi:10.7554/eLife.62394)

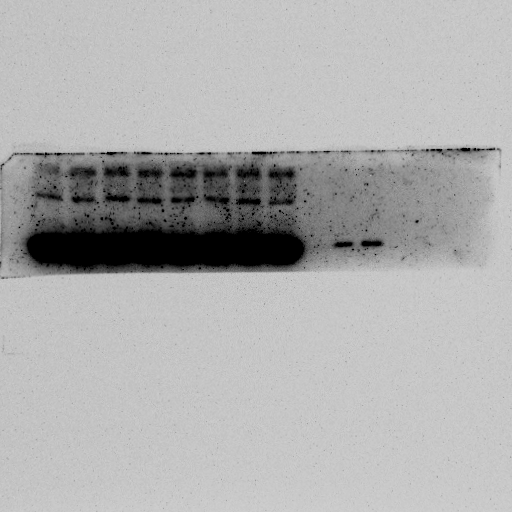

Supplement: Source data 1. [file elife-62394-data1.zip › Source data files/Source data (Raw)/Figure 5A-Source data-2(Actin).tif]

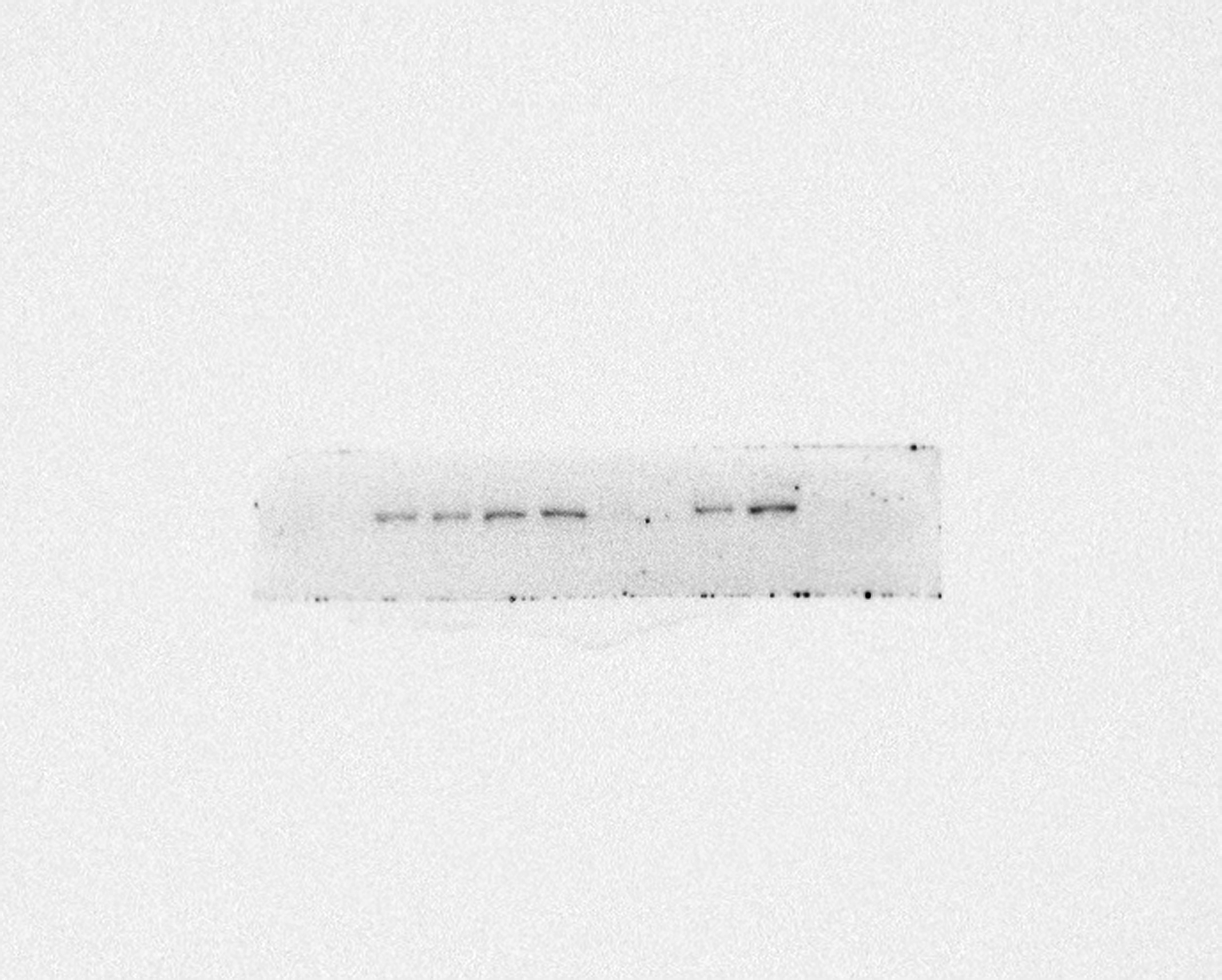

Supplement: Source data 1. [file elife-62394-data1.zip › Source data files/Source data (Raw)/Figure 5D-Source data-1(CUL3).tif]

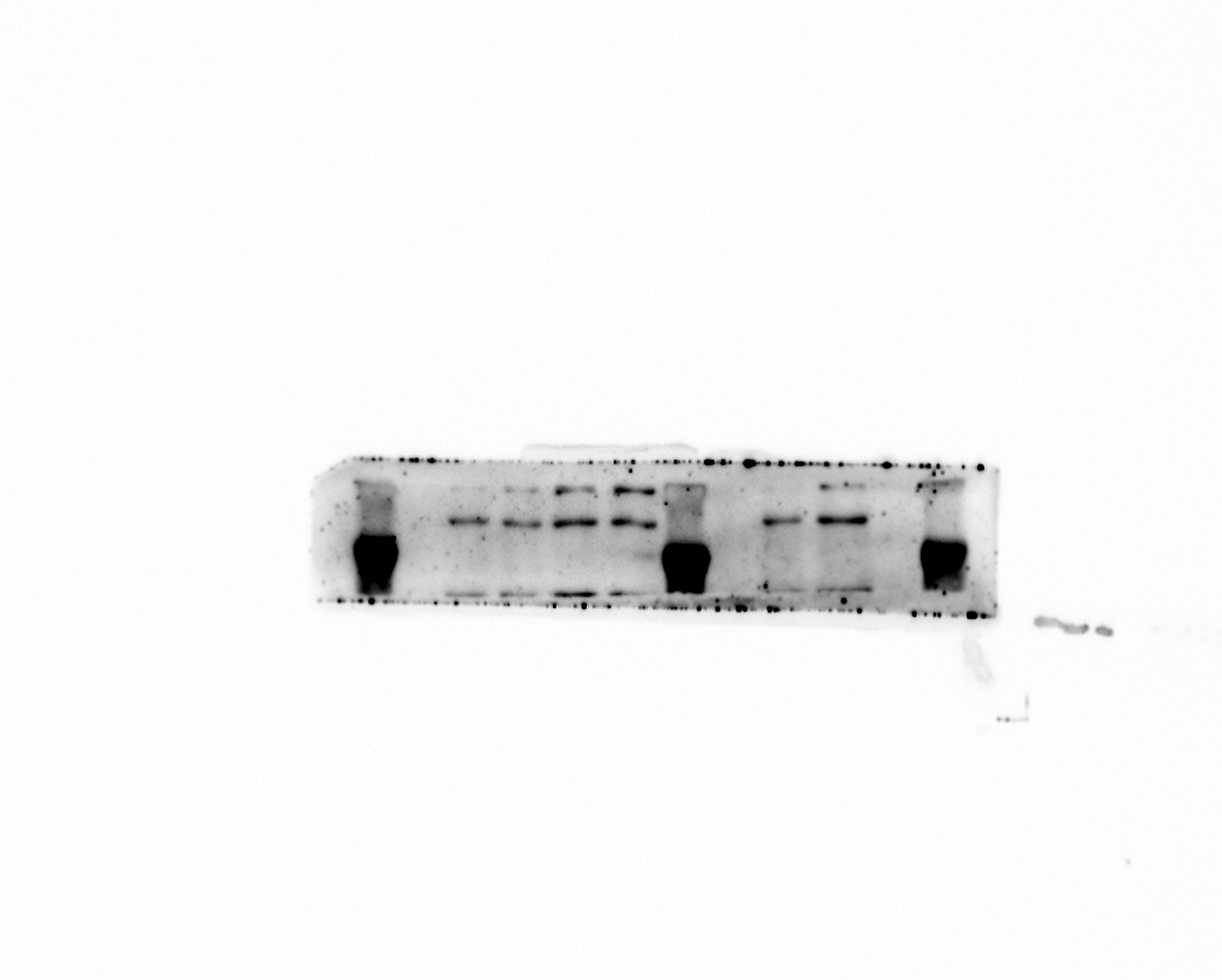

Supplement: Source data 1. [file elife-62394-data1.zip › Source data files/Source data (Raw)/Figure 5D-Source data-2(KLHL25).Tif]

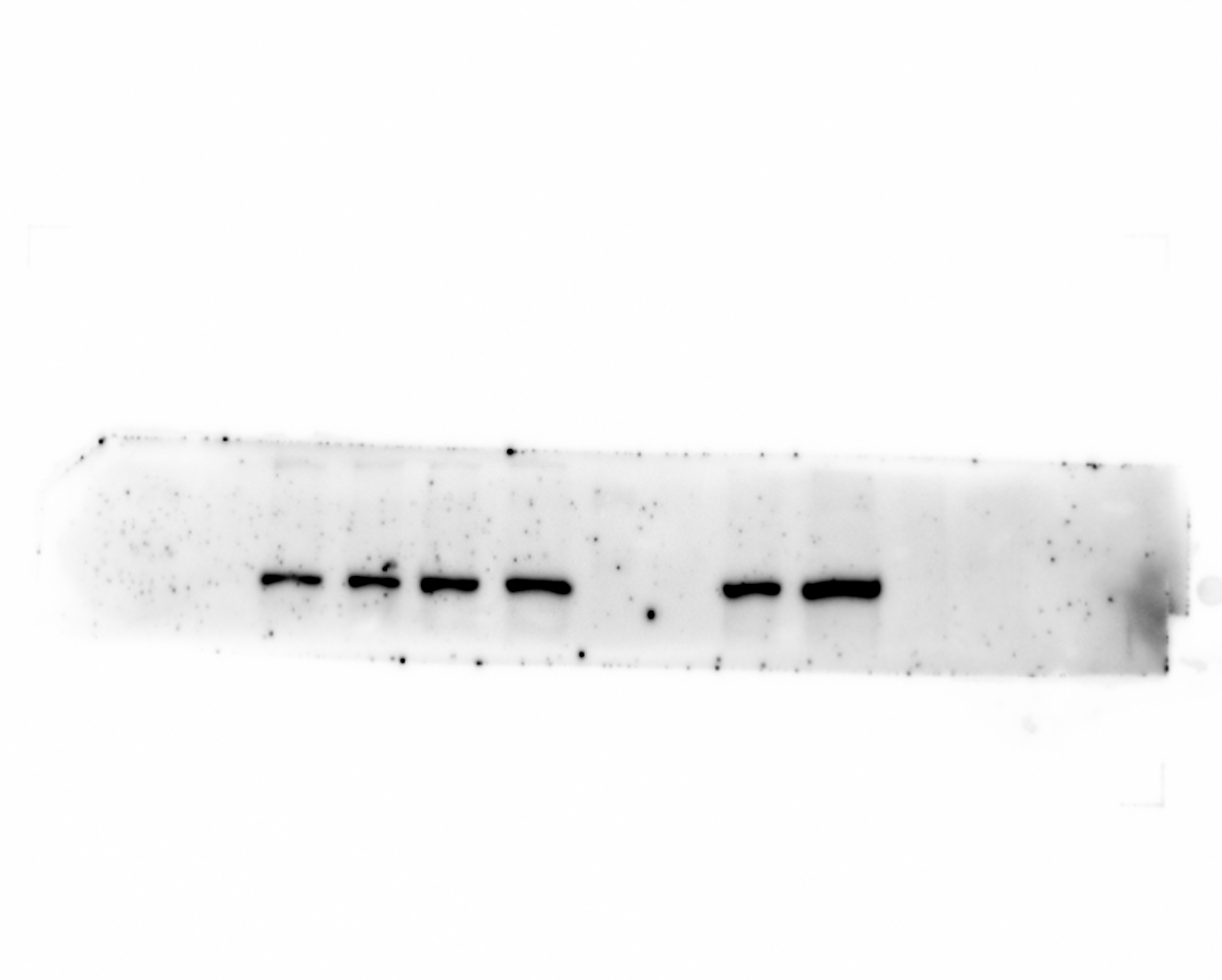

Supplement: Source data 1. [file elife-62394-data1.zip › Source data files/Source data (Raw)/Figure 5D-Source data-3(ACLY).Tif]

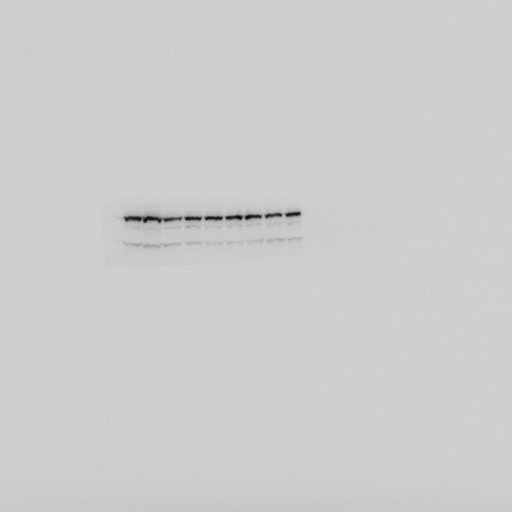

Supplement: Source data 1. [file elife-62394-data1.zip › Source data files/Source data (Raw)/Figure 5D-Source data-4(KLHL25).tif]

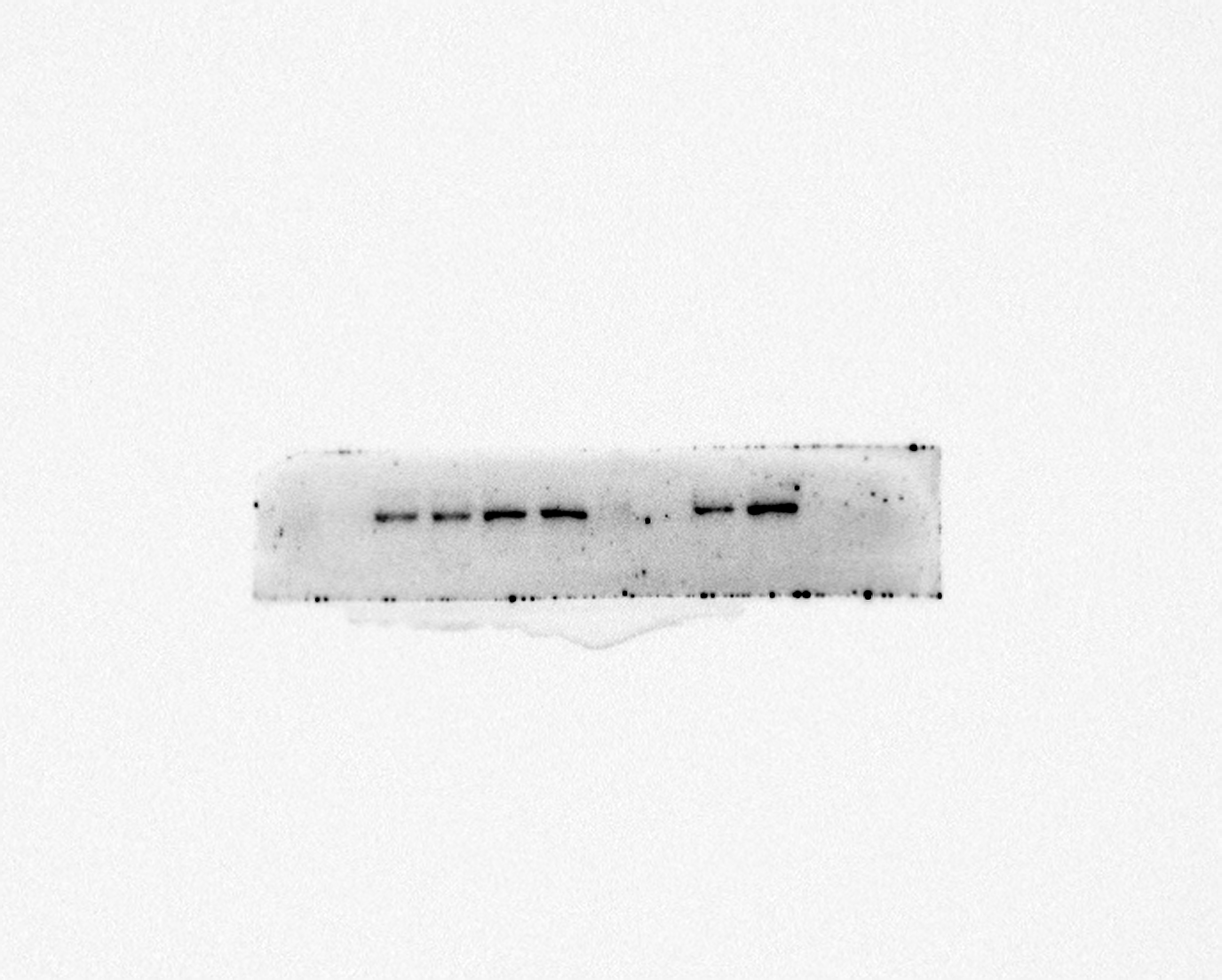

Supplement: Source data 1. [file elife-62394-data1.zip › Source data files/Source data (Raw)/Figure 5D-Source data-5(CUL3).Tif]

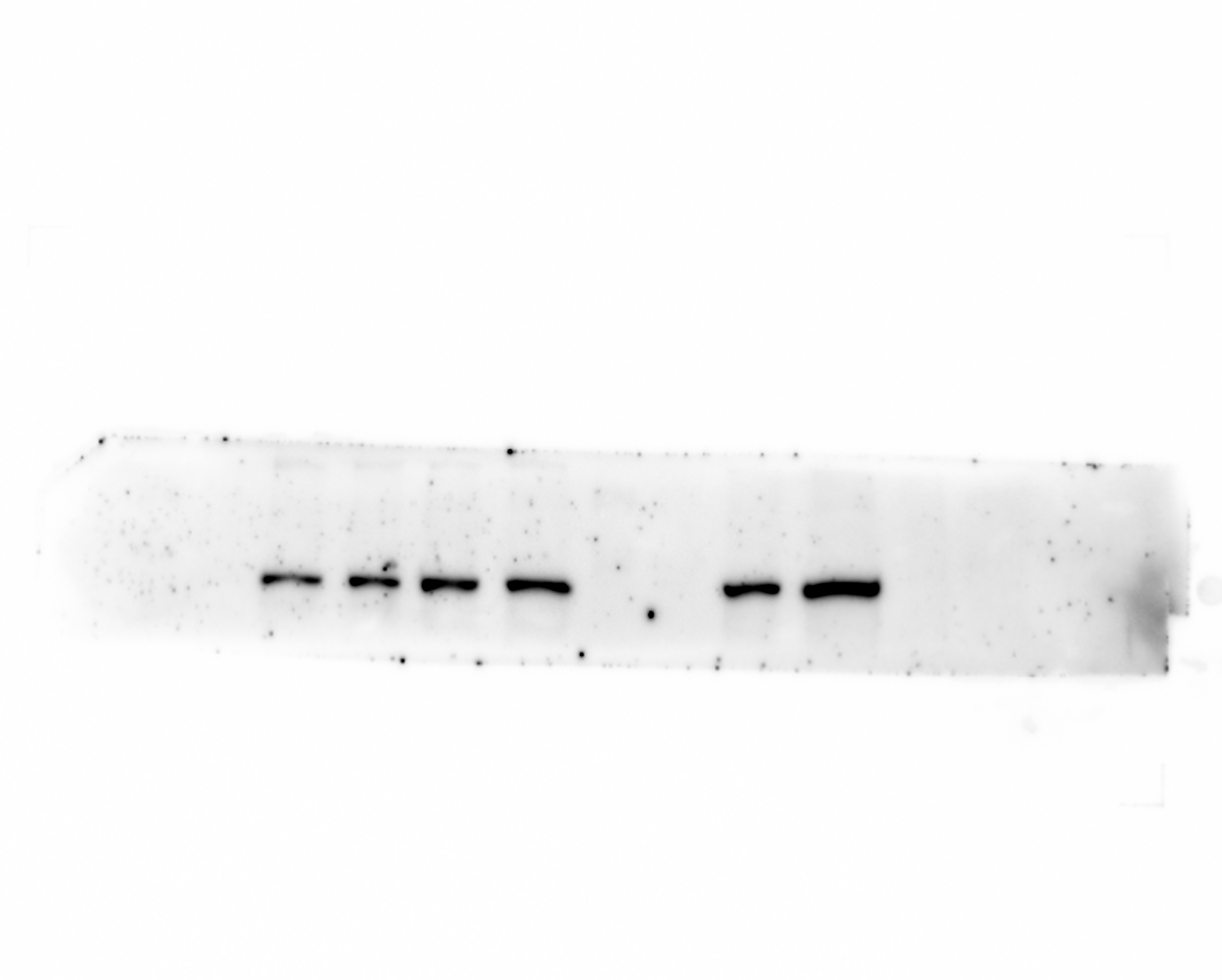

Supplement: Source data 1. [file elife-62394-data1.zip › Source data files/Source data (Raw)/Figure 5D-Source data-6(ACLY).Tif]

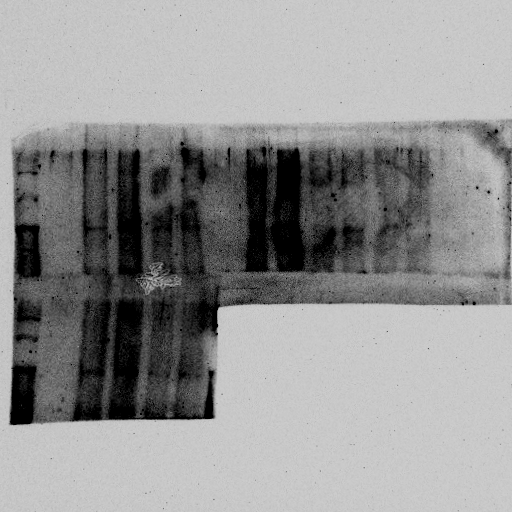

Supplement: Source data 1. [file elife-62394-data1.zip › Source data files/Source data (Raw)/Figure 5E-Source data-1(GFP-ACLY (Ub)n).tif]

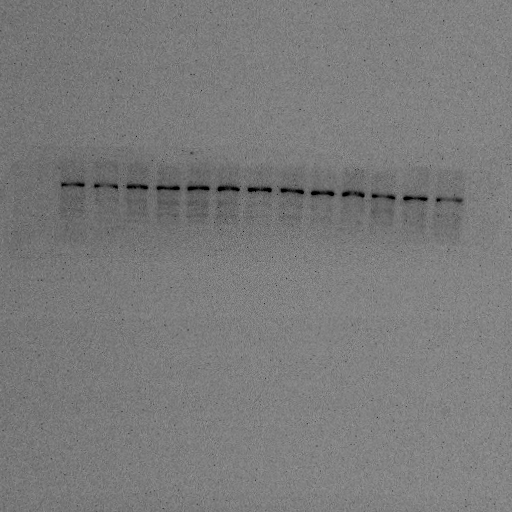

Supplement: Source data 1. [file elife-62394-data1.zip › Source data files/Source data (Raw)/Figure 5E-Source data-2(GFP-ACLY).tif]

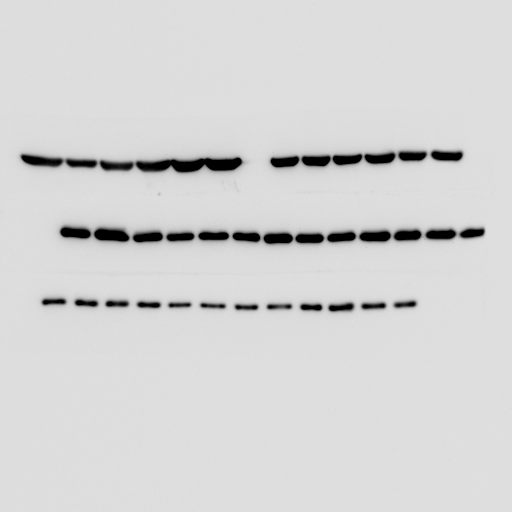

Supplement: Source data 1. [file elife-62394-data1.zip › Source data files/Source data (Raw)/Figure 5E-Source data-3(Actin).tif]

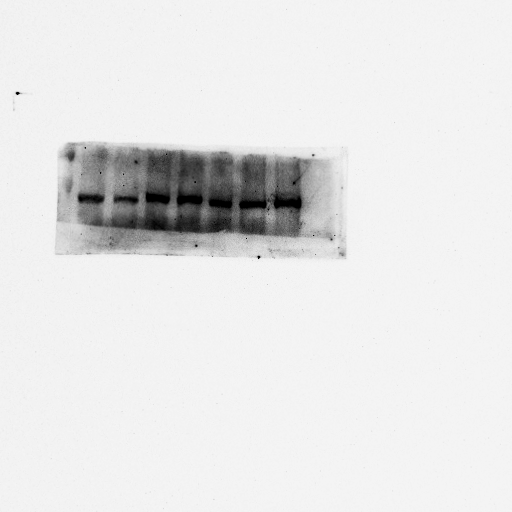

Supplement: Source data 1. [file elife-62394-data1.zip › Source data files/Source data (Raw)/Figure 5F-Source data-1(GFP-ACLY).tif]

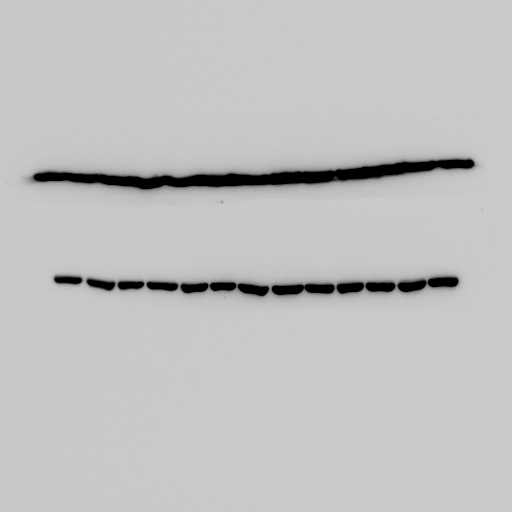

Supplement: Source data 1. [file elife-62394-data1.zip › Source data files/Source data (Raw)/Figure 5F-Source data-2(Actin).tif]
