# Supplementary figures and images for: ACLY ubiquitination by CUL3-KLHL25 induces the reprogramming of fatty acid metabolism to facilitate iTreg differentiation (part 1 of 2)
Source: eLife. 2021 Sep 7;10:e62394. doi: 10.7554/eLife.62394 (PMC8423445; doi:10.7554/eLife.62394)

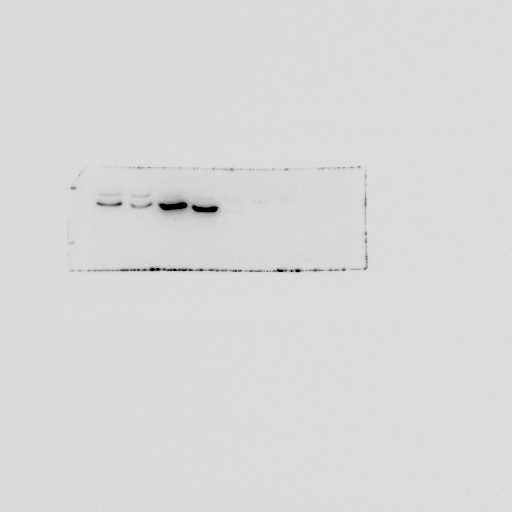

Supplement: Source data 1. [file elife-62394-data1.zip › Source data files/Source data (Raw)/Figure 1-figure supplement B-Source data-1(ATP5A).tif]

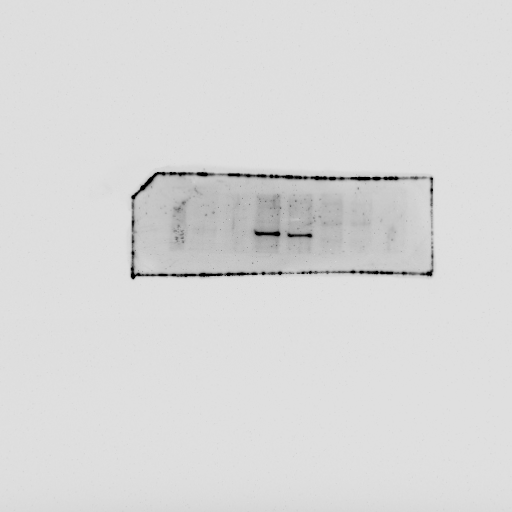

Supplement: Source data 1. [file elife-62394-data1.zip › Source data files/Source data (Raw)/Figure 1-figure supplement B-Source data-2(Lamin b).tif]

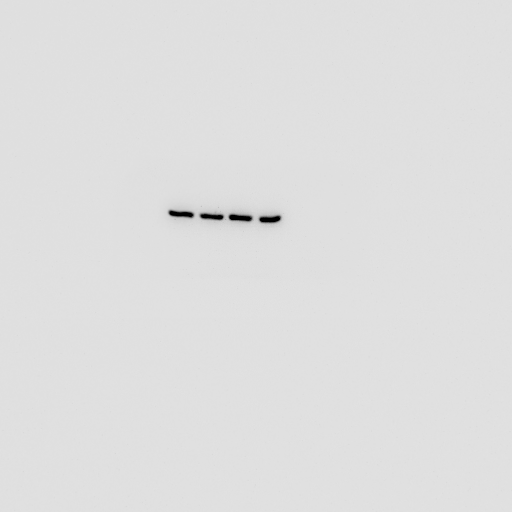

Supplement: Source data 1. [file elife-62394-data1.zip › Source data files/Source data (Raw)/Figure 1-figure supplement B-Source data-3(Tubulin).tif]

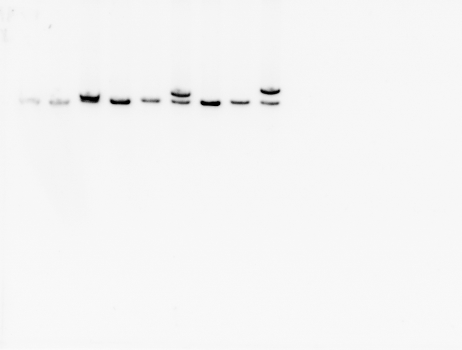

Supplement: Source data 1. [file elife-62394-data1.zip › Source data files/Source data (Raw)/Figure 1-figure supplement C-Source data-1(ACLY).tif]

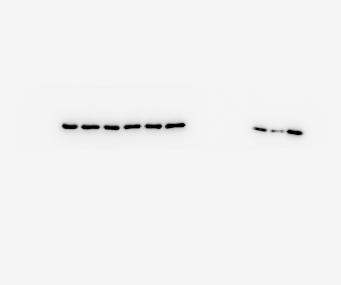

Supplement: Source data 1. [file elife-62394-data1.zip › Source data files/Source data (Raw)/Figure 1-figure supplement C-Source data-2(Actin).tif]

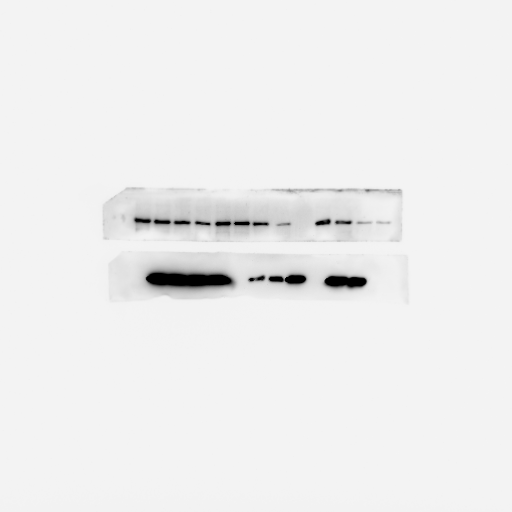

Supplement: Source data 1. [file elife-62394-data1.zip › Source data files/Source data (Raw)/Figure 1-figure supplement D-Source data-1(ACLY).tif]

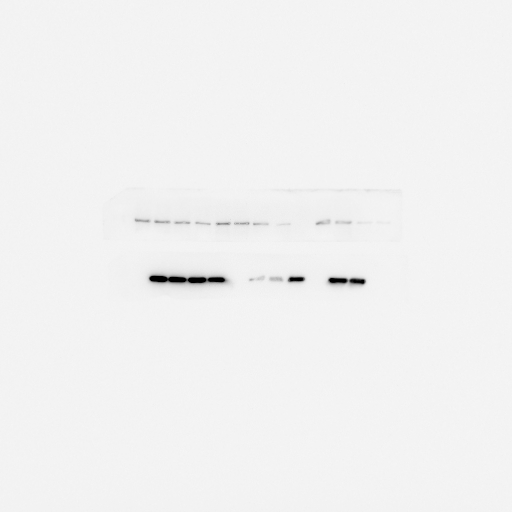

Supplement: Source data 1. [file elife-62394-data1.zip › Source data files/Source data (Raw)/Figure 1-figure supplement D-Source data-2(Actin).tif]

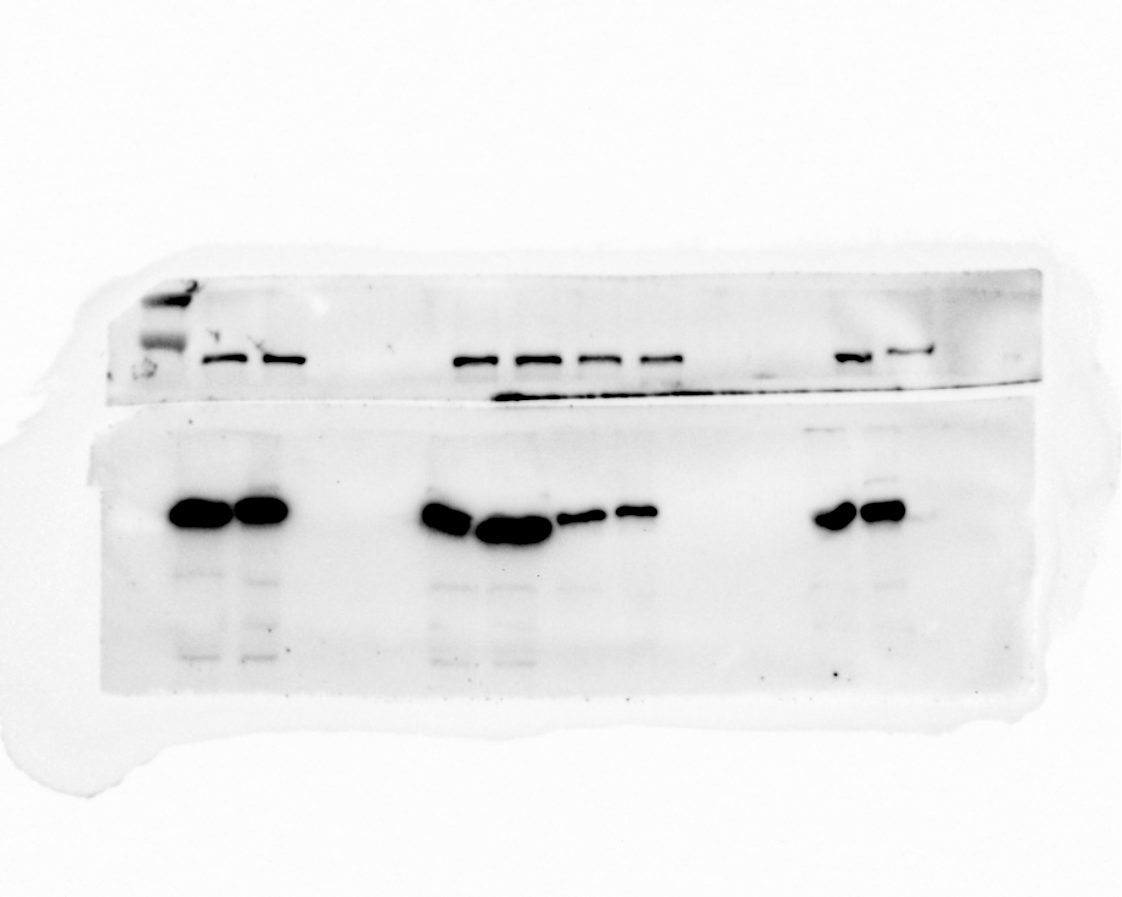

Supplement: Source data 1. [file elife-62394-data1.zip › Source data files/Source data (Raw)/Figure 2-figure supplement 3A-Source data-1(Lamin b1).tif]

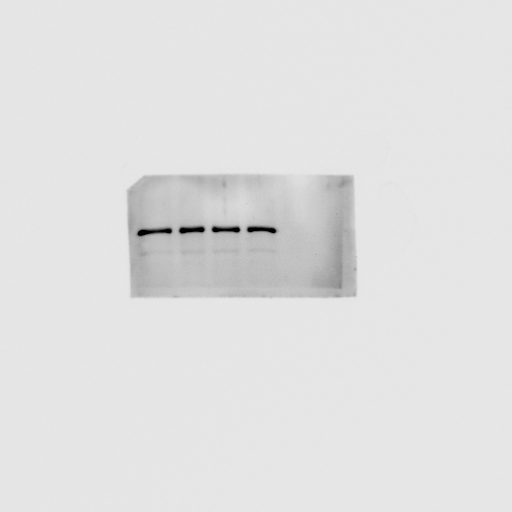

Supplement: Source data 1. [file elife-62394-data1.zip › Source data files/Source data (Raw)/Figure 2-figure supplement 3A-Source data-2(Tubulin).tif]

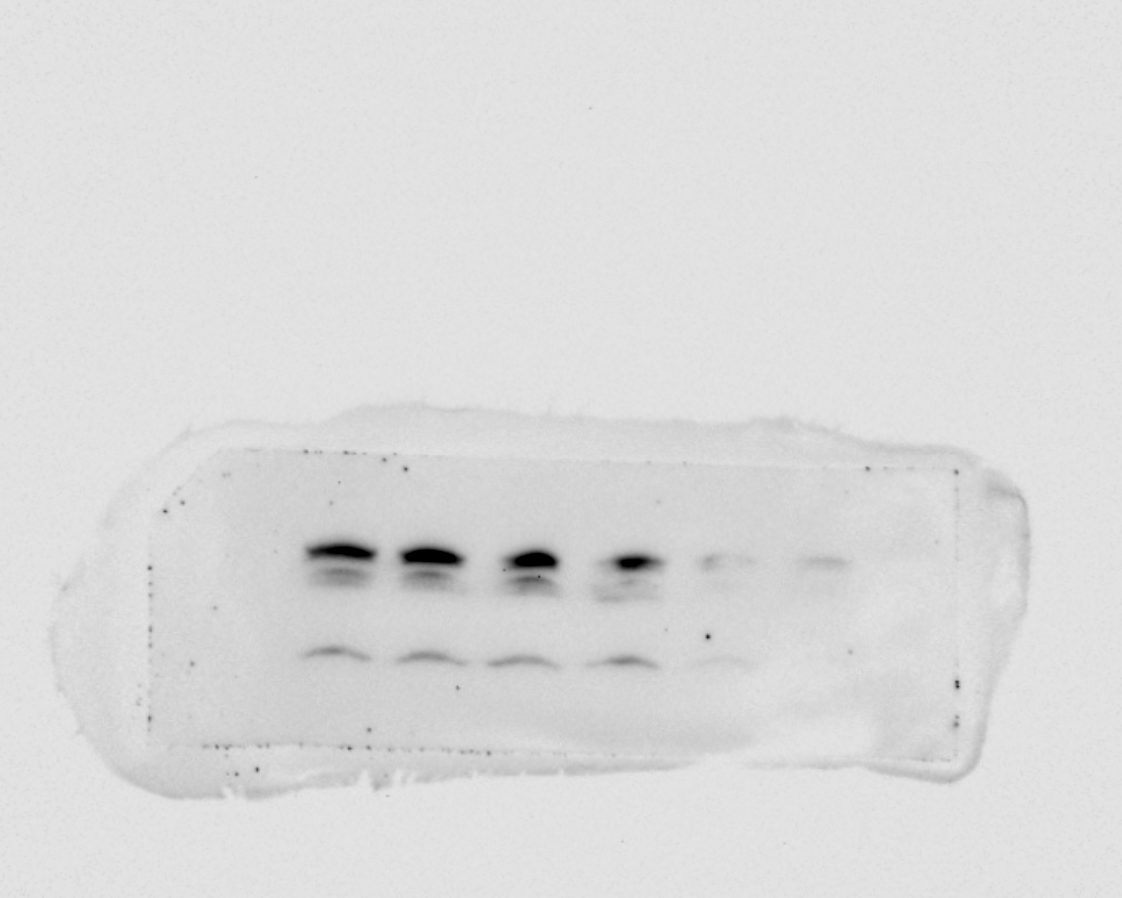

Supplement: Source data 1. [file elife-62394-data1.zip › Source data files/Source data (Raw)/Figure 2-figure supplement 3C-Source data-1(Acetylation).tif]

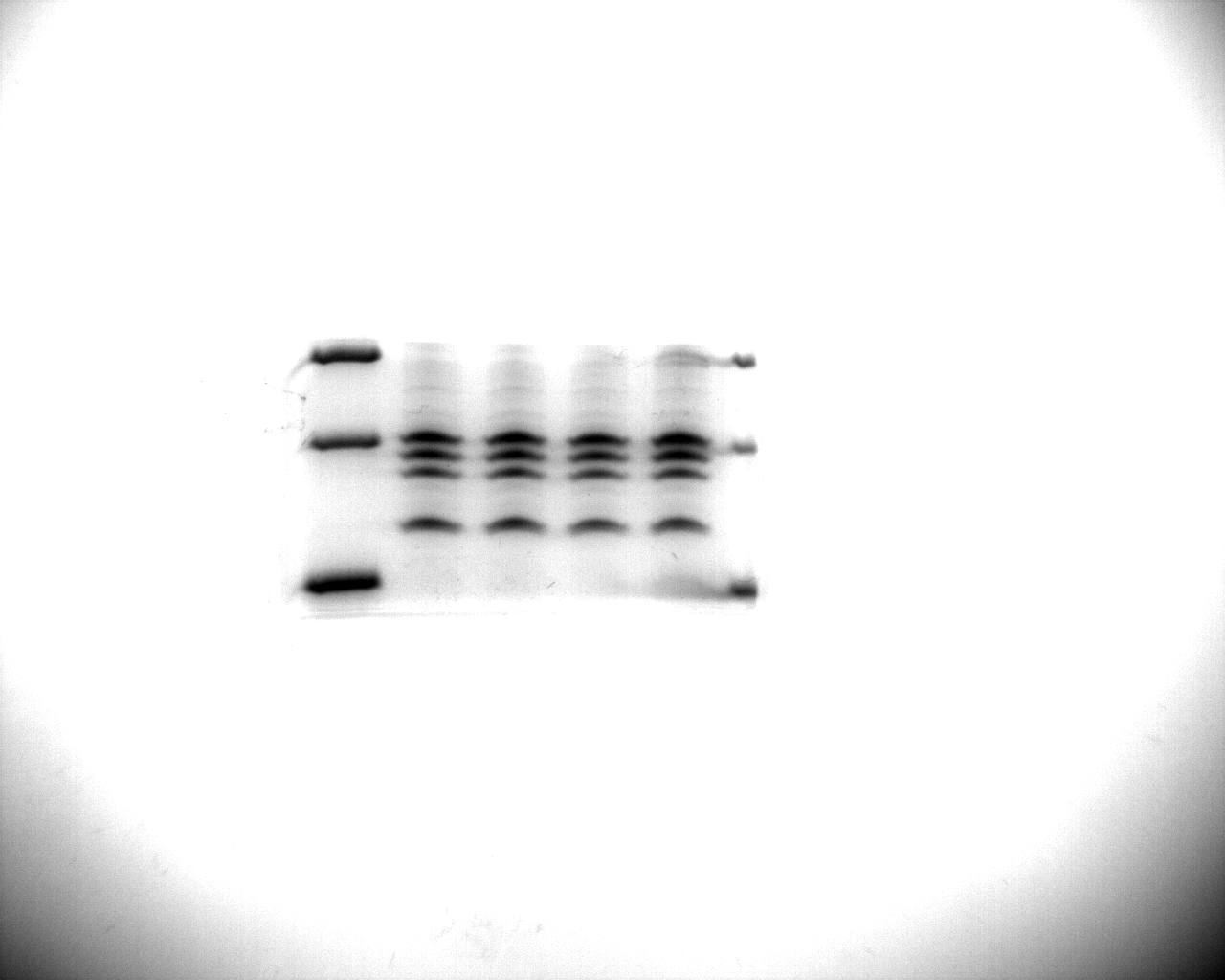

Supplement: Source data 1. [file elife-62394-data1.zip › Source data files/Source data (Raw)/Figure 2-figure supplement 3C-Source data-2(Coomassie).Tif]

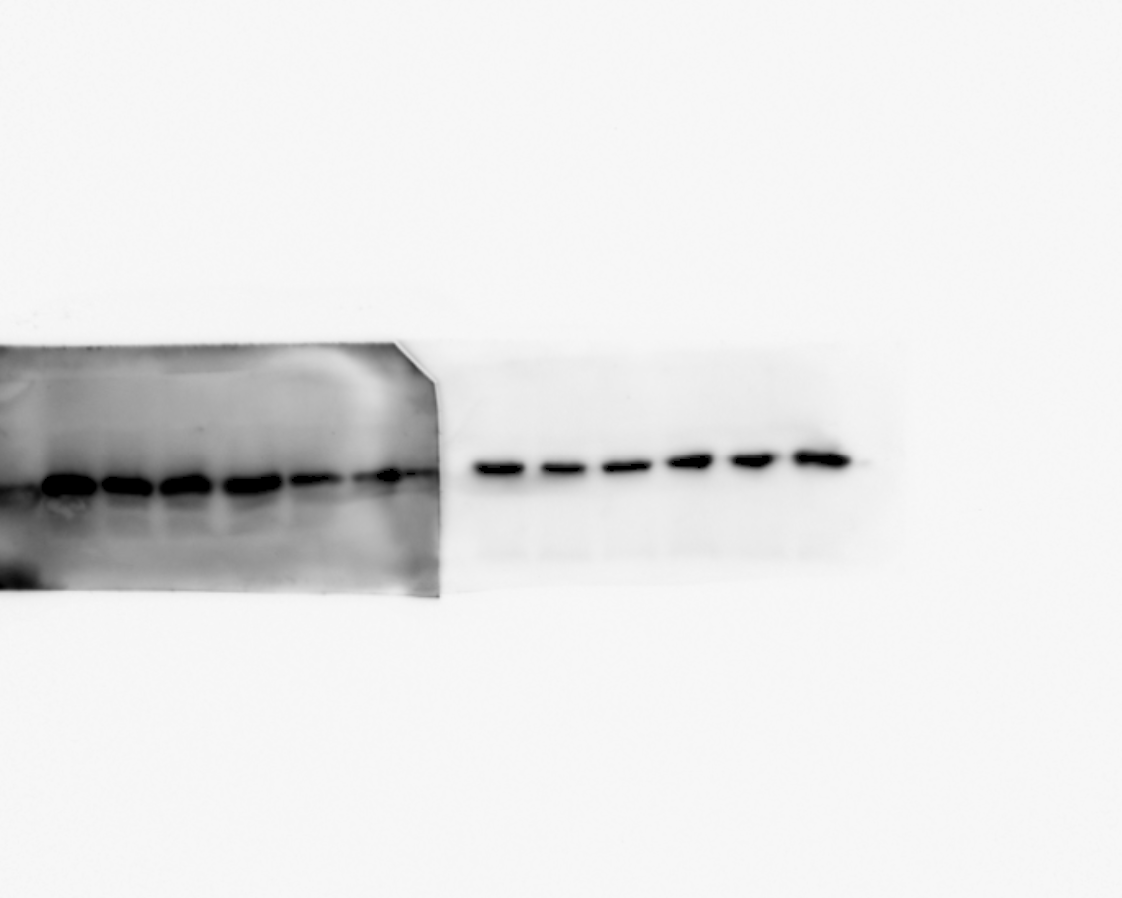

Supplement: Source data 1. [file elife-62394-data1.zip › Source data files/Source data (Raw)/Figure 2-figure supplement 3D-Source data-1(H3Ac).tif]

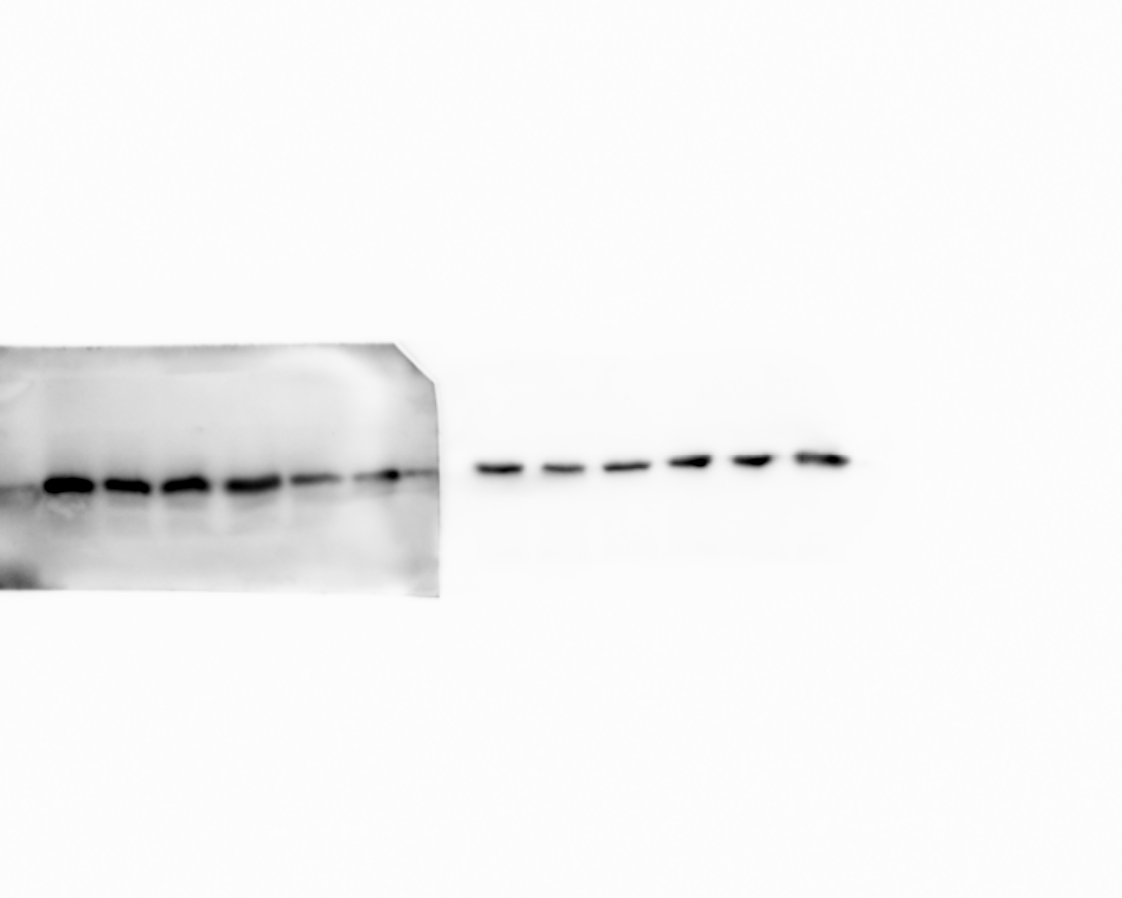

Supplement: Source data 1. [file elife-62394-data1.zip › Source data files/Source data (Raw)/Figure 2-figure supplement 3D-Source data-2(H3).tif]

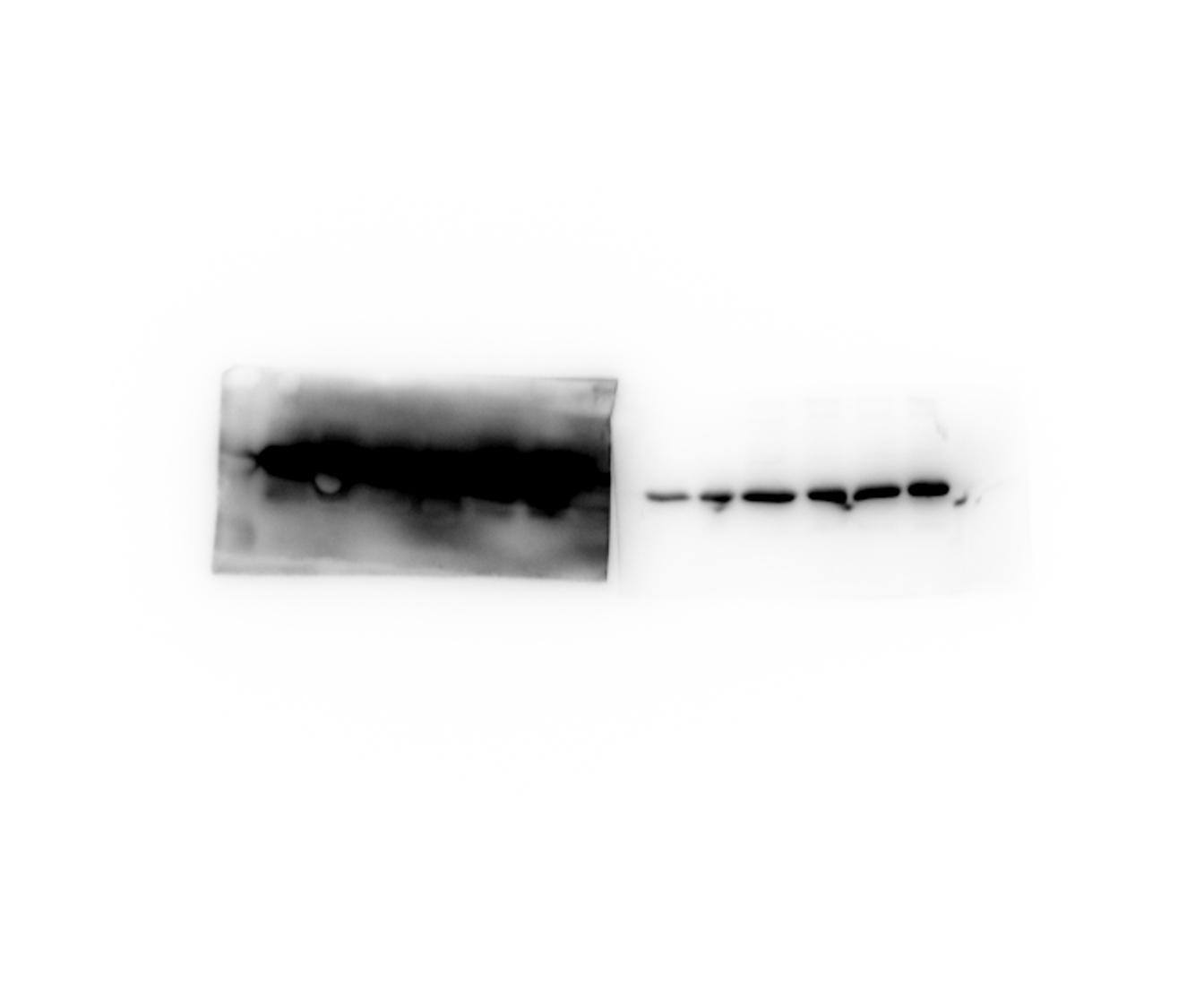

Supplement: Source data 1. [file elife-62394-data1.zip › Source data files/Source data (Raw)/Figure 2-figure supplement 3D-Source data-3(H4Ac).Tif]

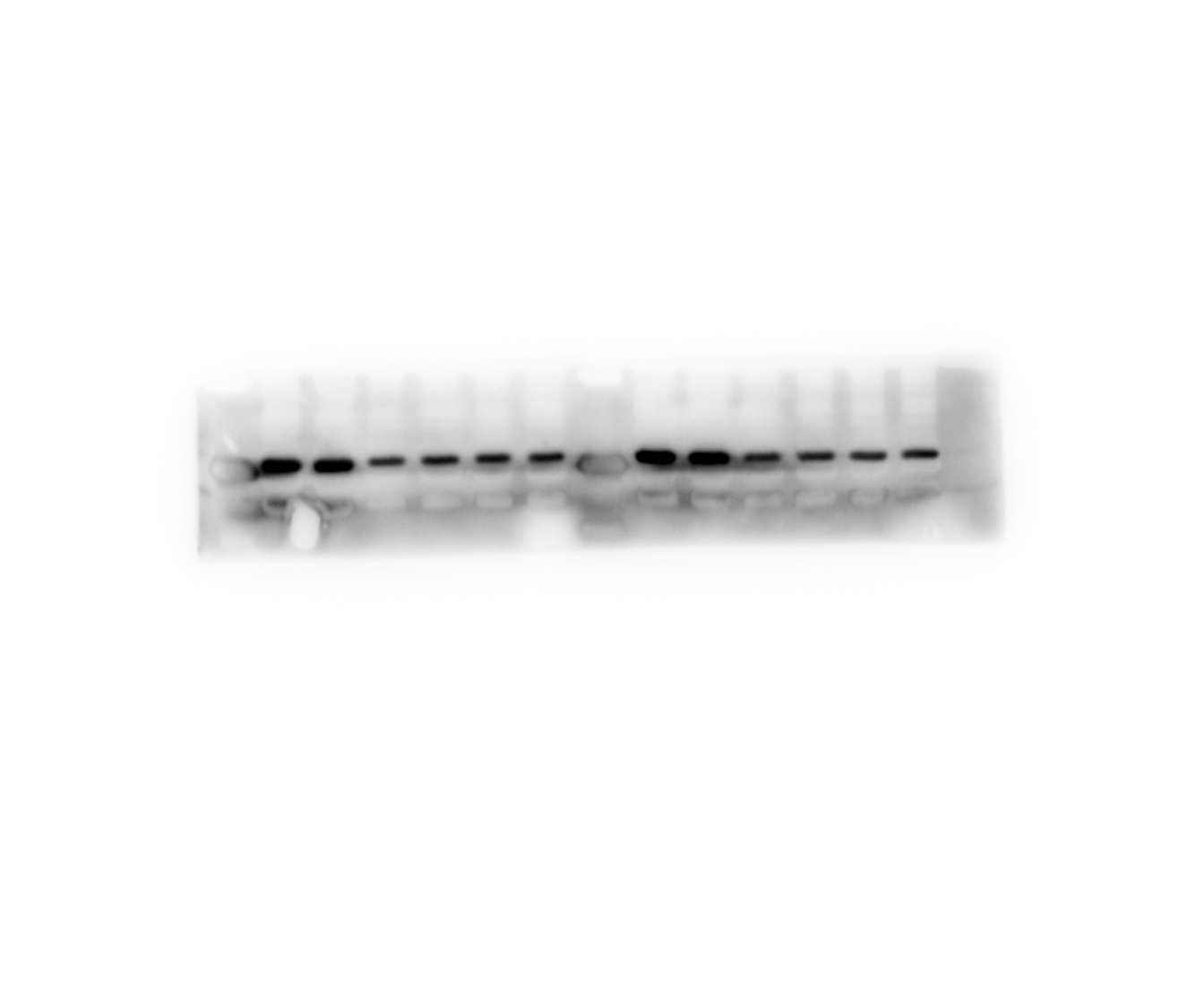

Supplement: Source data 1. [file elife-62394-data1.zip › Source data files/Source data (Raw)/Figure 2-figure supplement 3D-Source data-4(H4).Tif]

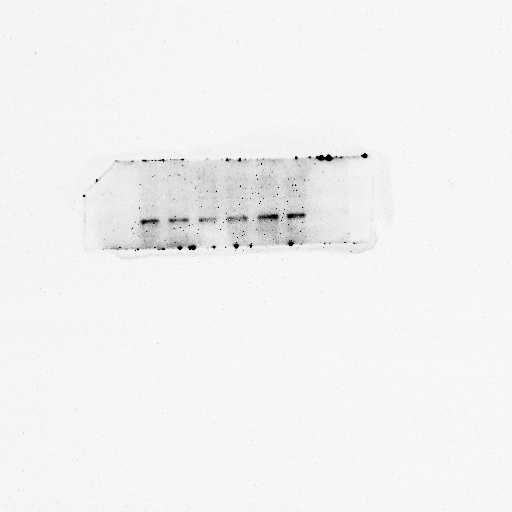

Supplement: Source data 1. [file elife-62394-data1.zip › Source data files/Source data (Raw)/Figure 2F-Source data-1(CPT1)1.tif]

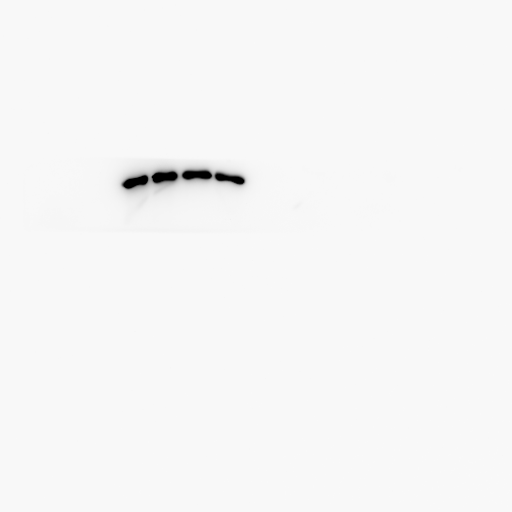

Supplement: Source data 1. [file elife-62394-data1.zip › Source data files/Source data (Raw)/Figure 2F-Source data-2(Actin).tif]

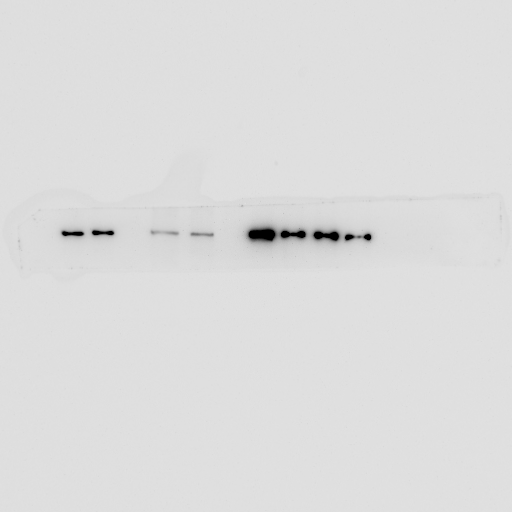

Supplement: Source data 1. [file elife-62394-data1.zip › Source data files/Source data (Raw)/Figure 3-figure supplement 1A-Source data-1 (ACLY).tif]

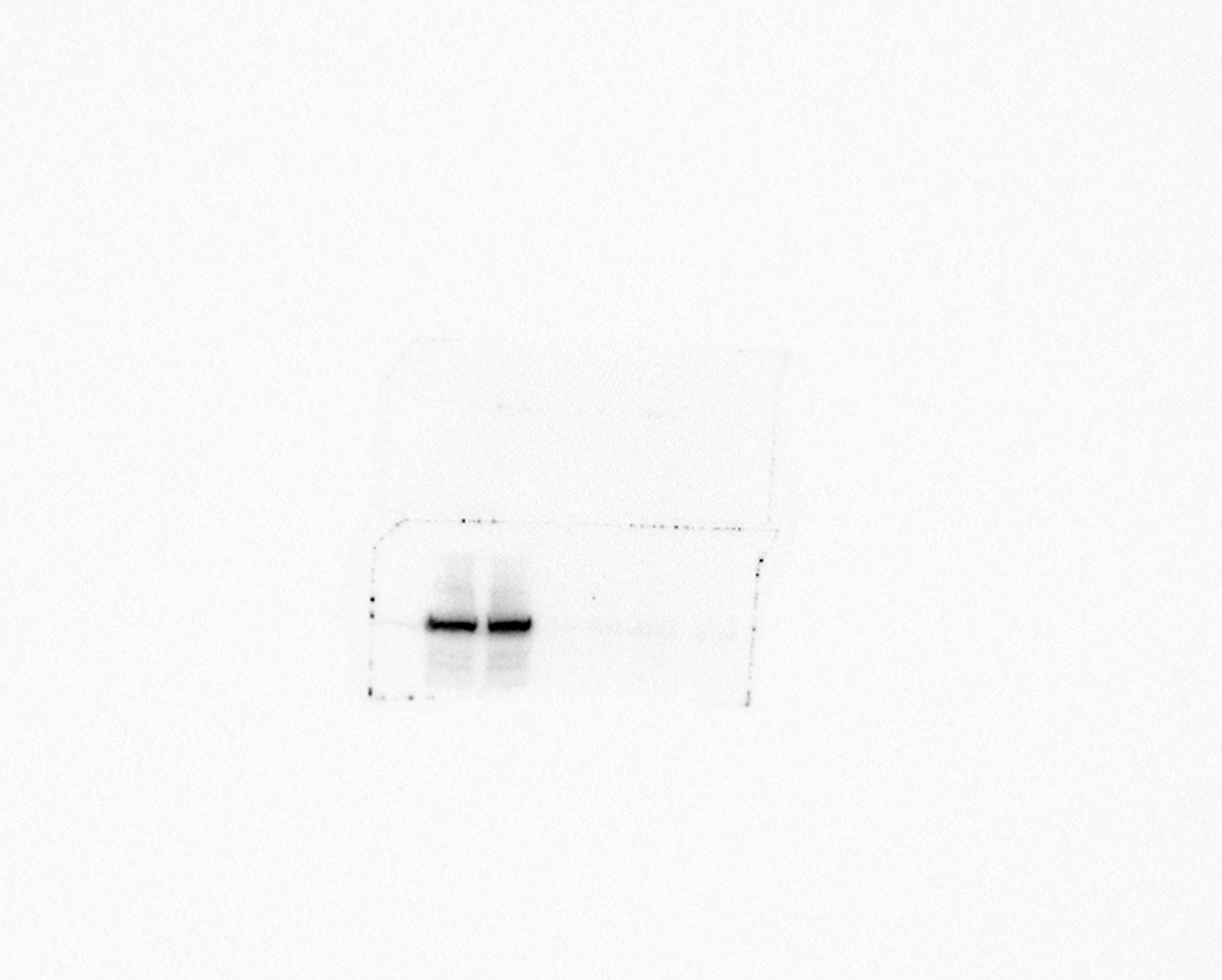

Supplement: Source data 1. [file elife-62394-data1.zip › Source data files/Source data (Raw)/Figure 3-figure supplement 1B-Source data-1 (ACLY-P(Ser 455)).Tif]

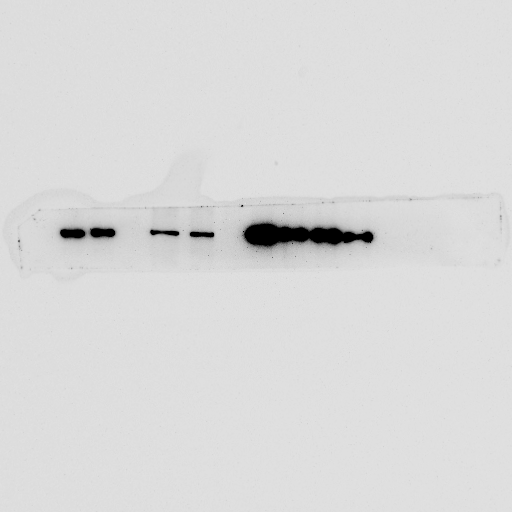

Supplement: Source data 1. [file elife-62394-data1.zip › Source data files/Source data (Raw)/Figure 3-figure supplement 1B-Source data-2 (ACLY).tif]

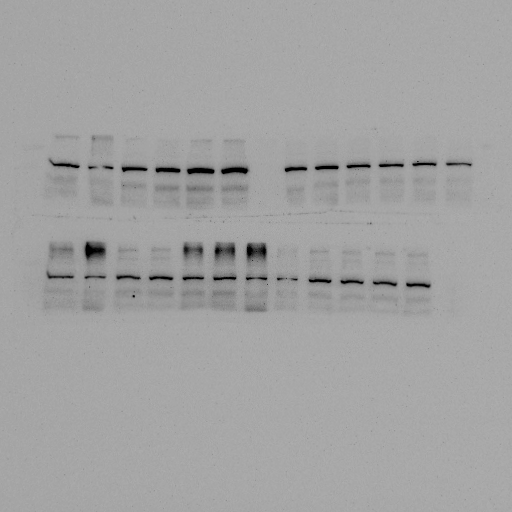

Supplement: Source data 1. [file elife-62394-data1.zip › Source data files/Source data (Raw)/Figure 3-figure supplement 2B-Source data-1 (GFP-ACLY).tif]

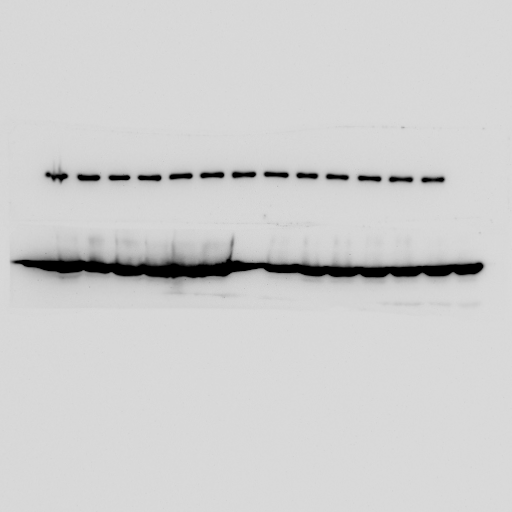

Supplement: Source data 1. [file elife-62394-data1.zip › Source data files/Source data (Raw)/Figure 3-figure supplement 2B-Source data-2 (Actin).tif]

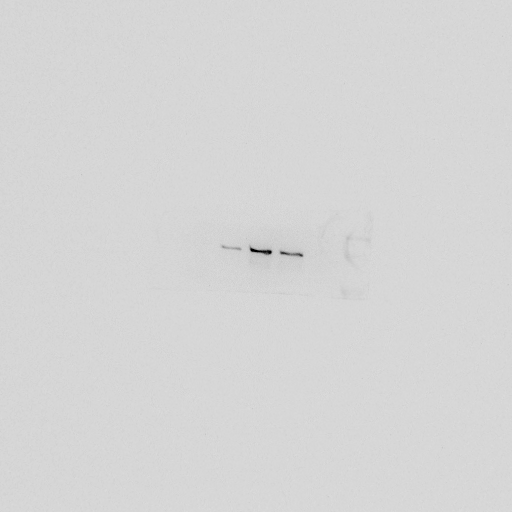

Supplement: Source data 1. [file elife-62394-data1.zip › Source data files/Source data (Raw)/Figure 3A-Source data-1(ACLY).tif]

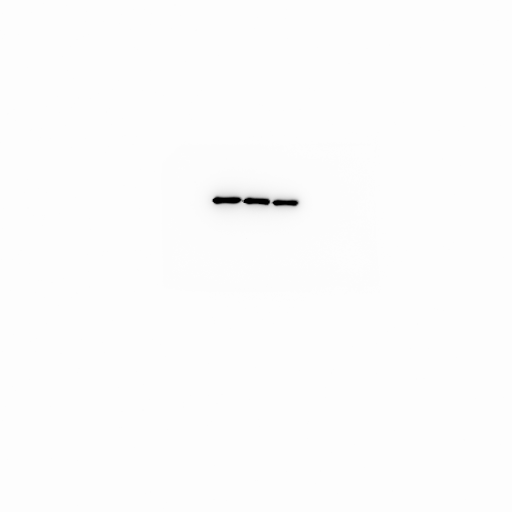

Supplement: Source data 1. [file elife-62394-data1.zip › Source data files/Source data (Raw)/Figure 3A-Source data-2(Actin).tif]

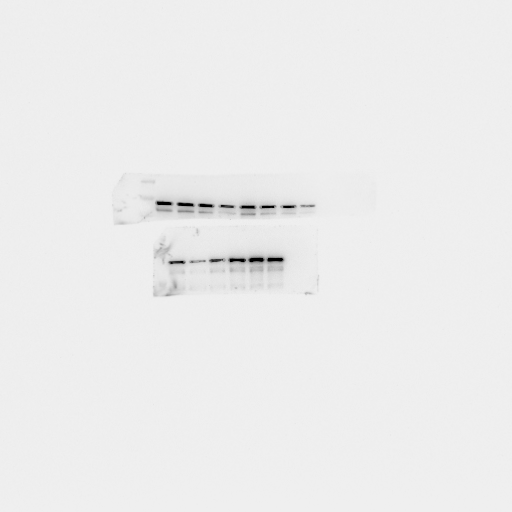

Supplement: Source data 1. [file elife-62394-data1.zip › Source data files/Source data (Raw)/Figure 3B-Source data-1(ACLY).tif]

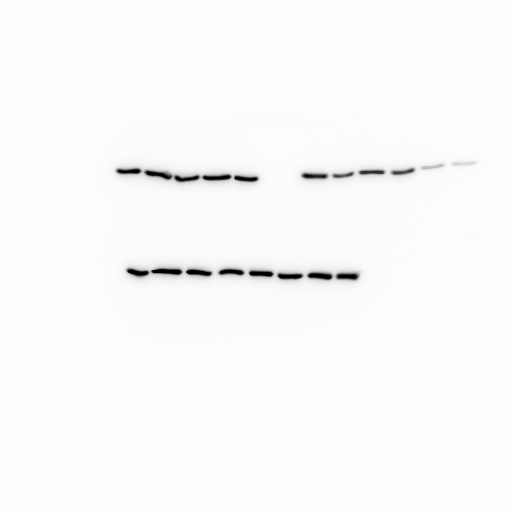

Supplement: Source data 1. [file elife-62394-data1.zip › Source data files/Source data (Raw)/Figure 3B-Source data-2(Actin).tif]

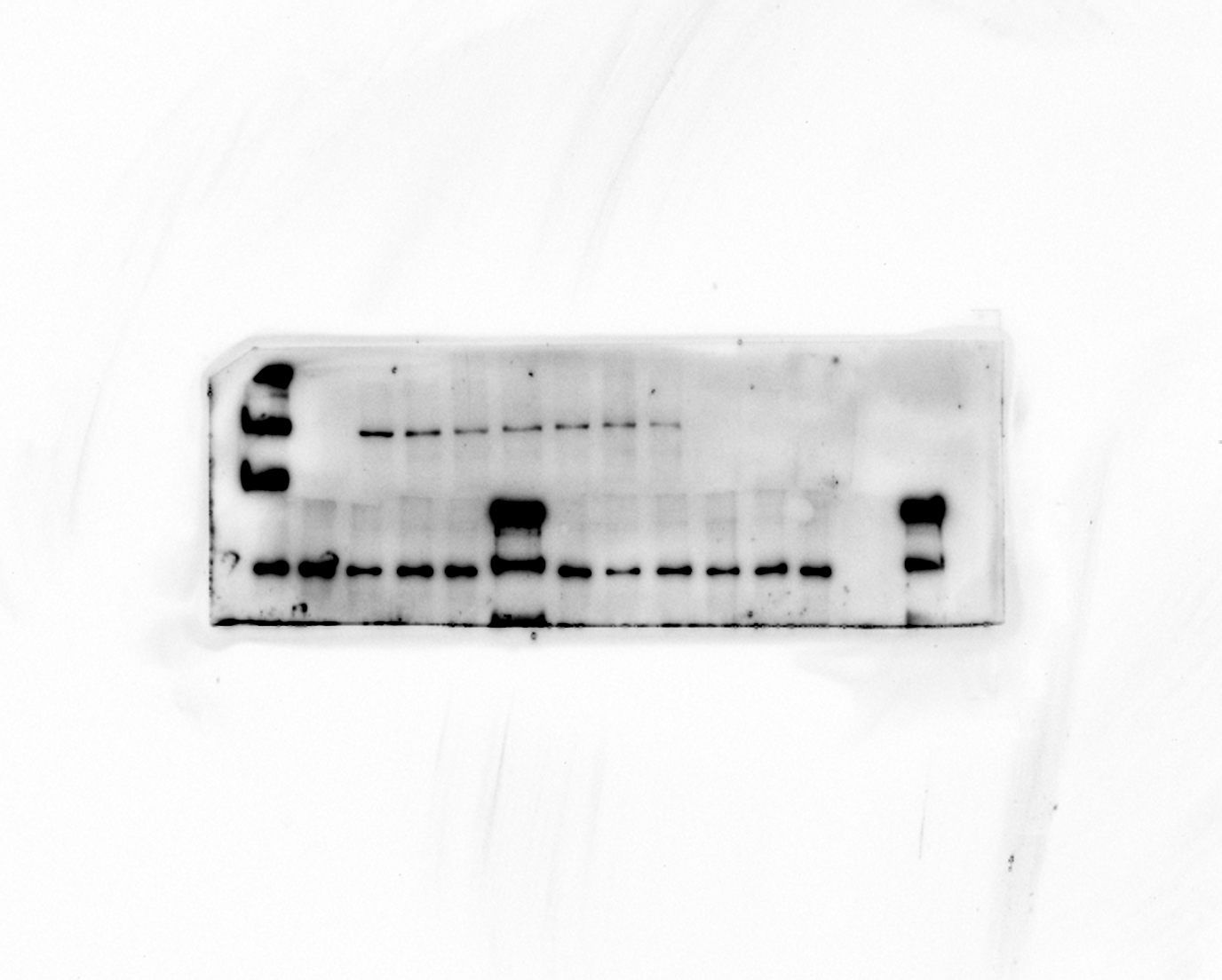

Supplement: Source data 1. [file elife-62394-data1.zip › Source data files/Source data (Raw)/Figure 3C-Source data-1 (ACLY).Tif]

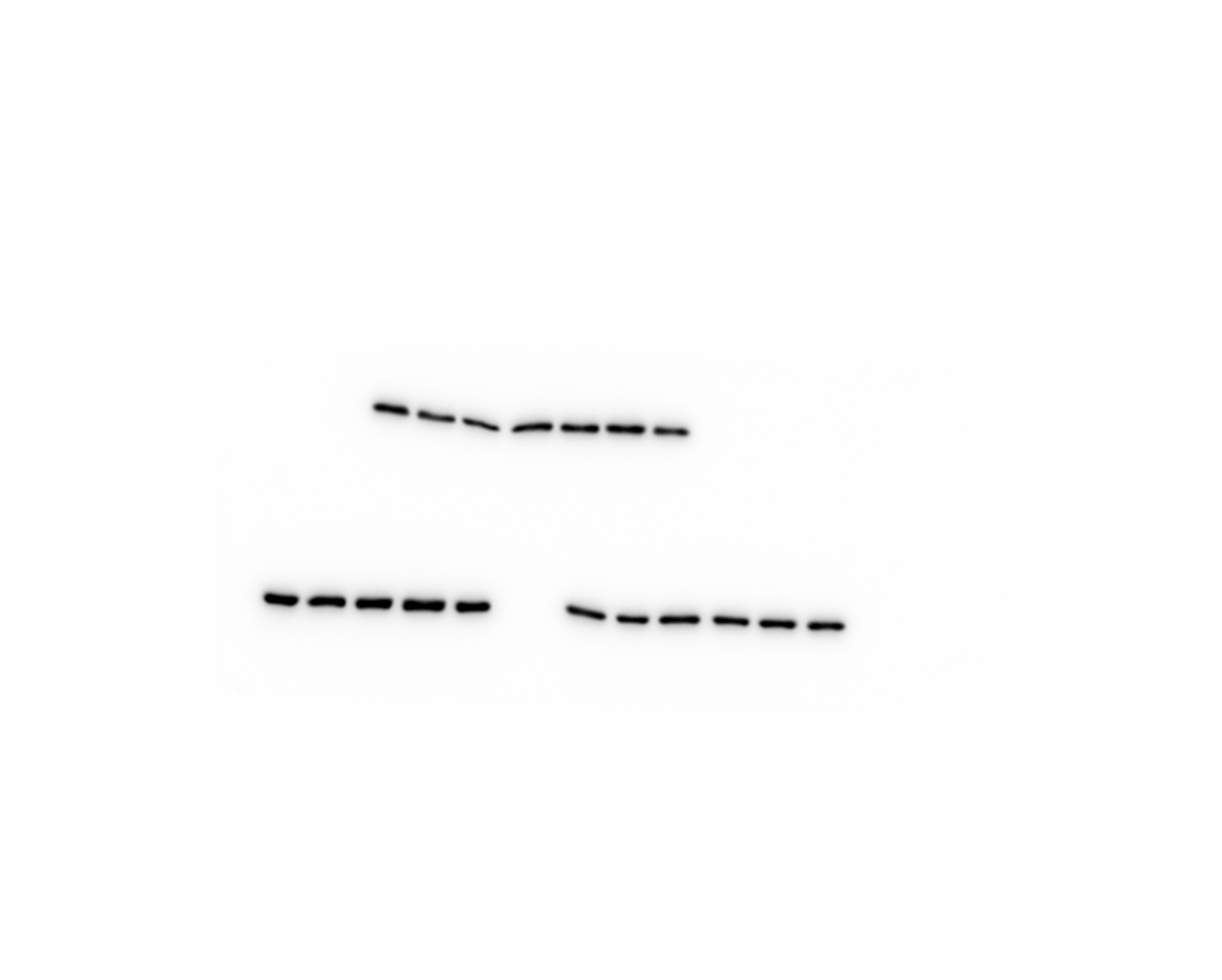

Supplement: Source data 1. [file elife-62394-data1.zip › Source data files/Source data (Raw)/Figure 3C-Source data-2 (Actin).Tif]

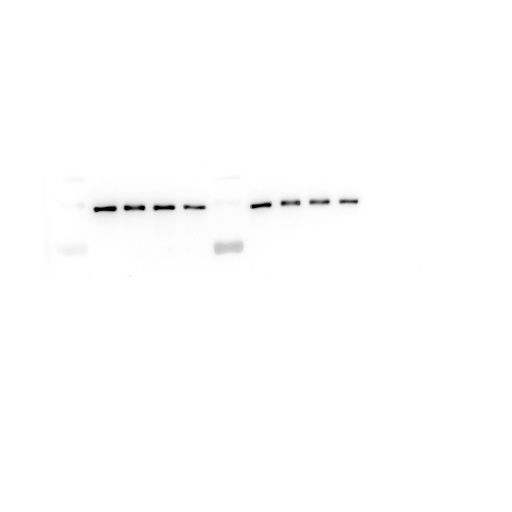

Supplement: Source data 1. [file elife-62394-data1.zip › Source data files/Source data (Raw)/Figure 3C-Source data-3 (ACLY).tif]

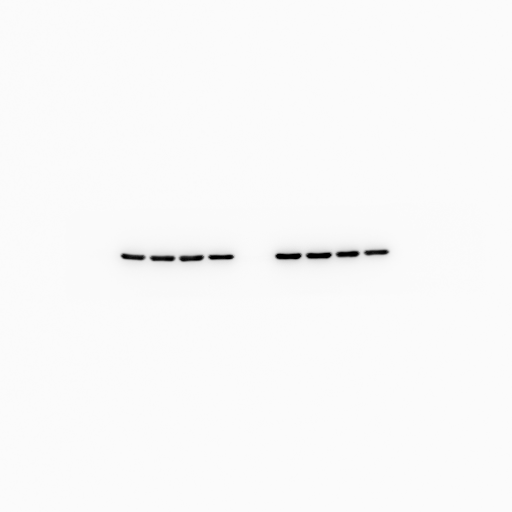

Supplement: Source data 1. [file elife-62394-data1.zip › Source data files/Source data (Raw)/Figure 3C-Source data-4 (Actin).tif]

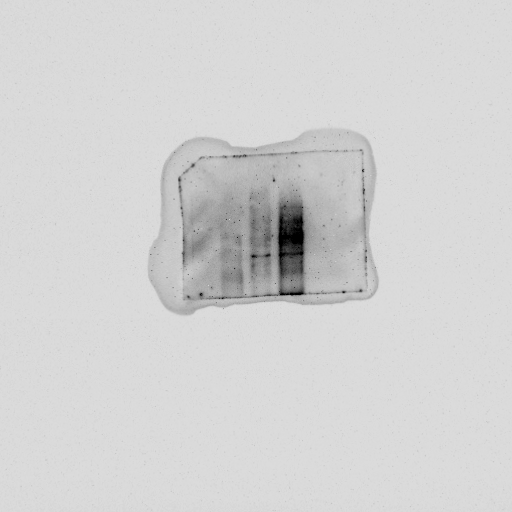

Supplement: Source data 1. [file elife-62394-data1.zip › Source data files/Source data (Raw)/Figure 3D-Source data-1(ACLY-(Ub)n).tif]

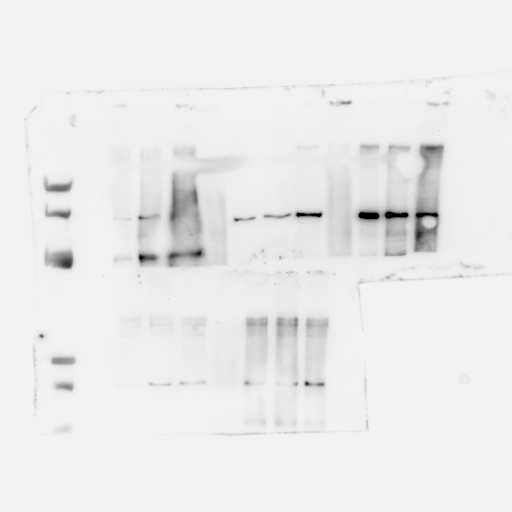

Supplement: Source data 1. [file elife-62394-data1.zip › Source data files/Source data (Raw)/Figure 3D-Source data-2(ACLY).tif]

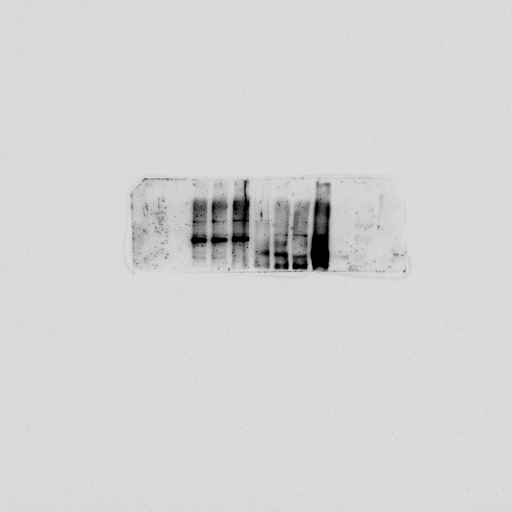

Supplement: Source data 1. [file elife-62394-data1.zip › Source data files/Source data (Raw)/Figure 3D-Source data-3(Ub).tif]

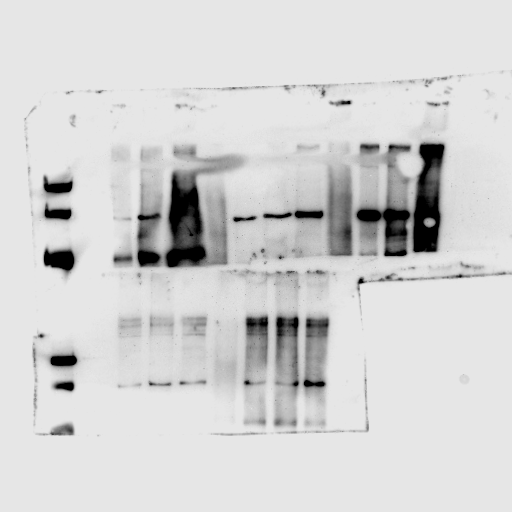

Supplement: Source data 1. [file elife-62394-data1.zip › Source data files/Source data (Raw)/Figure 3D-Source data-4(ACLY).tif]

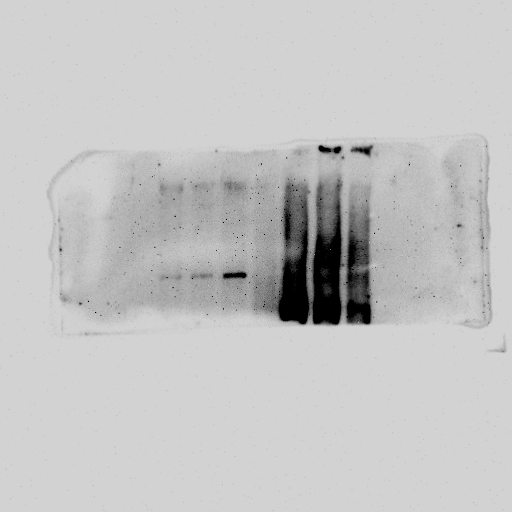

Supplement: Source data 1. [file elife-62394-data1.zip › Source data files/Source data (Raw)/Figure 3E-Source data-1(ACLY).tif]

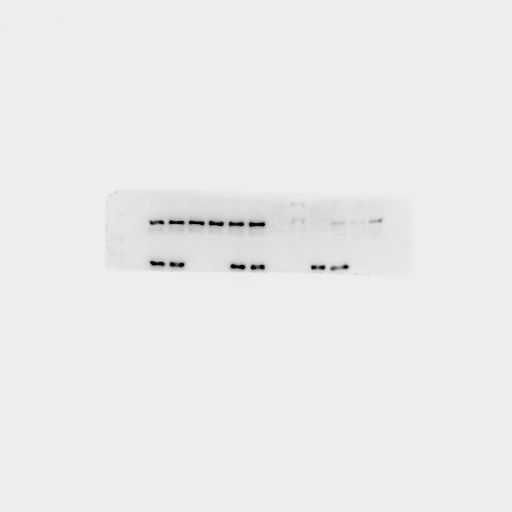

Supplement: Source data 1. [file elife-62394-data1.zip › Source data files/Source data (Raw)/Figure 3E-Source data-2(ACLY).tif]

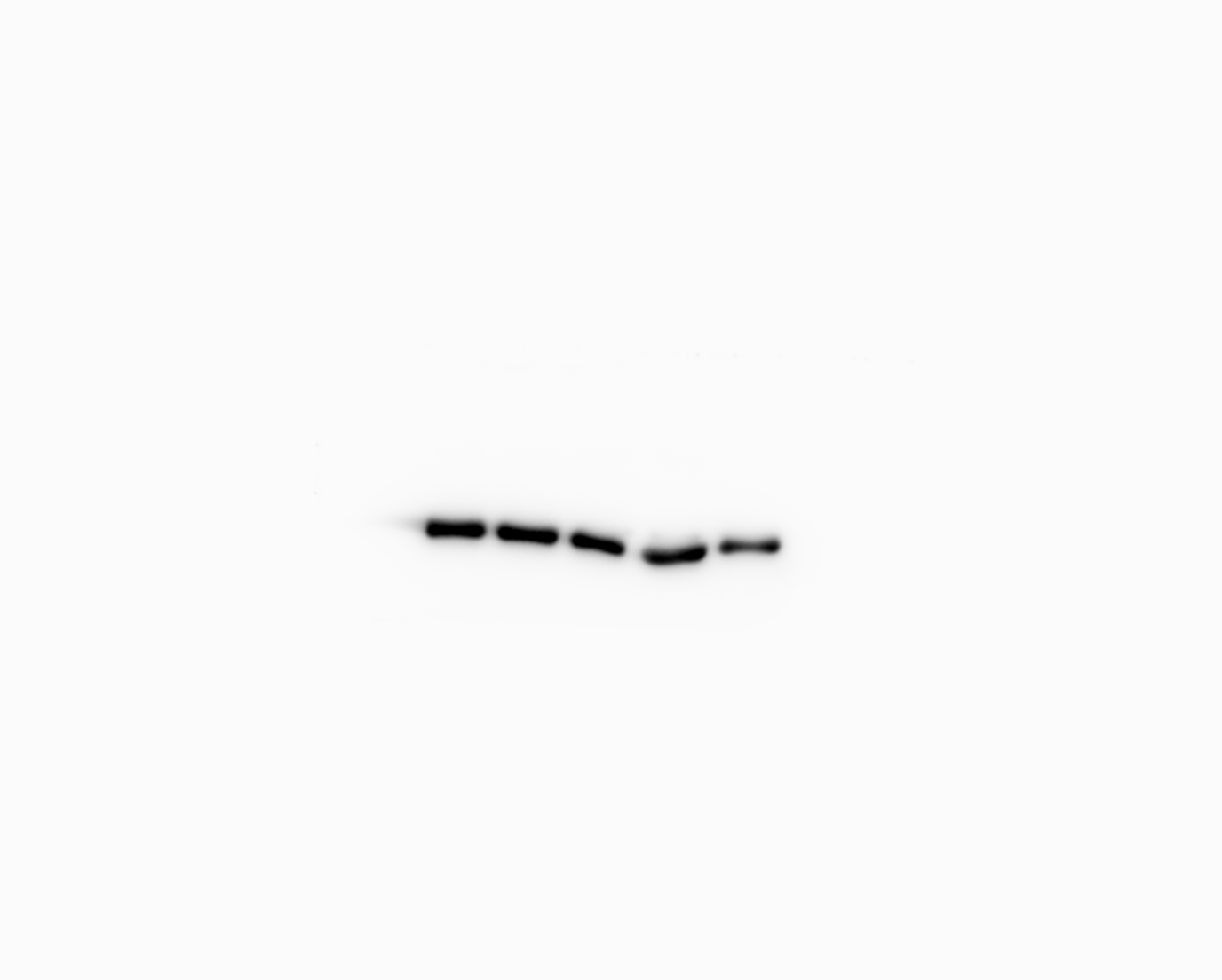

Supplement: Source data 1. [file elife-62394-data1.zip › Source data files/Source data (Raw)/Figure 3E-Source data-3(Actin).Tif]

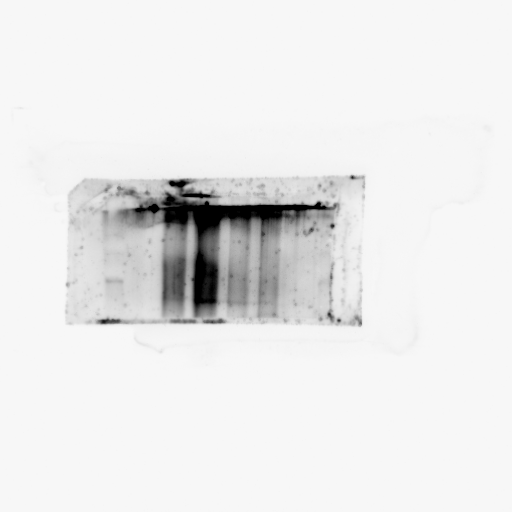

Supplement: Source data 1. [file elife-62394-data1.zip › Source data files/Source data (Raw)/Figure 3F-Source data-1(ACLY-(Ub)n).tif]

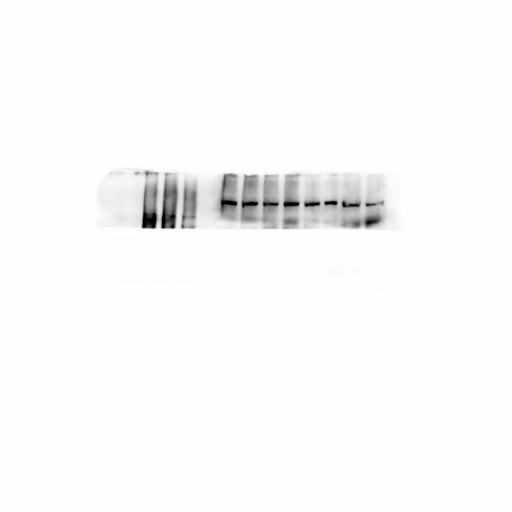

Supplement: Source data 1. [file elife-62394-data1.zip › Source data files/Source data (Raw)/Figure 3F-Source data-2(GFP).tif]

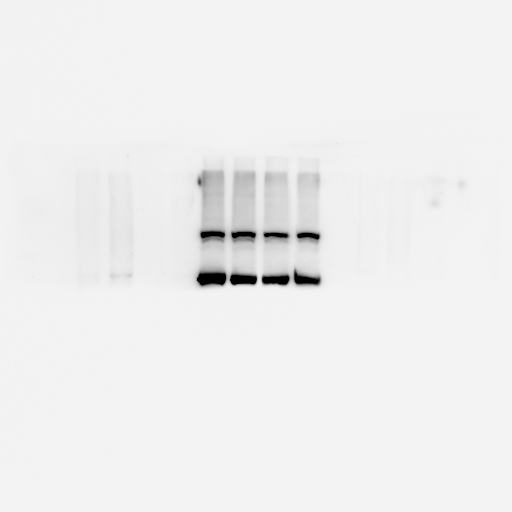

Supplement: Source data 1. [file elife-62394-data1.zip › Source data files/Source data (Raw)/Figure 3F-Source data-3(GFP).tif]

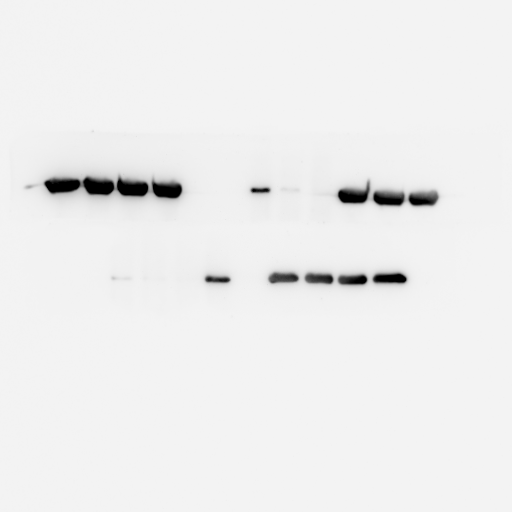

Supplement: Source data 1. [file elife-62394-data1.zip › Source data files/Source data (Raw)/Figure 3F-Source data-4(Actin).tif]

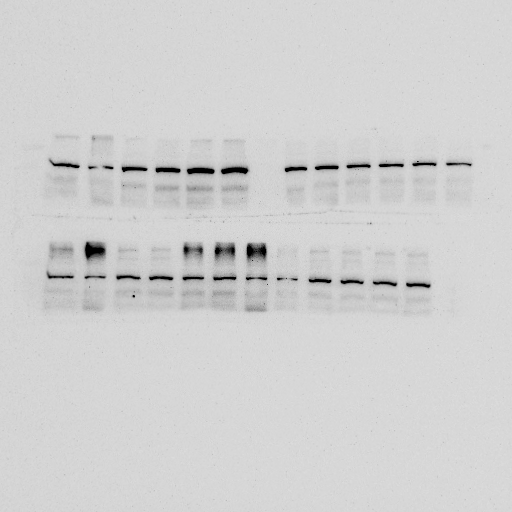

Supplement: Source data 1. [file elife-62394-data1.zip › Source data files/Source data (Raw)/Figure 3G-Source data-1(GFP).tif]

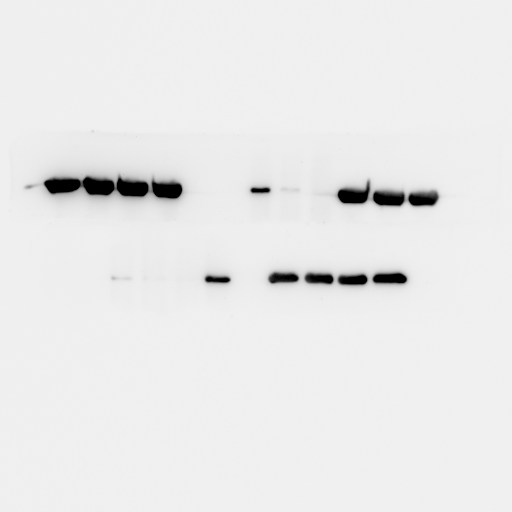

Supplement: Source data 1. [file elife-62394-data1.zip › Source data files/Source data (Raw)/Figure 3G-Source data-2(Actin).tif]

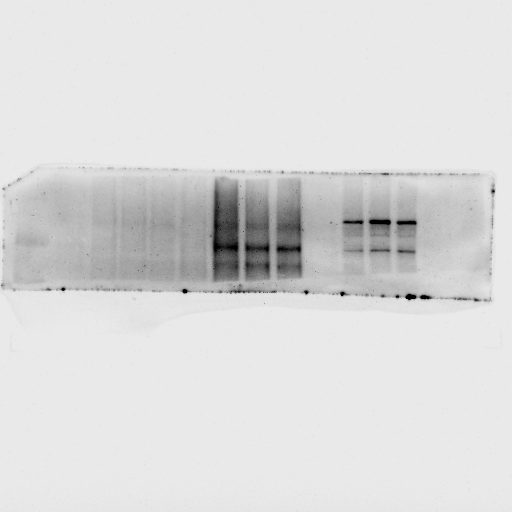

Supplement: Source data 1. [file elife-62394-data1.zip › Source data files/Source data (Raw)/Figure 4-figure supplement 1A-Source data-1 (USP13).tif]

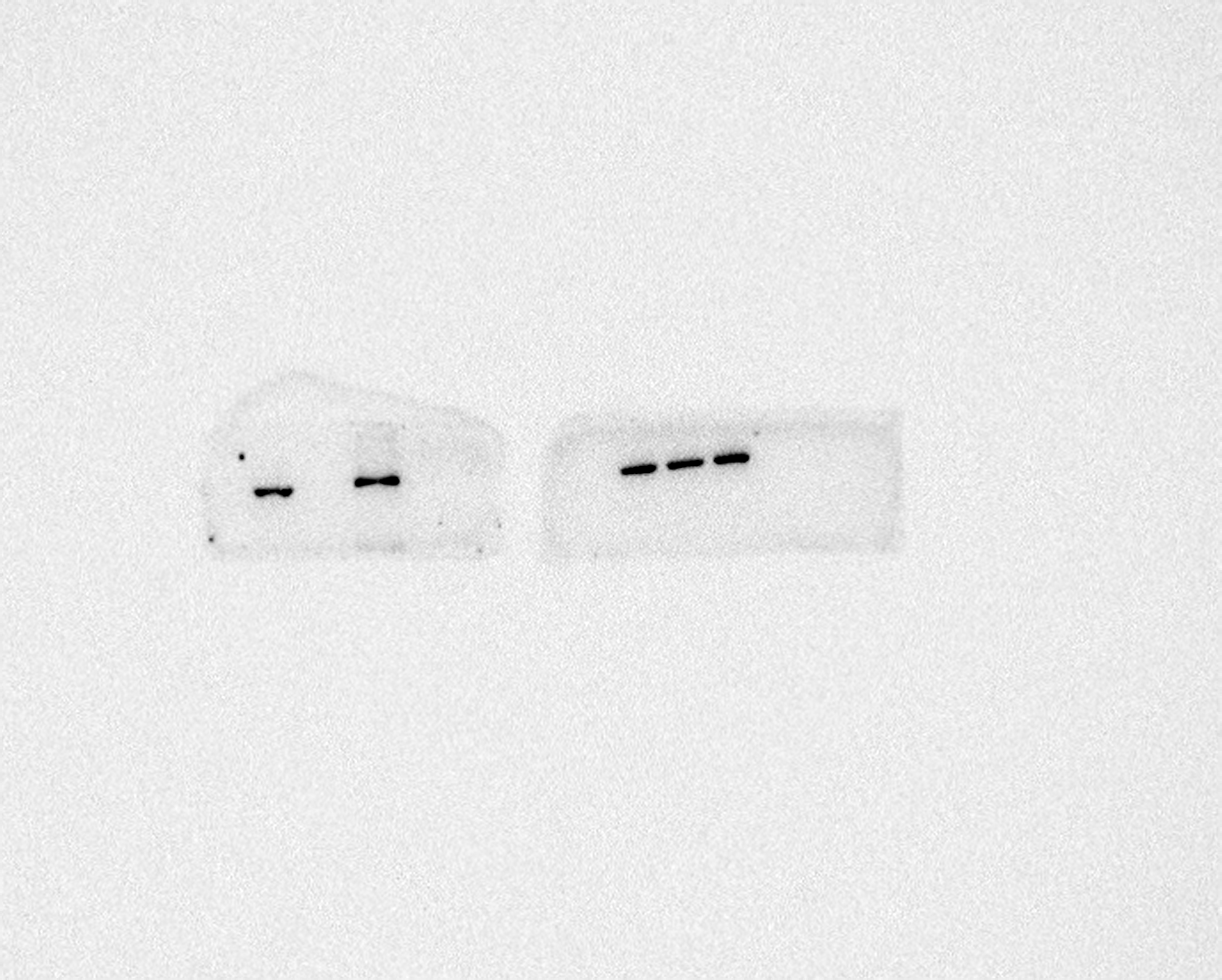

Supplement: Source data 1. [file elife-62394-data1.zip › Source data files/Source data (Raw)/Figure 4-figure supplement 1A-Source data-2 (CUL3).Tif]

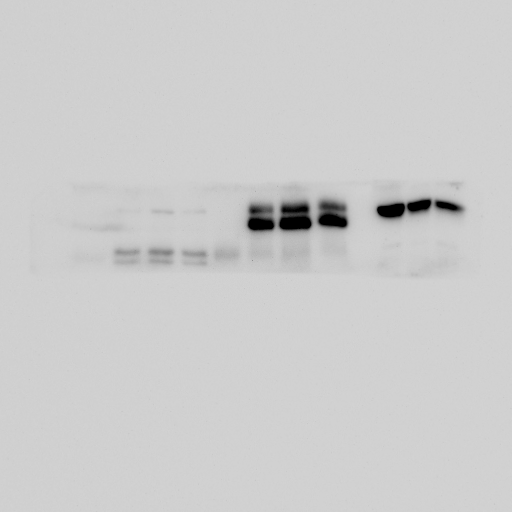

Supplement: Source data 1. [file elife-62394-data1.zip › Source data files/Source data (Raw)/Figure 4-figure supplement 1A-Source data-3 (KLHL25).tif]

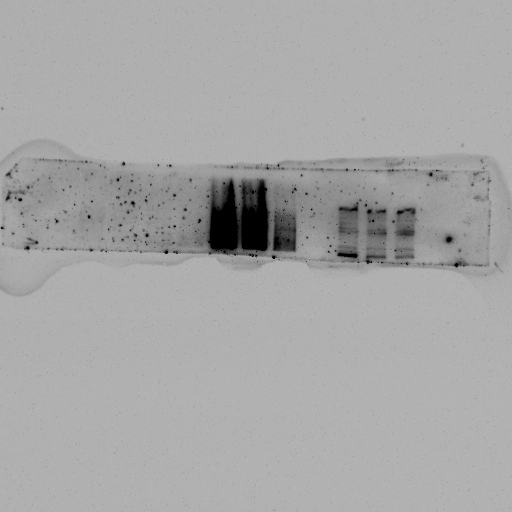

Supplement: Source data 1. [file elife-62394-data1.zip › Source data files/Source data (Raw)/Figure 4-figure supplement 1A-Source data-4 (UBR4).tif]

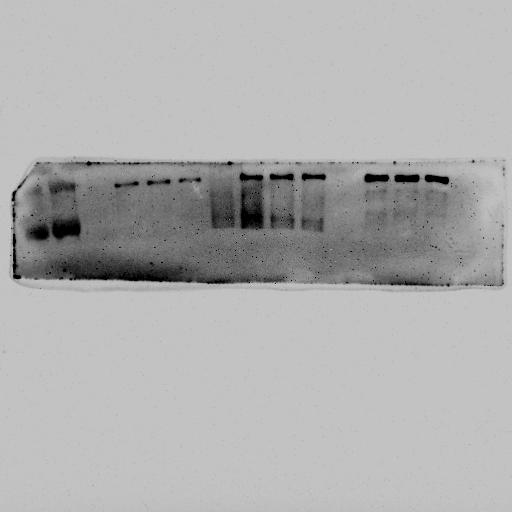

Supplement: Source data 1. [file elife-62394-data1.zip › Source data files/Source data (Raw)/Figure 4-figure supplement 1A-Source data-5 (ACLY).tif]

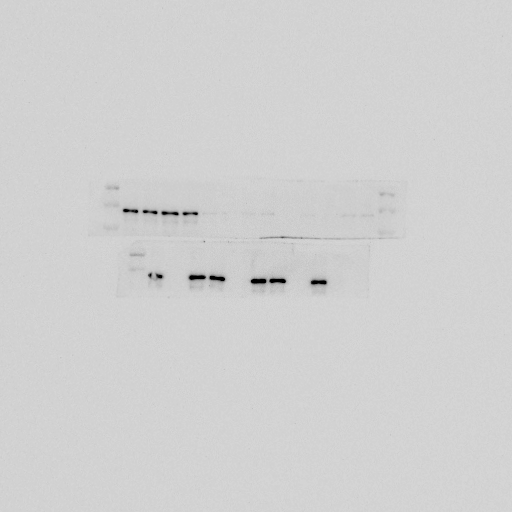

Supplement: Source data 1. [file elife-62394-data1.zip › Source data files/Source data (Raw)/Figure 4-figure supplement 1B-Source data-1 (ACLY).tif]

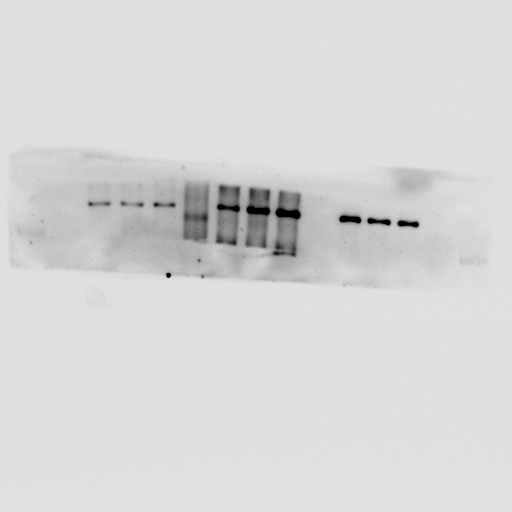

Supplement: Source data 1. [file elife-62394-data1.zip › Source data files/Source data (Raw)/Figure 4-figure supplement 1B-Source data-2 (USP13).tif]

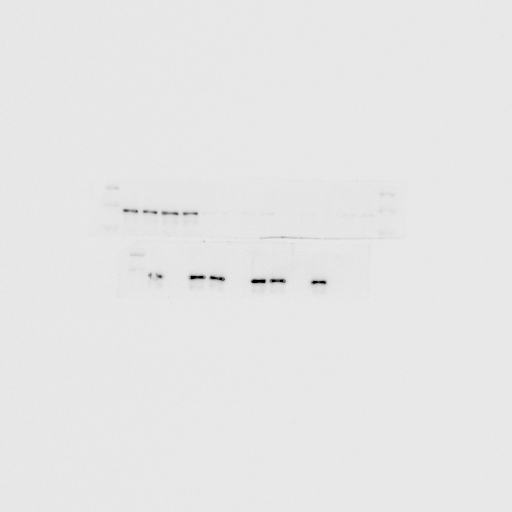

Supplement: Source data 1. [file elife-62394-data1.zip › Source data files/Source data (Raw)/Figure 4-figure supplement 1B-Source data-3 (ACLY).tif]

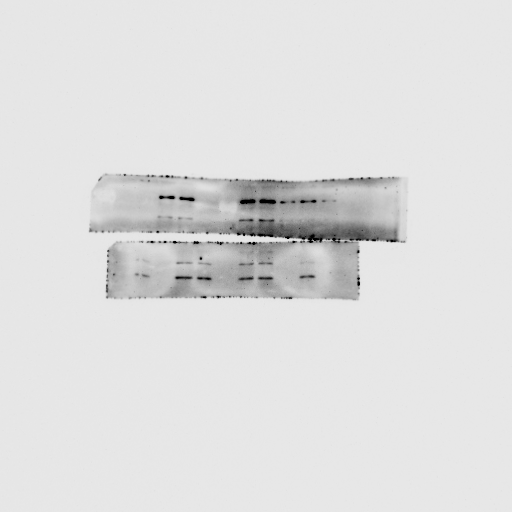

Supplement: Source data 1. [file elife-62394-data1.zip › Source data files/Source data (Raw)/Figure 4-figure supplement 1B-Source data-4 (KLHL25).tif]

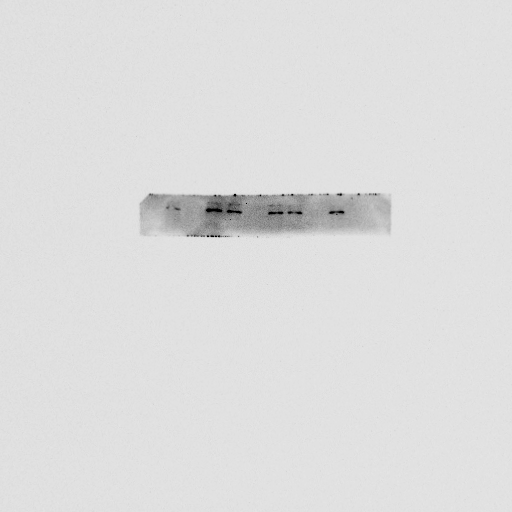

Supplement: Source data 1. [file elife-62394-data1.zip › Source data files/Source data (Raw)/Figure 4-figure supplement 1B-Source data-6 (CUL3).tif]

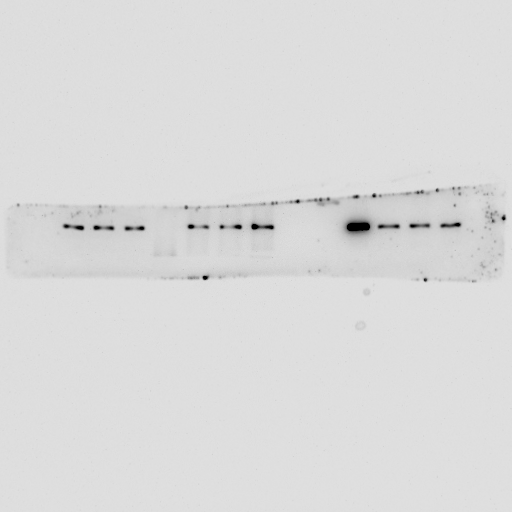

Supplement: Source data 1. [file elife-62394-data1.zip › Source data files/Source data (Raw)/Figure 4-figure supplement 1B-Source data-7 (ACLY).tif]

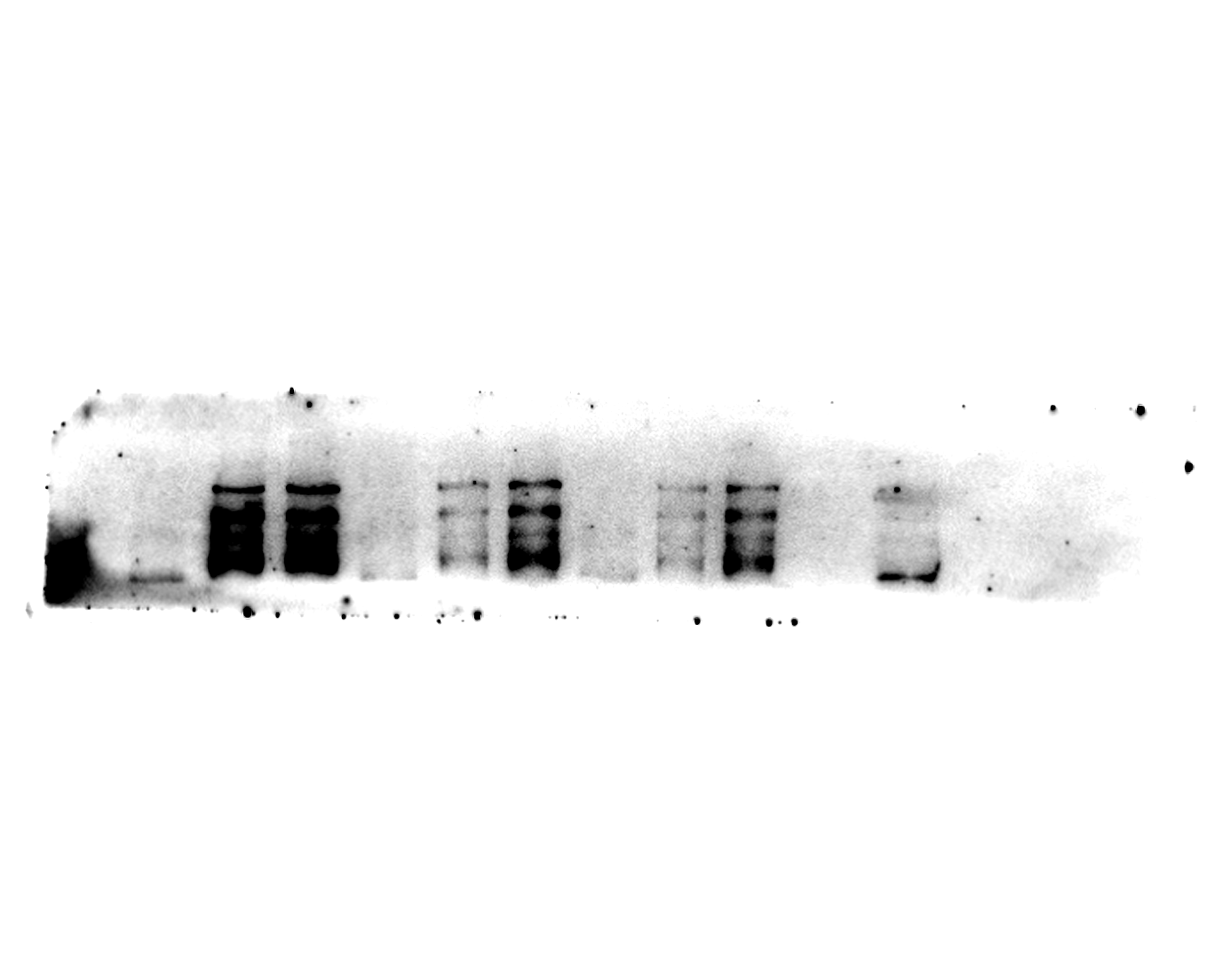

Supplement: Source data 1. [file elife-62394-data1.zip › Source data files/Source data (Raw)/Figure 4-figure supplement 1B-Source data-8 (UBR4).Tif]

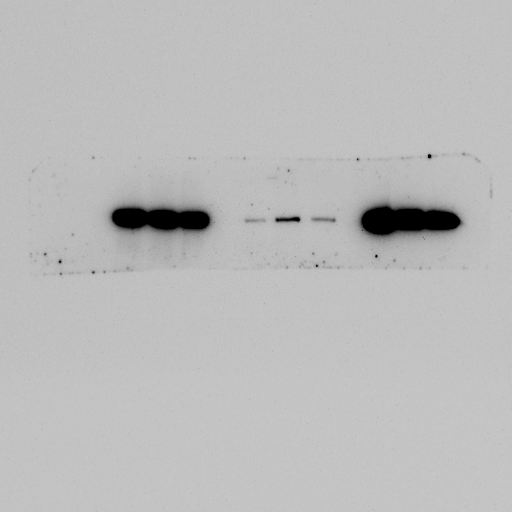

Supplement: Source data 1. [file elife-62394-data1.zip › Source data files/Source data (Raw)/Figure 4-figure supplement 1C-Source data-1 (ACLY).tif]

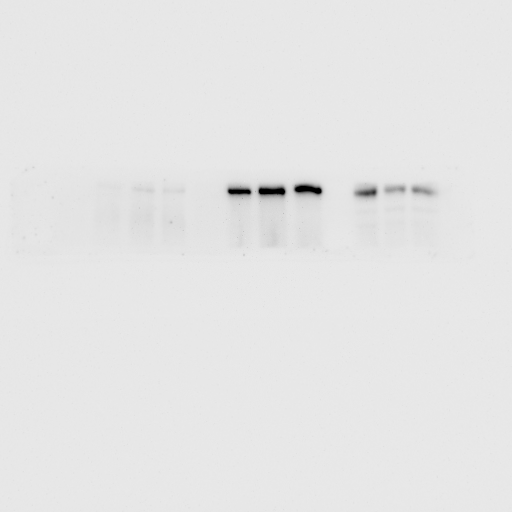

Supplement: Source data 1. [file elife-62394-data1.zip › Source data files/Source data (Raw)/Figure 4-figure supplement 1C-Source data-2 (CUL3).tif]

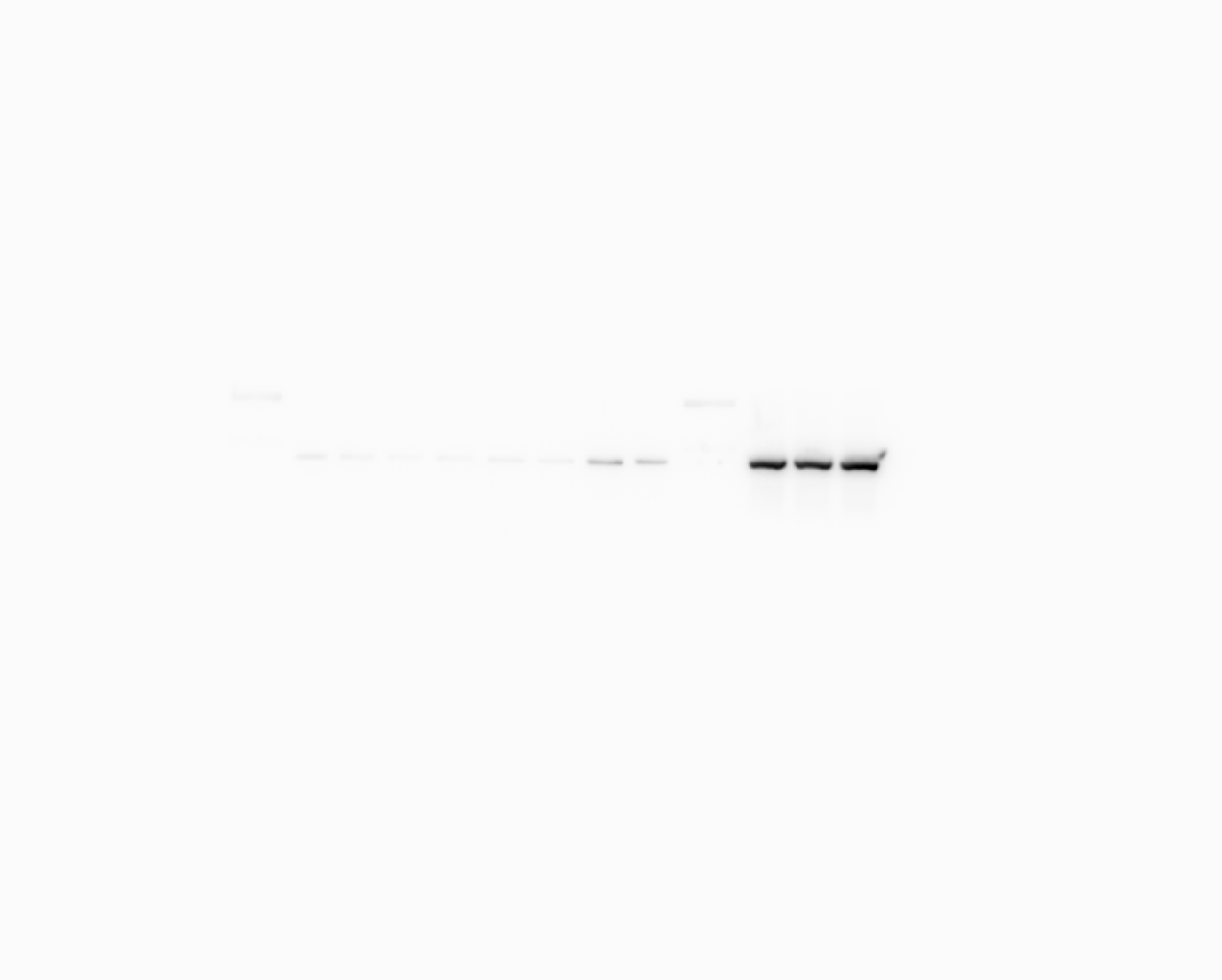

Supplement: Source data 1. [file elife-62394-data1.zip › Source data files/Source data (Raw)/Figure 4-figure supplement 1C-Source data-3 (ACLY).Tif]

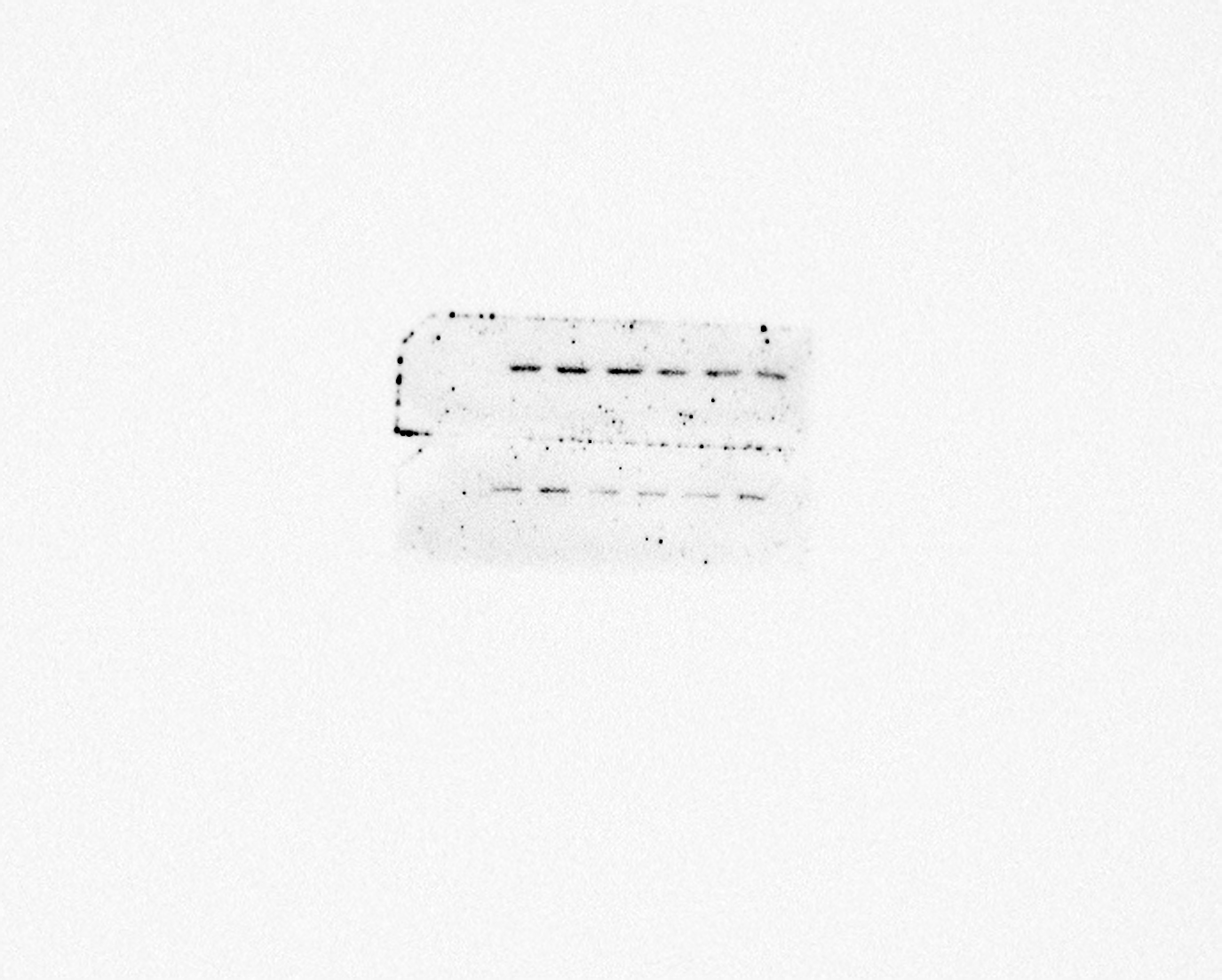

Supplement: Source data 1. [file elife-62394-data1.zip › Source data files/Source data (Raw)/Figure 4-figure supplement 2A-Source data-1 (KLHL25).tif]

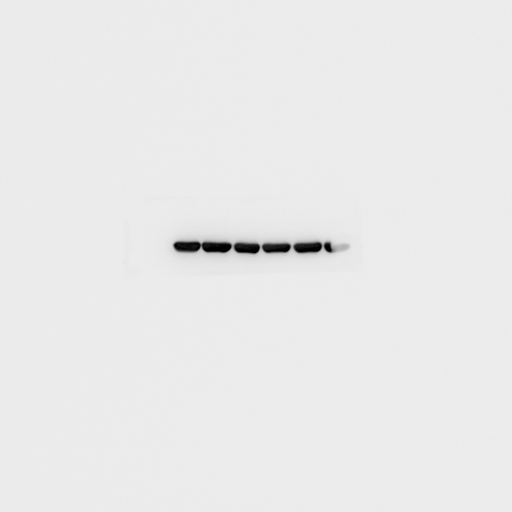

Supplement: Source data 1. [file elife-62394-data1.zip › Source data files/Source data (Raw)/Figure 4-figure supplement 2A-Source data-2 (Actin).tif]

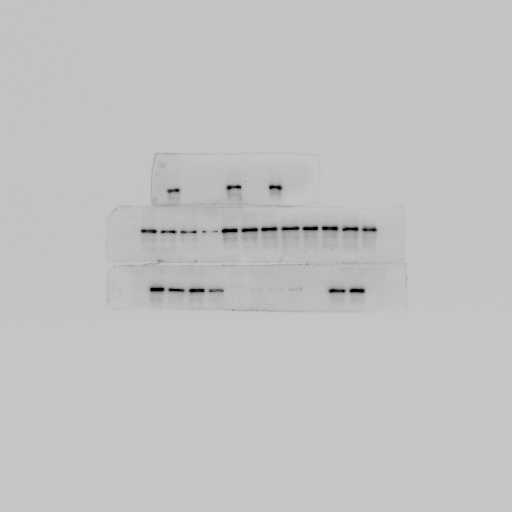

Supplement: Source data 1. [file elife-62394-data1.zip › Source data files/Source data (Raw)/Figure 4-figure supplement 2B-Source data-1 (ACLY).tif]

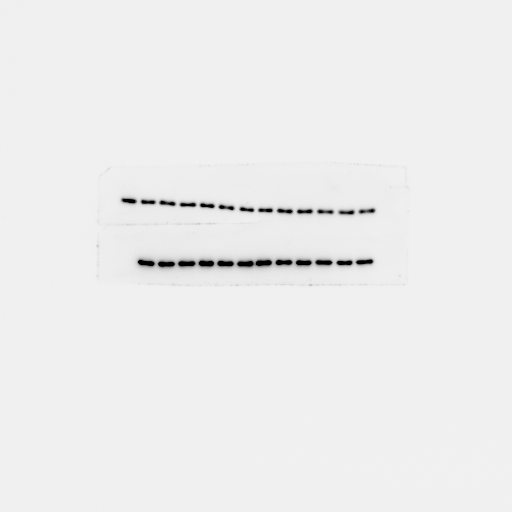

Supplement: Source data 1. [file elife-62394-data1.zip › Source data files/Source data (Raw)/Figure 4-figure supplement 2B-Source data-2(Actin).tif]

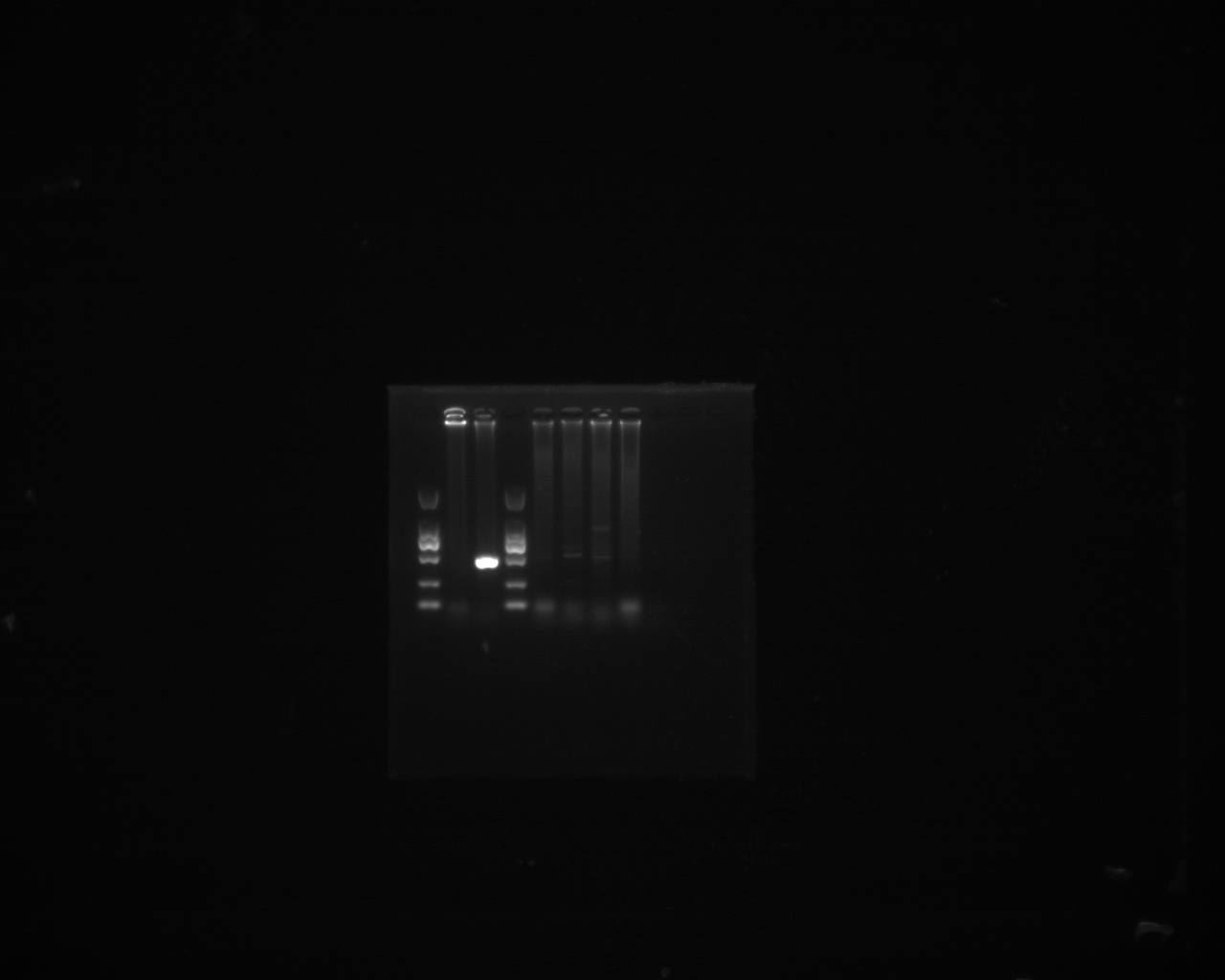

Supplement: Source data 1. [file elife-62394-data1.zip › Source data files/Source data (Raw)/Figure 4-figure supplement 3A-Source data.Tif]

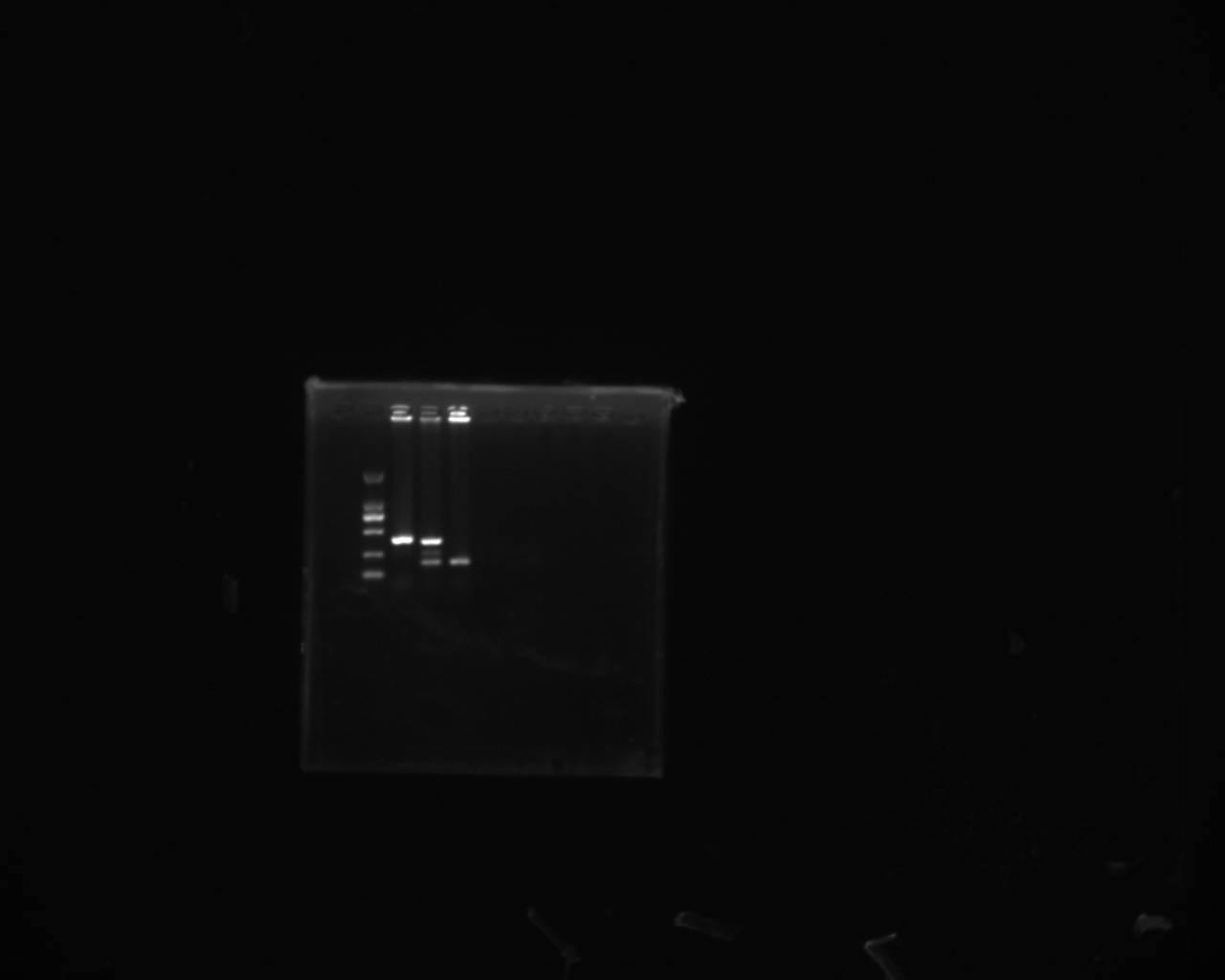

Supplement: Source data 1. [file elife-62394-data1.zip › Source data files/Source data (Raw)/Figure 4-figure supplement 3B-Source data.Tif]

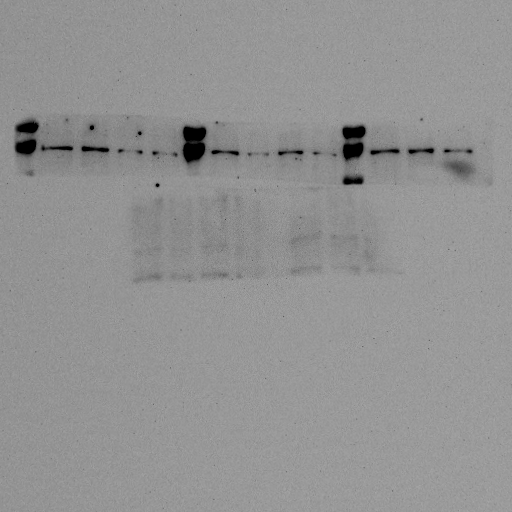

Supplement: Source data 1. [file elife-62394-data1.zip › Source data files/Source data (Raw)/Figure 4-figure supplement 4B-Source data-1 (ACLY).tif]

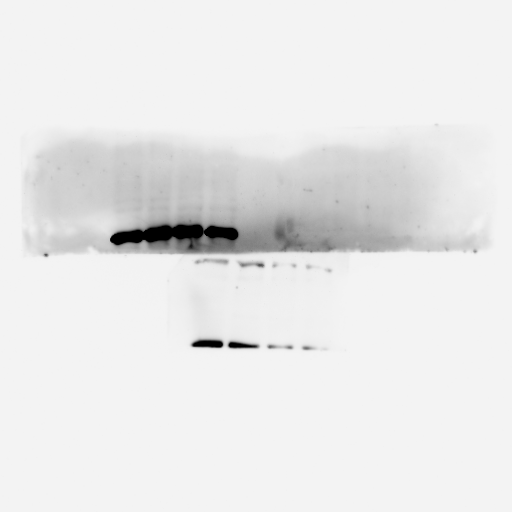

Supplement: Source data 1. [file elife-62394-data1.zip › Source data files/Source data (Raw)/Figure 4-figure supplement 4B-Source data-2 (CUL3).tif]

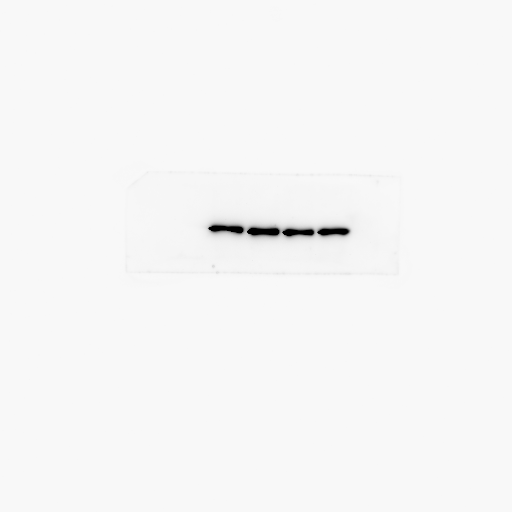

Supplement: Source data 1. [file elife-62394-data1.zip › Source data files/Source data (Raw)/Figure 4-figure supplement 4B-Source data-3 (Actin).tif]

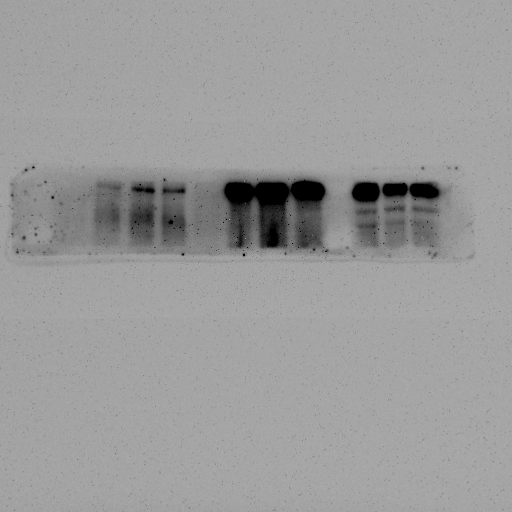

Supplement: Source data 1. [file elife-62394-data1.zip › Source data files/Source data (Raw)/Figure 4A-Source data-1 (CUL3).tif]

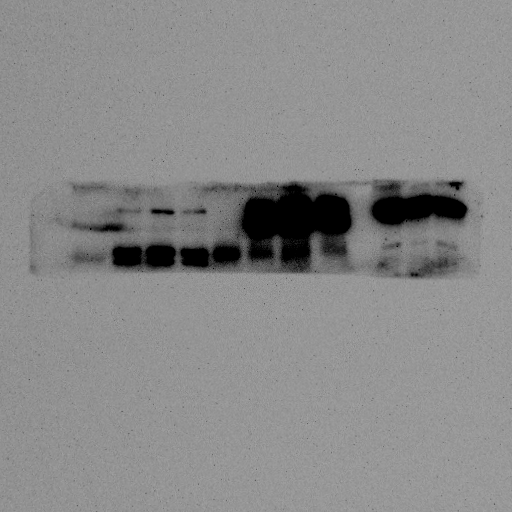

Supplement: Source data 1. [file elife-62394-data1.zip › Source data files/Source data (Raw)/Figure 4A-Source data-2 (KLHL25).tif]

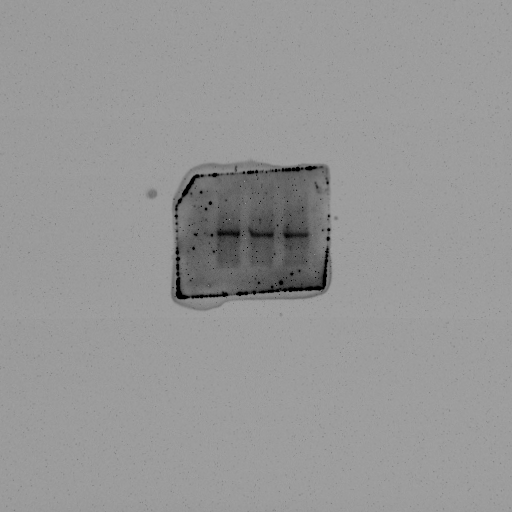

Supplement: Source data 1. [file elife-62394-data1.zip › Source data files/Source data (Raw)/Figure 4A-Source data-3 (USP13).tif]

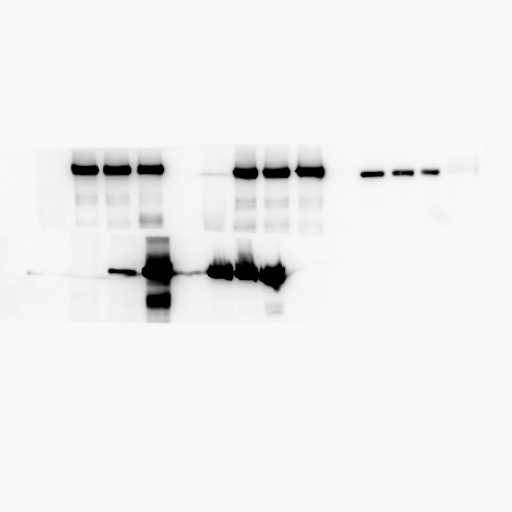

Supplement: Source data 1. [file elife-62394-data1.zip › Source data files/Source data (Raw)/Figure 4A-Source data-4 (ACLY).tif]

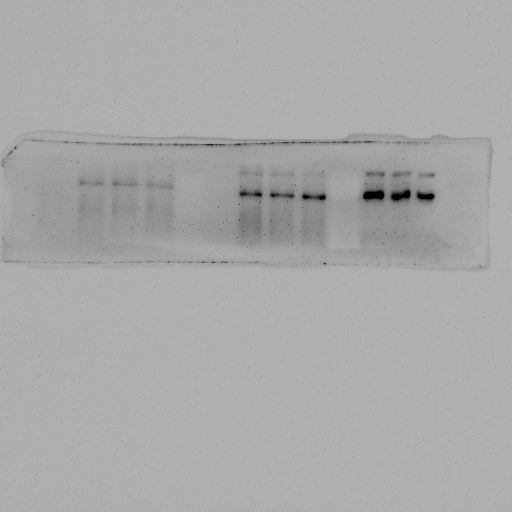

Supplement: Source data 1. [file elife-62394-data1.zip › Source data files/Source data (Raw)/Figure 4A-Source data-5 (CUL3).tif]

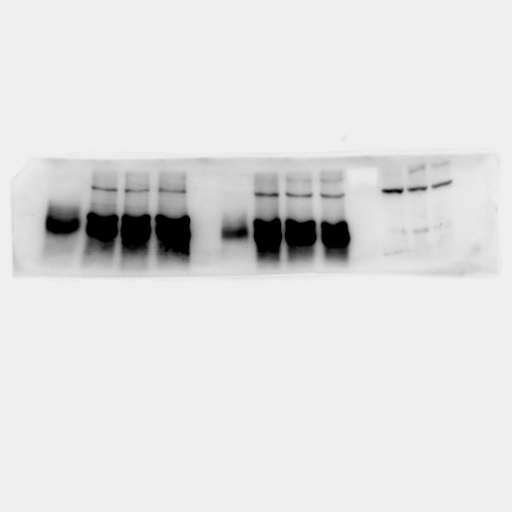

Supplement: Source data 1. [file elife-62394-data1.zip › Source data files/Source data (Raw)/Figure 4A-Source data-6 (KLHL25).tif]

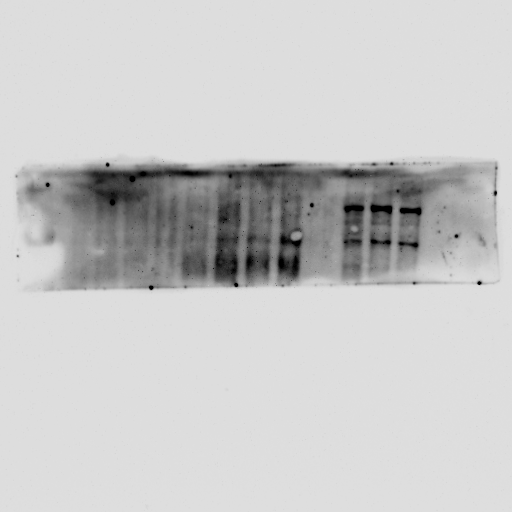

Supplement: Source data 1. [file elife-62394-data1.zip › Source data files/Source data (Raw)/Figure 4A-Source data-7 (USP13).tif]

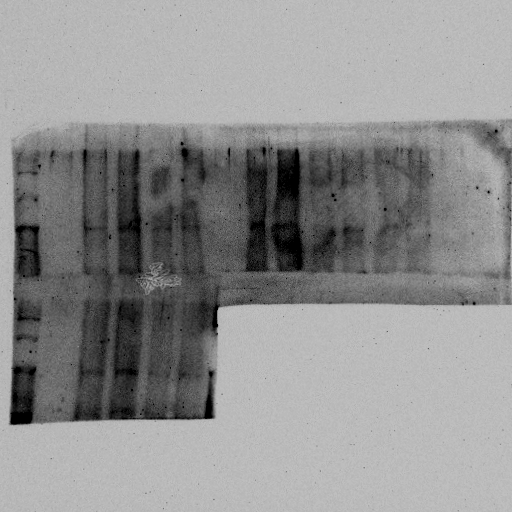

Supplement: Source data 1. [file elife-62394-data1.zip › Source data files/Source data (Raw)/Figure 4B-Source data-1 (ACLY-(Ub)n).tif]

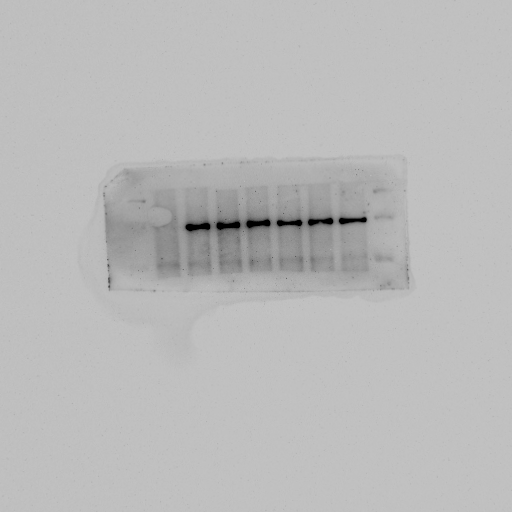

Supplement: Source data 1. [file elife-62394-data1.zip › Source data files/Source data (Raw)/Figure 4B-Source data-2 (ACLY).tif]

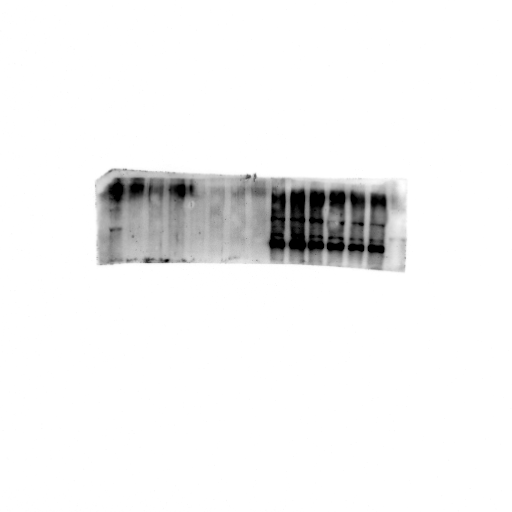

Supplement: Source data 1. [file elife-62394-data1.zip › Source data files/Source data (Raw)/Figure 4B-Source data-3 (Ub).tif]

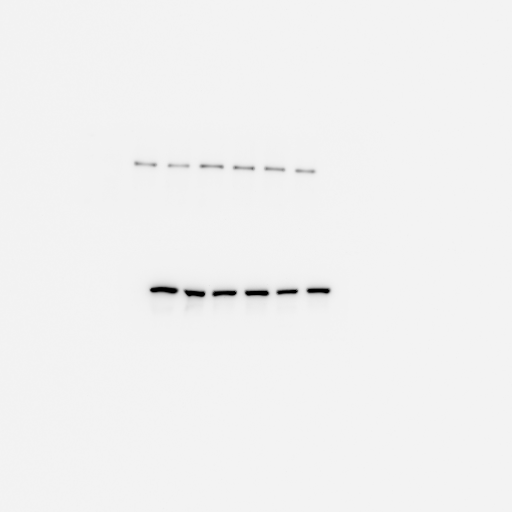

Supplement: Source data 1. [file elife-62394-data1.zip › Source data files/Source data (Raw)/Figure 4B-Source data-4 (ACLY).tif]

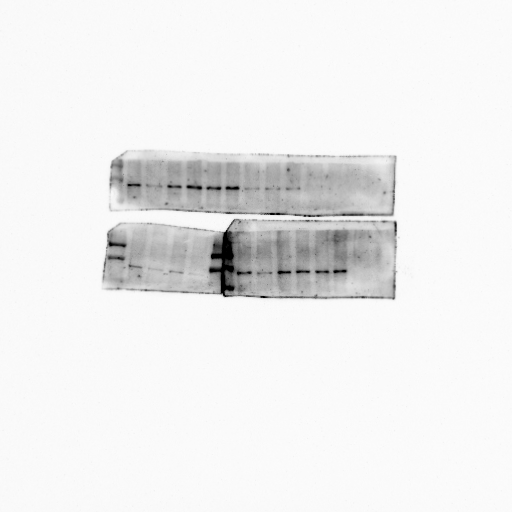

Supplement: Source data 1. [file elife-62394-data1.zip › Source data files/Source data (Raw)/Figure 4C-Source data-1 (ACLY).tif]

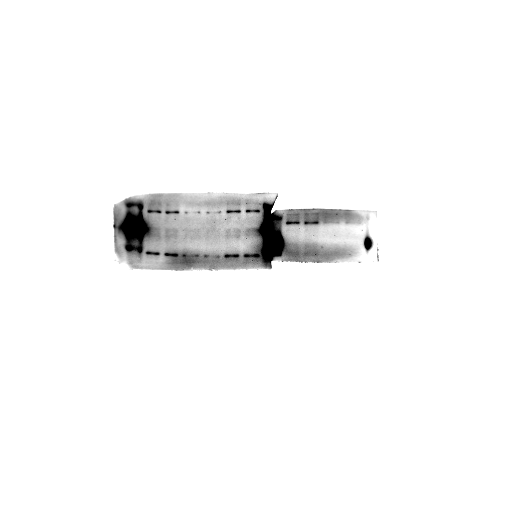

Supplement: Source data 1. [file elife-62394-data1.zip › Source data files/Source data (Raw)/Figure 4C-Source data-2 (Cul3).tif]

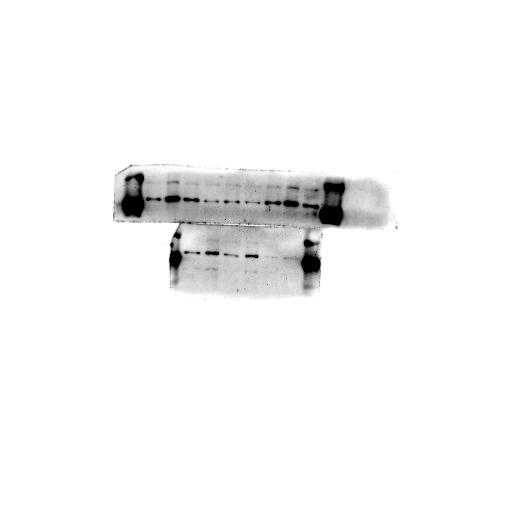

Supplement: Source data 1. [file elife-62394-data1.zip › Source data files/Source data (Raw)/Figure 4C-Source data-3 (KLHL25).tif]

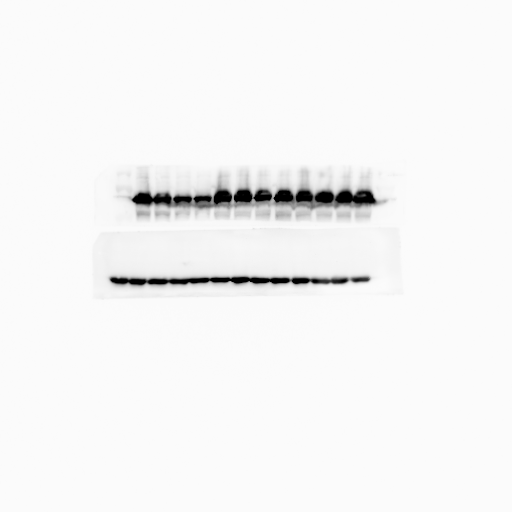

Supplement: Source data 1. [file elife-62394-data1.zip › Source data files/Source data (Raw)/Figure 4C-Source data-4 (Actin).tif]

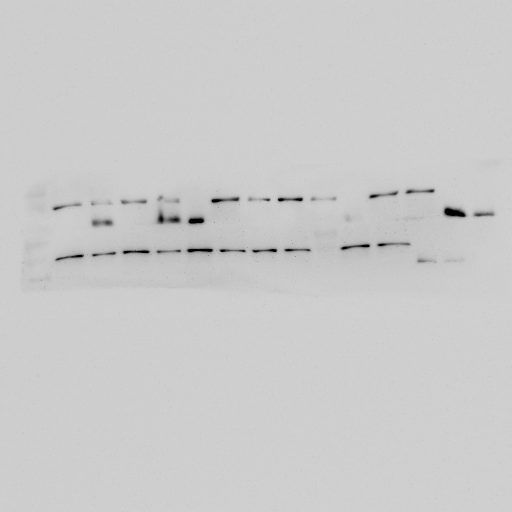

Supplement: Source data 1. [file elife-62394-data1.zip › Source data files/Source data (Raw)/Figure 4D-Source data-1(ACLY).tif]

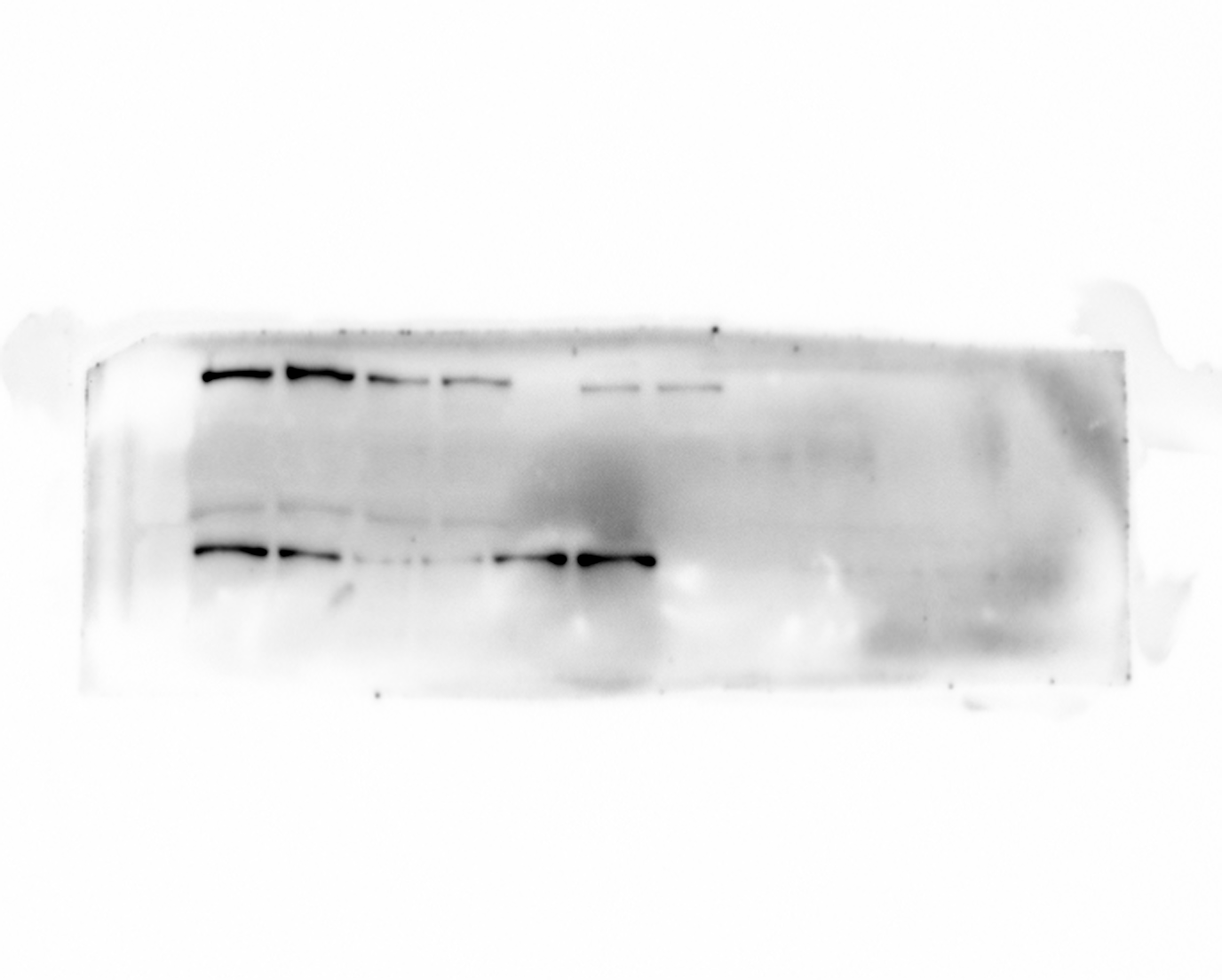

Supplement: Source data 1. [file elife-62394-data1.zip › Source data files/Source data (Raw)/Figure 4D-Source data-2(CUL3).Tif]

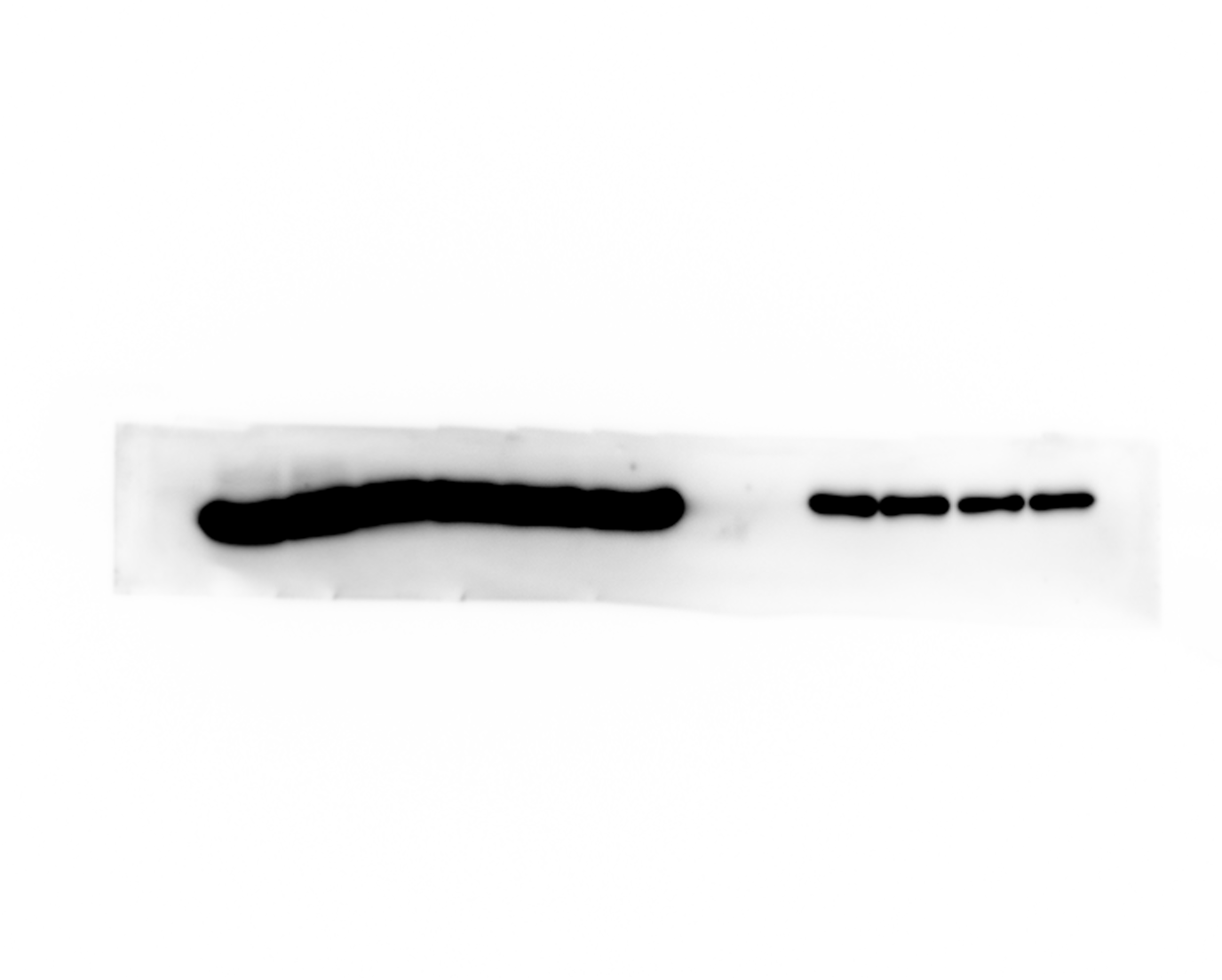

Supplement: Source data 1. [file elife-62394-data1.zip › Source data files/Source data (Raw)/Figure 4D-Source data-3(Actin).Tif]

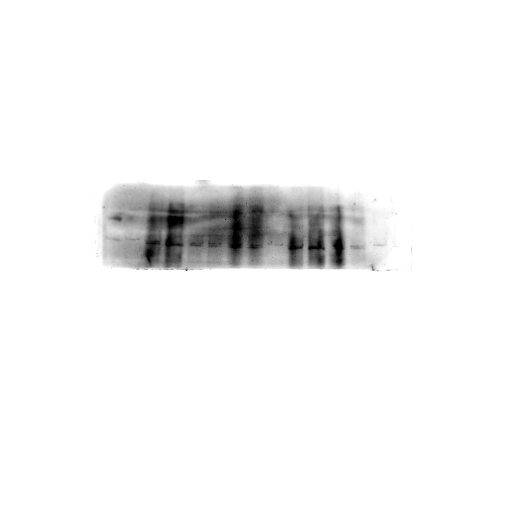

Supplement: Source data 1. [file elife-62394-data1.zip › Source data files/Source data (Raw)/Figure 4F-Source data-1(ACLY-(Ub)n).tif]

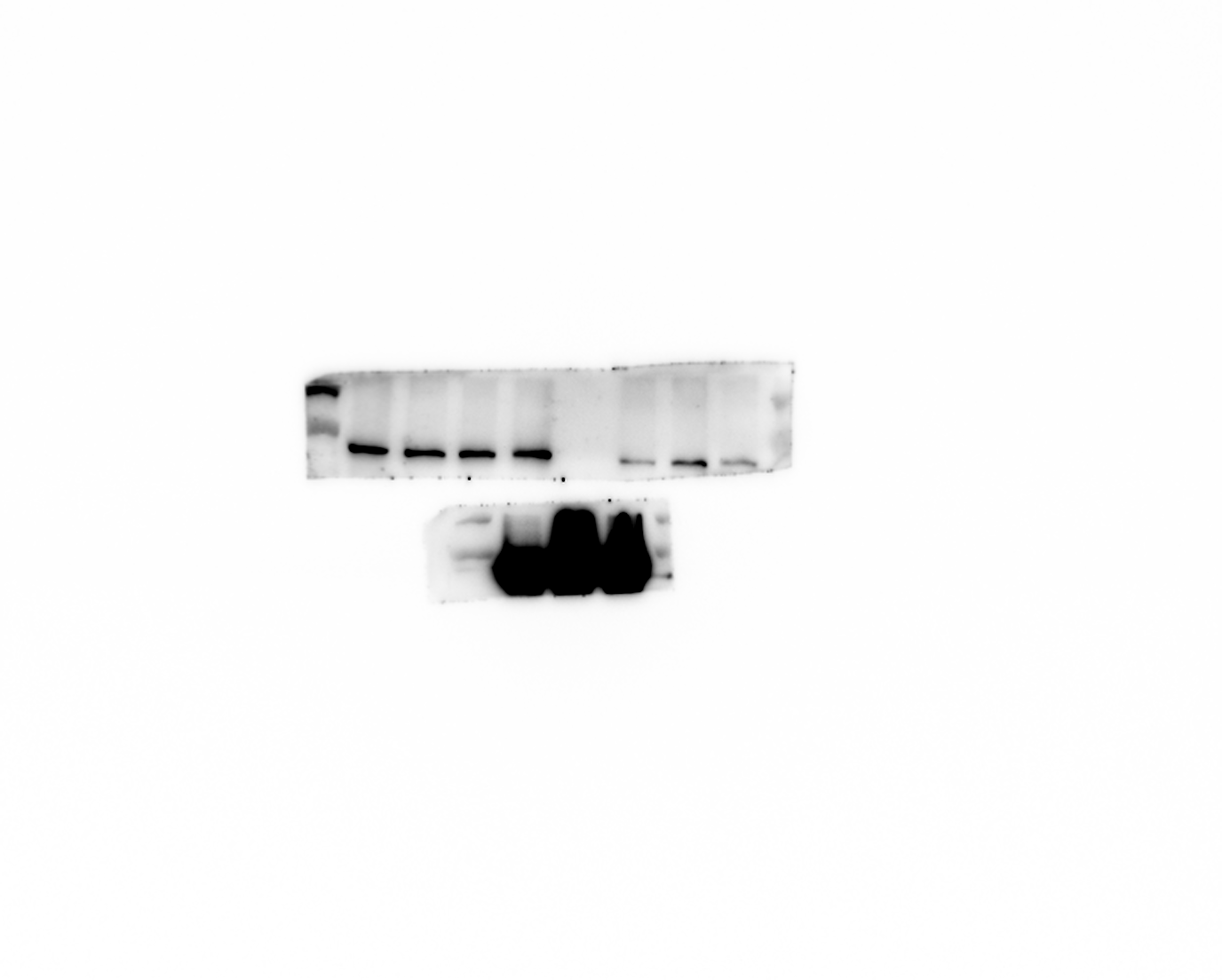

Supplement: Source data 1. [file elife-62394-data1.zip › Source data files/Source data (Raw)/Figure 4F-Source data-2(ACLY).Tif]

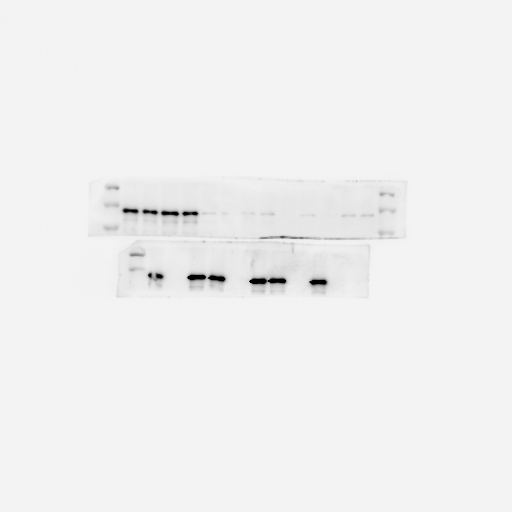

Supplement: Source data 1. [file elife-62394-data1.zip › Source data files/Source data (Raw)/Figure 4F-Source data-3(ACLY).tif]

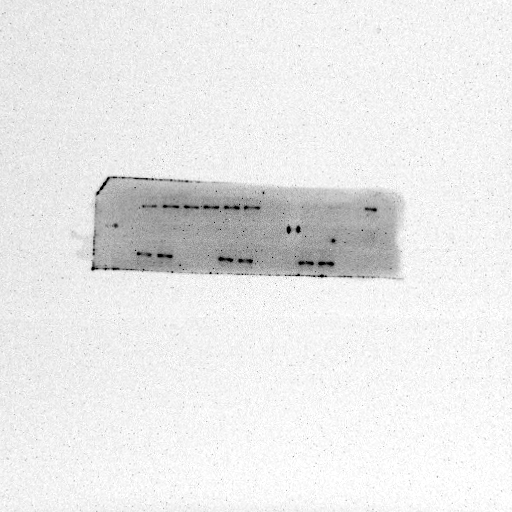

Supplement: Source data 1. [file elife-62394-data1.zip › Source data files/Source data (Raw)/Figure 4F-Source data-4(CUL3).tif]

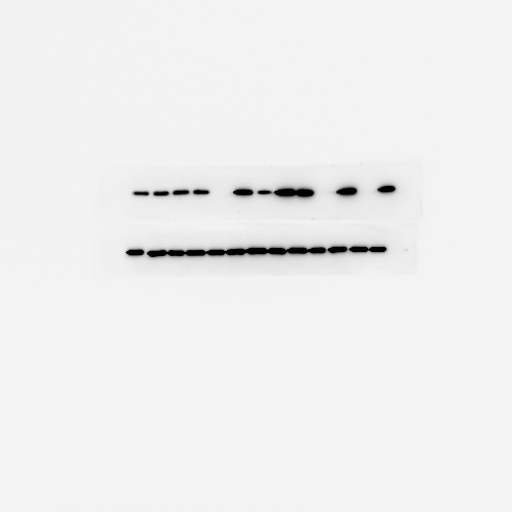

Supplement: Source data 1. [file elife-62394-data1.zip › Source data files/Source data (Raw)/Figure 4F-Source data-5(Actin).tif]

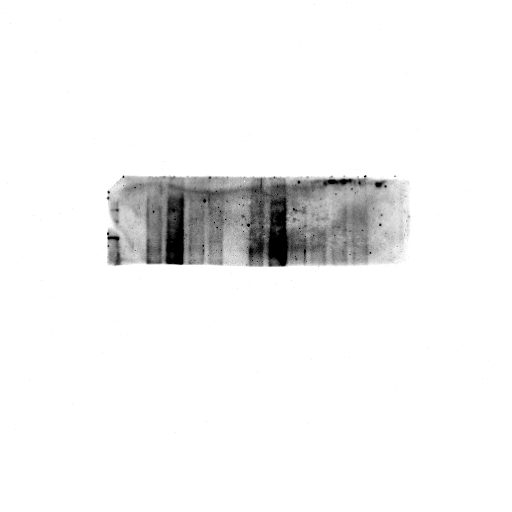

Supplement: Source data 1. [file elife-62394-data1.zip › Source data files/Source data (Raw)/Figure 4G-Source data-1(ACLY).tif]

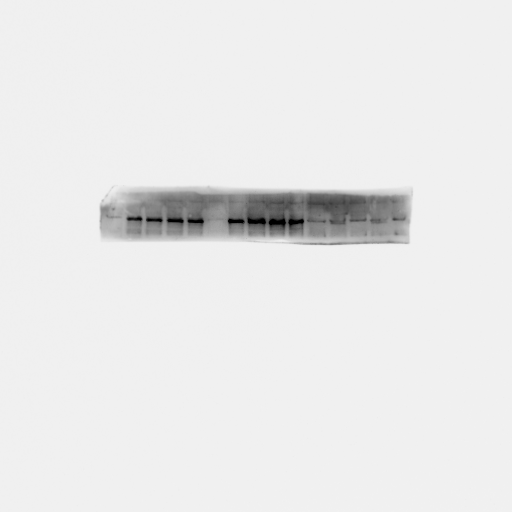

Supplement: Source data 1. [file elife-62394-data1.zip › Source data files/Source data (Raw)/Figure 4G-Source data-2(ACLY).tif]

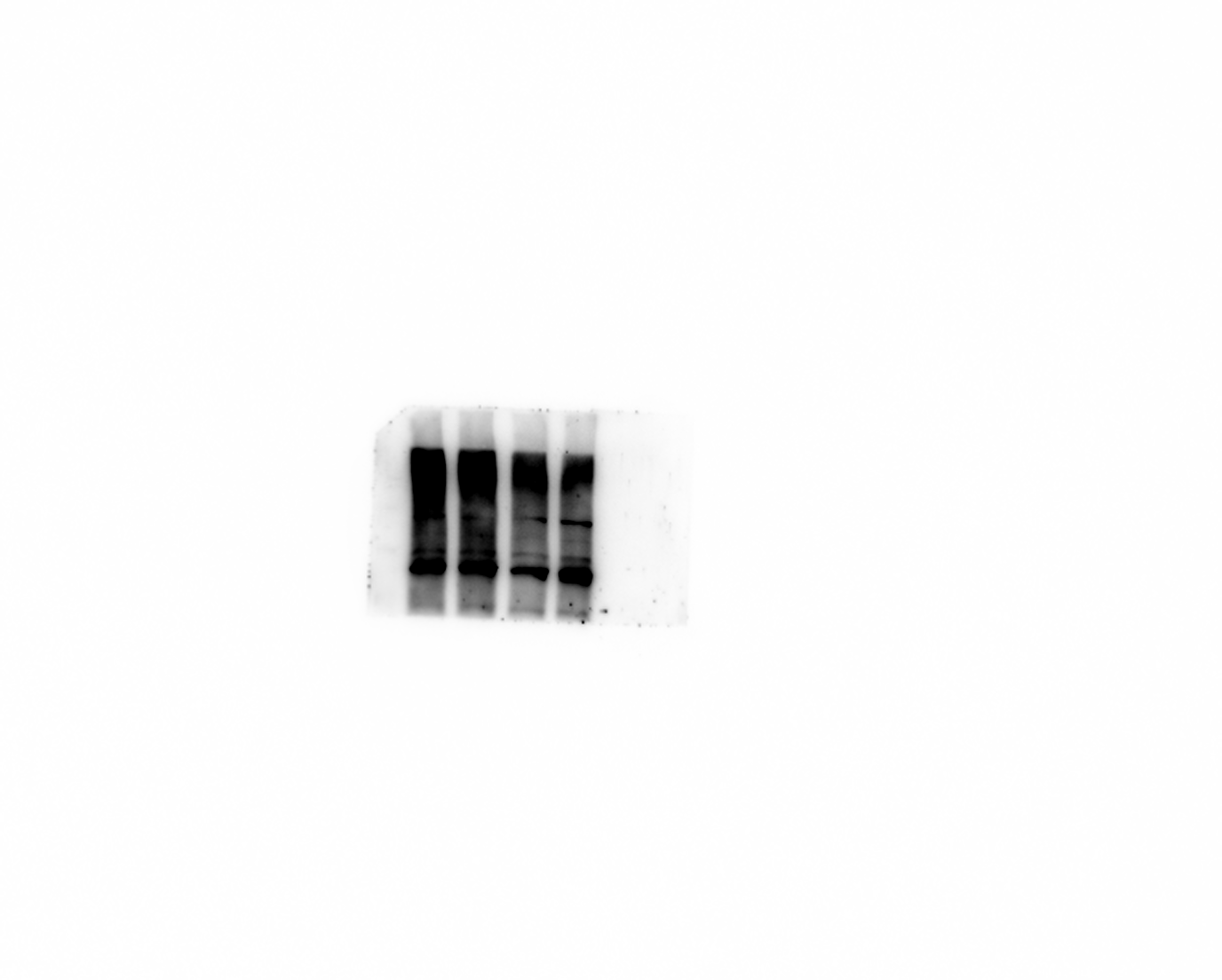

Supplement: Source data 1. [file elife-62394-data1.zip › Source data files/Source data (Raw)/Figure 4G-Source data-3 (Ub).Tif]

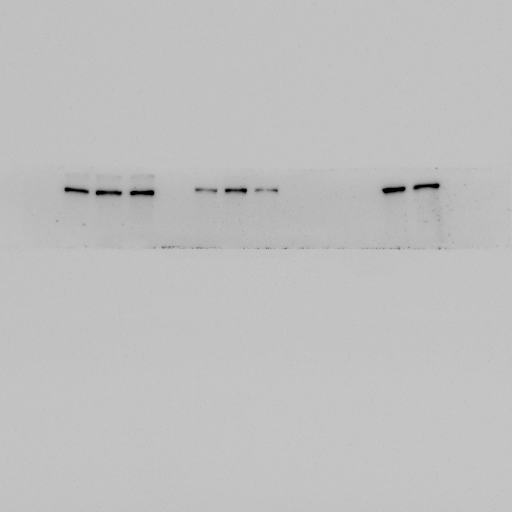

Supplement: Source data 1. [file elife-62394-data1.zip › Source data files/Source data (Raw)/Figure 4G-Source data-4 (Cul3).tif]

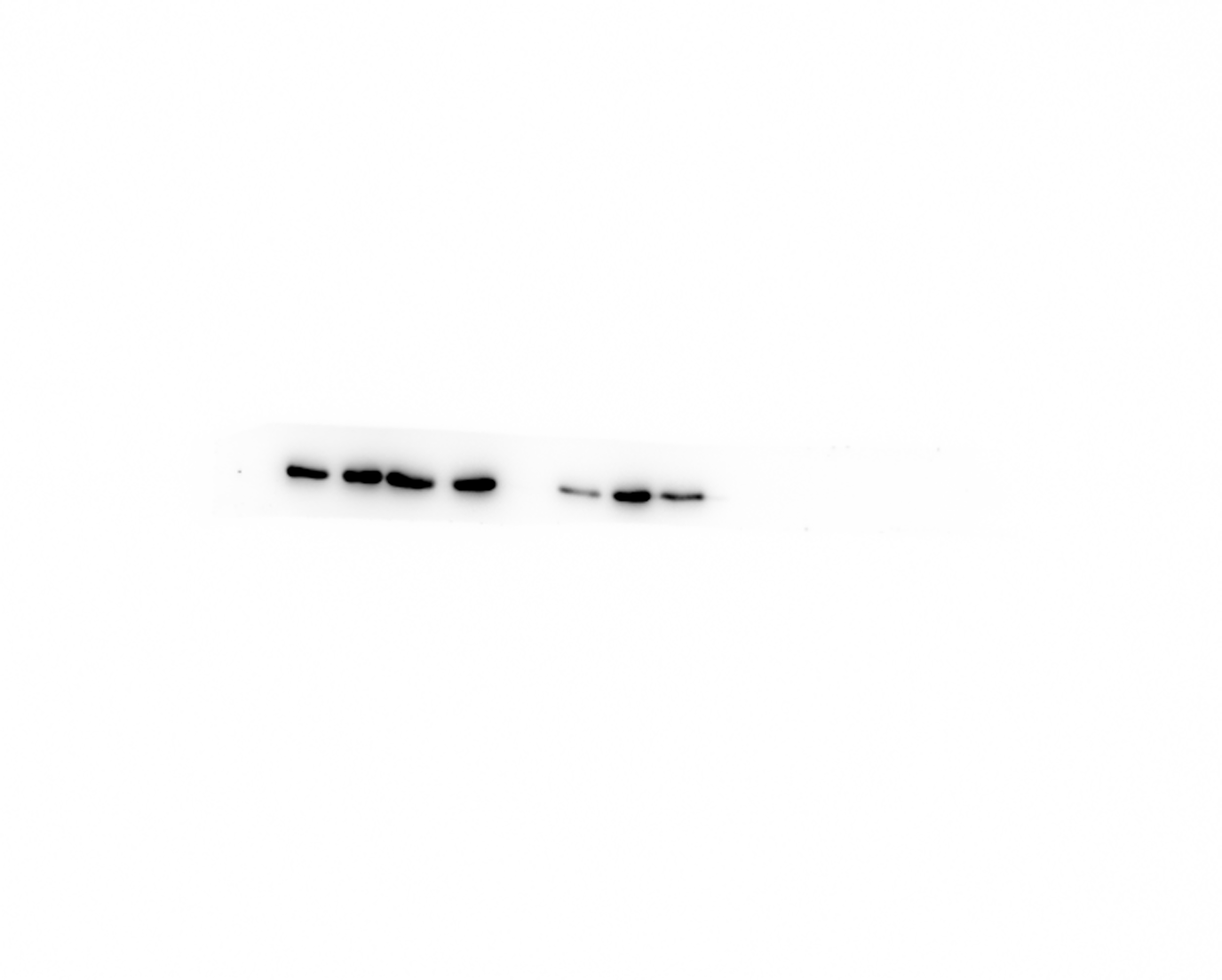

Supplement: Source data 1. [file elife-62394-data1.zip › Source data files/Source data (Raw)/Figure 4G-Source data-5 (Actin).Tif]

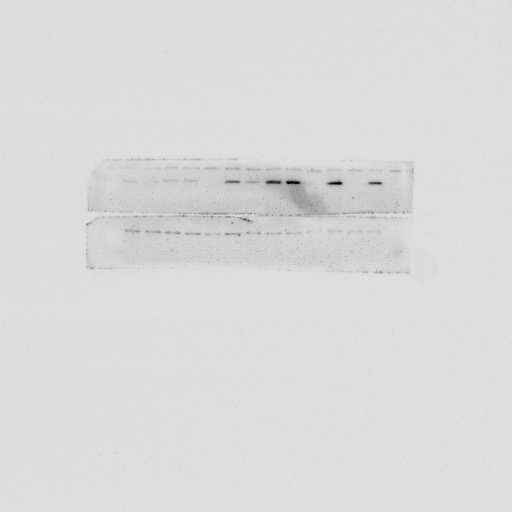

Supplement: Source data 1. [file elife-62394-data1.zip › Source data files/Source data (Raw)/Figure 4H-Source data-1 (ACLY).tif]

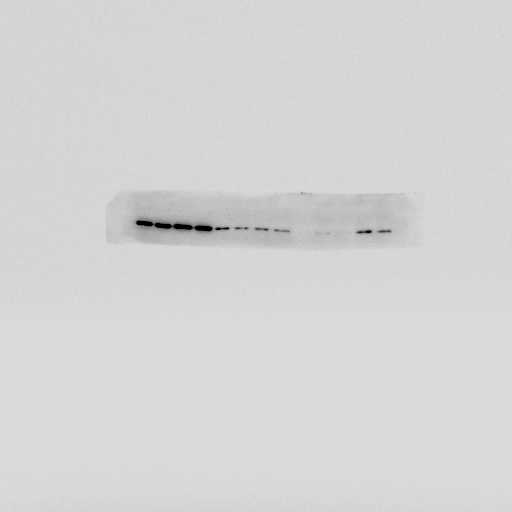

Supplement: Source data 1. [file elife-62394-data1.zip › Source data files/Source data (Raw)/Figure 4H-Source data-3 (Actin).tif]

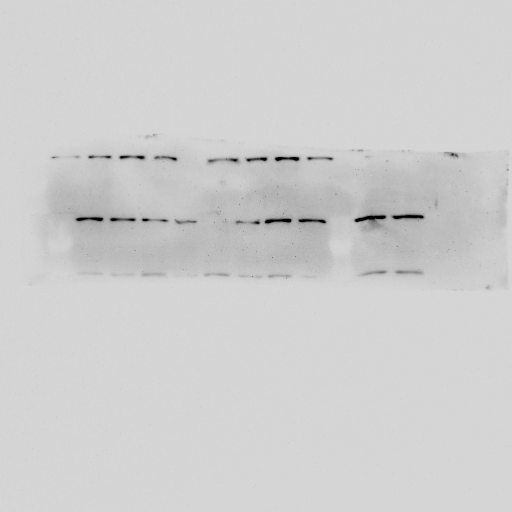

Supplement: Source data 1. [file elife-62394-data1.zip › Source data files/Source data (Raw)/Figure 4I-Source data-2(CUL3).tif]

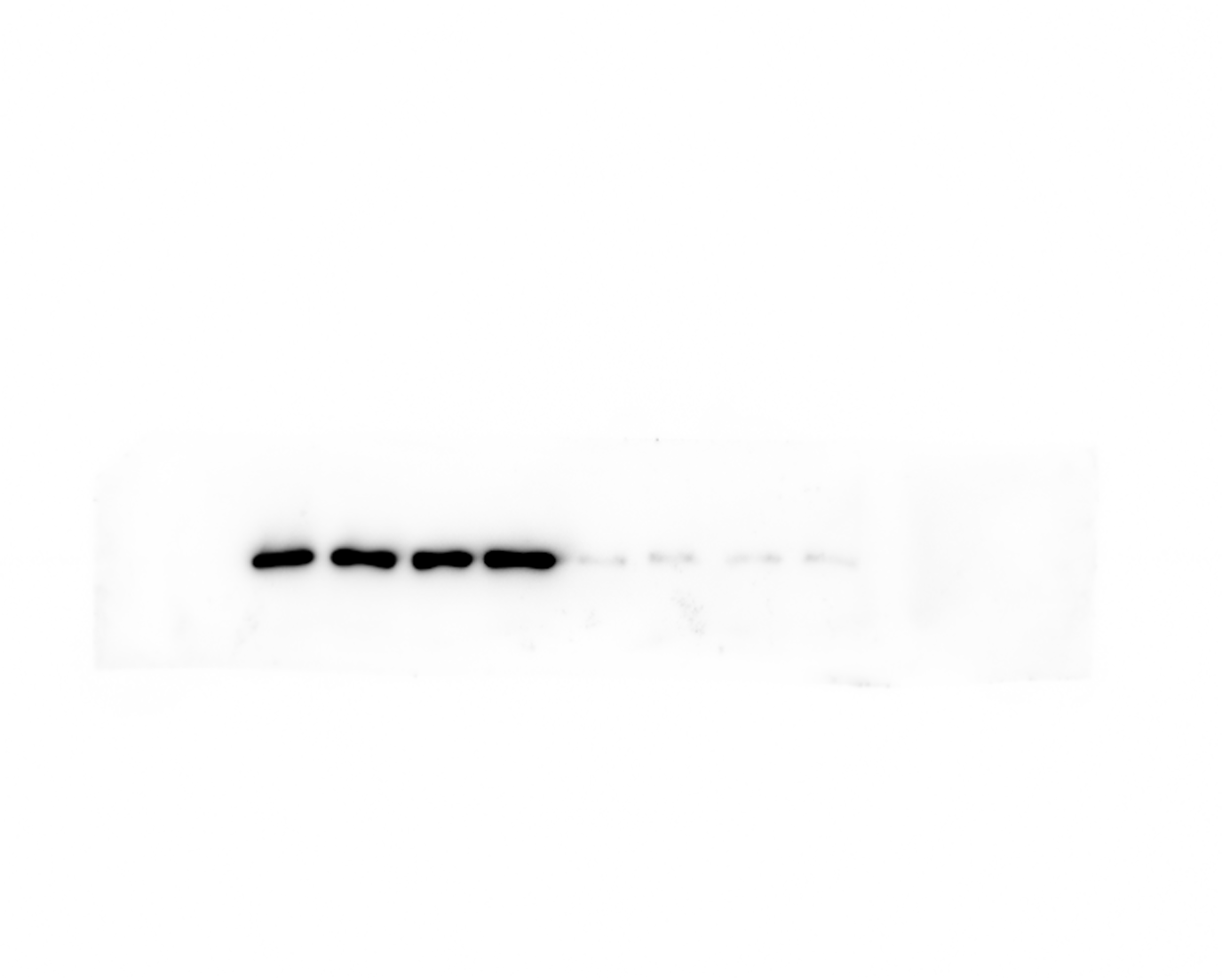

Supplement: Source data 1. [file elife-62394-data1.zip › Source data files/Source data (Raw)/Figure 4I-Source data-3(Actin).Tif]

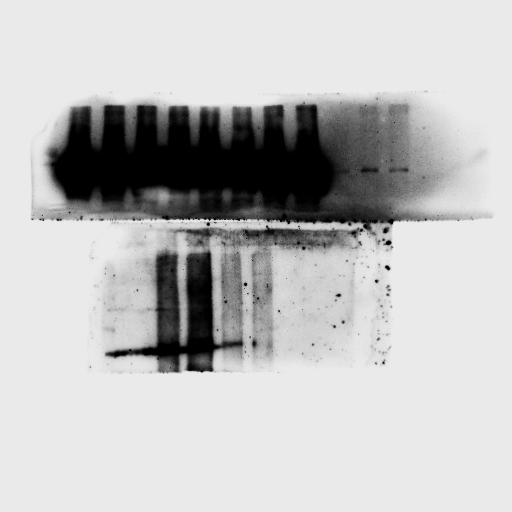

Supplement: Source data 1. [file elife-62394-data1.zip › Source data files/Source data (Raw)/Figure 5A-Source data-1(ACLY).tif]
