# Supplementary material for: ACLY ubiquitination by CUL3-KLHL25 induces the reprogramming of fatty acid metabolism to facilitate iTreg differentiation
Source: eLife. 2021 Sep 7;10:e62394. doi: 10.7554/eLife.62394 (PMC8423445; doi:10.7554/eLife.62394)
Supplement: Supplementary file 1. — Primers for PCR and quantitative PCR, and sequences of siRNAs are included. [file elife-62394-supp1.docx]

**Supplementary File 1. List of oligonucleotides**

| **Designation** | **Source or reference** | **Identifiers** | **Additional information** |
| --- | --- | --- | --- |
| *Cd4^Cre^*-F | (Dong et al., 2008) | PCR primers | AGGGTCGGAGACAATAACGG |
| *Cd4^Cre^*-R | (Dong et al., 2008) | PCR primers | TTGCGAACCTCATCACTCGT |
| WT *Cul3*-F | The Jackson Lab | PCR primers | CAGGGCTGTAATTCTGTCTGG |
| Mutant *Cul3*-F | The Jackson Lab | PCR primers | AGACTGCCTTGGGAAAAGCG |
| *Cul3*-R | The Jackson Lab | PCR primers | ATGCTCCCTACCATGCAAAC |
| *Acly*-F | (Teng et al., 2015) | RT-PCR primers | AAGAAGGAGGGGAAGCTGAT |
| *Acly*-R | (Teng et al., 2015) | RT-PCR primers | TCGCATGTCTGGGTTGTTTA |
| *Actin*-F | (Gareau et al., 2011) | RT-PCR primers | GGCTGTATTCCCCTCCATCG |
| *Actin*-R | (Gareau et al., 2011) | RT-PCR primers | GGCTGTATTCCCCTCCATCG |
| *Acly* siRNA#1-F | Suzhou GenePharma | siRNAs | GCAAAGAACUCCUGUACAATT |
| *Acly* siRNA#1-R | Suzhou GenePharma | siRNAs | UUGUACAGGAGUUCUUUGCTT |
| *Acly* siRNA#2-F | Suzhou GenePharma | siRNAs | GGAUGACAUUUCCUAUGUUTT |
| *Acly* siRNA#2-R | Suzhou GenePharma | siRNAs | AACAUAGGAAAUGUCAUCCTT |
| *Cul3* siRNA-F | (Zhang et al., 2015) | siRNAs | AUAAUUUGUACAUGCAAGCAAGGUC |
| *Cul3* siRNA-R | (Zhang et al., 2015) | siRNAs | GACCUUGCUUGCAUGUACAAAUUAU |
| *Klhl25* siRNA#1-F | Suzhou GenePharma | siRNAs | GCCGAUACUUUGAGGCCAUTT |
| *Klhl25* siRNA#1-R | Suzhou GenePharma | siRNAs | AUGGCCUCAAAGUAUCGGCTT |
| *Klhl25* siRNA#2-F | Suzhou GenePharma | siRNAs | CCGAGAUAUGGUGUCCAAATT |
| *Klhl25* siRNA#2-R | Suzhou GenePharma | siRNAs | UUUGGACACCAUAUCUCGGTT |
| *Klhl25* siRNA#3-F | Suzhou GenePharma | siRNAs | GCAGCCAGAUCUUCAUCAUTT |
| *Klhl25* siRNA#3-R | Suzhou GenePharma | siRNAs | AUGAUGAAGAUCUGGCUGCTT |
| *Cpt1* siRNA-F | (Hao et al., 2021) | siRNAs | CAUCCAUGCAUACCAAAGUTT |
| *Cpt1* siRNA-R | (Hao et al., 2021) | siRNAs | ACUUUGGUAUGCAUGGAUGTT |
| Negative control siRNA-F | Suzhou GenePharma | siRNAs | UUCUCCGAACGUGUCACGUTT |
| Negative control siRNA-R | Suzhou GenePharma | siRNAs | ACGUGACACGUUCGGAGAATT |

**References**

Dong X, Li J, Li S, Zhang J, Hua ZC. 2008. A novel genotyping strategy based on allele-specific inverse PCR for rapid and reliable identification of conditional FADD knockout mice. *Mol Biotechnol* **38**:129–135. doi:10.1007/s12033-007-9002-y

Gareau MG, Wine E, Rodrigues DM, Cho JH, Whary MT, Philpott DJ, MacQueen G, Sherman PM. 2011. Bacterial infection causes stress-induced memory dysfunction in mice. *Gut* **60**:307–317. doi:10.1136/gut.2009.202515

Hao F, Tian M, Zhang X, Jin X, Jiang Y, Sun X, Wang Y, Peng P, Liu J, Xia C, Feng Y, Wei M. 2021. Butyrate enhances CPT1A activity to promote fatty acid oxidation and iTreg differentiation. *Proc Natl Acad Sci* **118**:e2014681118. doi:10.1073/pnas.2014681118

Teng C-F, Wu H-C, Hsieh W-C, Tsai H-W, Su I-J. 2015. Activation of ATP citrate lyase by mTOR signal induces disturbed lipid metabolism in hepatitis B virus pre-S2 mutant tumorigenesis. *J Virol* **89**:605–614. doi:10.1128/jvi.02363-14

Zhang Q, Yu S, Huang X, Tan Y, Zhu C, Wang YL, Wang Haibin, Lin HY, Fu J, Wang Hongmei. 2015. New insights into the function of Cullin 3 in trophoblast invasion and migration. *Reproduction* **150**:139–149. doi:10.1530/REP-15-0126
